# Monomer‐Dependent Selectivity in Sulfur‐Containing Ring‐Opening Copolymerisation: Bimetallic Catalysis for Predictive Design of Degradable Polymers

**DOI:** 10.1002/anie.202508985

**Published:** 2025-08-26

**Authors:** Bhargav R. Manjunatha, Mani Sengoden, Merlin R. Stühler, Robert Langer, Donald J. Darensbourg, Alex J. Plajer

**Affiliations:** ^1^ Makromolekulare Chemie Universität Bayreuth Universitätsstraße 30 Bayreuth 95447 Germany; ^2^ Department of Chemistry Texas A&M University 3255 TAMU College Station TX 77843 USA; ^3^ Institute for Chemistry Martin‐Luther‐University Halle‐ Wittenberg, Kurt‐Mothes‐Str. 2 06120 Halle Germany; ^4^ Bayrisches Polymer Institut (BPI) Universität Bayreuth Universitätsstraße 30 95447 Bayreuth Germany

**Keywords:** Bimetallic catalysis, Ring opening copolymerisation, Sulfur‐containing polymers

## Abstract

Sulfur‐containing polymers offer unique opportunities for advanced materials due to their inherent degradability, high refractive indices, and potential for chemical recyclability. Yet, synthetic strategies for their precise construction remain underdeveloped and monomer selection rules to achieve this are elusive. Herein, we achieve the first comprehensive evaluation of sulfurated ring‐opening copolymerisations (ROCOP) performed under unified conditions using a single heterobimetallic Cr(III)/Rb(I) catalyst platform. Although such catalysts are largely unexplored in sulfur‐based ROCOP, our study demonstrates their remarkable synergistic performance in controlling both activity and selectivity across diverse heteroallene/epoxide systems. We identify carbonyl sulfide (COS) as the most promising monomer, enabling perfectly alternating copolymers under mild conditions on kinetic grounds. As a close second, PhNCS emerges as a viable, easier to handle, alternative that offers selective access to sulfur‐containing copolymers. In contrast, thioanhydrides and CS_2_ show progressively lower selectivity, with increasing O/S scrambling and small molecule byproduct formation. This study provides the first predictive framework linking sulfur monomer identity to selectivity and reactivity under unified conditions, enabling rational design of degradable sulfur‐rich polymers.

## Graphical abstract

A unified Cr(III)/Rb(I) catalyst was developed for sulfur‐based ring‐opening copolymerisation (ROCOP), enabling high selectivity. Perfectly alternating copolymers were obtained from COS with minimal byproducts. PhNCS was identified as a practical alternative. Lower selectivity and O/S scrambling were observed for CS_2_ and thioanhydride

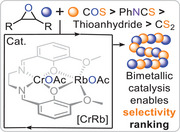

## Introduction

**Figure 1:**
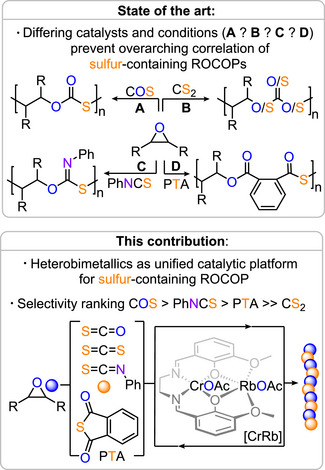
Outline of the state‐of‐the‐art and the current work.

Incorporating heteroatoms into polymer backbones enhances polymer degradability through physical, chemical, and biochemical pathways and opens avenues for innovative recycling strategies.^[^ ^1^, ^2^, ^3^ ^]^ A particularly powerful method for accessing such functional materials is the alternating ring‐opening copolymerisation (ROCOP) of heteroallenes with heterocycles, a strategy that has gained prominence for synthesizing polyesters and polycarbonates.^[^ ^4^ ^]^ More recently, this approach has been extended to a broader set of sulfur‐containing polymer classes, including polythiocarbonates from CS_2_ or COS, thiocarbamates from isothiocyanates (RNCS), and polythioesters from thioanhydrides (see Figure 1).^[^ ^5^, ^6^, ^7^, ^8^, ^9^, ^10^, ^11^, ^12^, ^13^, ^14^, ^15^, ^16^, ^17^, ^18^, ^19^, ^20^, ^21^, ^22^, ^23^, ^24^ ^]^ In this context, introducing sulfur centers into the polymer main chain can not only enhance degradability and recyclability but also introduce unique material properties such as high refractive indices and the ability to selectively coordinate transition metal centers.^[^ ^25^, ^26^, ^27^, ^28^, ^29^, ^30^, ^31^, ^32^, ^33^, ^34^, ^35^, ^36^, ^37^ ^]^ Unlike traditional oxygen‐based ROCOP systems, these emerging sulfur‐containing monomer combinations often engage in multiple propagation pathways, giving rise to a variety of isomeric linkages depending on the placement of oxygen, sulfur, and nitrogen atoms.^[^ ^38^, ^39^, ^40^, ^41^, ^42^, ^43^ ^]^ A prime example is CS_2_/epoxide ROCOP, which—while ideally producing alternating dithiocarbonate (─O─C(═S)─S─) linkages—commonly generates a mixture of carbonate and thiocarbonate structures (e.g., ─O─C(═O)─S─, ─O─C(═S)─O─, ─S─C(═S)─S─, ─O─C(═O)─O─) through a phenomenon known as O/S scrambling. This scrambling is typically accompanied by the formation of cyclic five‐membered dithiocarbonate byproducts and remains a central challenge in achieving high linkage and polymer selectivity from such monomer combinations. Mechanistic studies have revealed that O/S scrambling arises from backbiting reactions in which metal alkoxide chain‐ends attack nearby heterocarbonate linkages, yielding metal thiolate chain‐ends and oxygen‐enriched links.^[^ ^44^, ^45^ ^]^

These thiolates may propagate via further CS_2_ insertion or eliminate thiiranes, leading to complex polymer microstructures and reduced predictability. Similar scrambling and side‐product pathways have been identified in ROCOPs involving COS, RNCS, and thioanhydrides. Across all these systems, catalysis plays a defining role in determining selectivity, activity, and polymer structure. A variety of catalytic motifs have been employed, including ammonium halides, organobases, alkali metal alkoxides, and bicomponent systems pairing nucleophilic initiators with Lewis acidic boranes or metal bisiminophenoxide complexes.^[^ ^4^ ^]^ Remarkably, even seemingly simple initiator structures have been shown to exert profound effects on the ROCOP outcome—highlighting that active species formation and propagation mechanisms must be considered together.

Unfortunately though, the employed catalysts and conditions to achieve these sulfurated ROCOPs need to be tailored and optimized to each specific monomer combinations. This is not only time consuming, but also prevents mechanistic insight of how sulfurated ROCOPs correlate to each other. However, answering this question is of utmost importance to identify ROCOP monomers which are most suited to yield precision polymers with competitive and predictable material properties. Despite this necessity and interest in sulfur‐rich polymers, no general framework exists to guide monomer selection across ROCOPs due to incompatible catalytic platforms. This work fills that gap.

An emerging class of catalysts with significant yet underexplored potential in sulfur‐based ROCOP is (hetero)bimetallic complexes.^[^ ^46^, ^47^, ^48^, ^49^, ^50^, ^51^, ^52^, ^53^, ^54^, ^55^, ^56^, ^57^, ^58^, ^59^ ^]^ These systems combine synthetic accessibility with high activity at low catalyst loadings and offer well‐defined transition states that are retained across monomer types. In particular, systems pairing a transition metal (for epoxide activation) with an alkali metal (for heteroallene coordination) could offer a unique platform for mechanistically consistent studies across chemically diverse monomers. Thus, heterobimetallic catalysts are ideally suited to resolve long‐standing questions around monomer‐specific effects and selectivity trends in sulfur‐containing ROCOP.

Accordingly, we here identify a single heterobimetallic Cr(III)/Rb(I) complex as a unified catalytic platform for the ROCOP of epoxides and four sulfur‐containing comonomers—carbonyl sulfide (COS), carbon disulfide (CS_2_), phenyl isothiocyanate (PhNCS), and phthalic thioanhydride (PTA)—with epoxides. This unified approach enables a mechanistically coherent comparison of activity, selectivity, and polymer microstructure across monomers. Our results establish COS as a uniquely selective monomer, delivering perfectly alternating copolymers with high molar mass and minimal side products under mild conditions. In contrast, CS_2_, and PTA show increasing tendencies for scrambling and byproduct formation, while PhNCS emerges as a COS alternative.

## Results and Discussion

**Figure 2:**
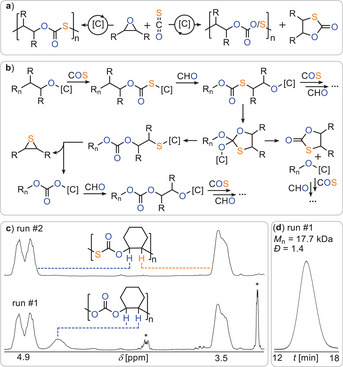
a) Comparison of reaction products for (left) perfect and (right) erroneous reaction for COS/epoxide ROCOP occurring in Table 1 run #2 and run #1, respectively. b) Propagation mechanism producing poly(monothiocarbonate) as well as side‐reactions leading to O/S scrambled links and small molecule by‐products. [C] denotes catalyst; [*R*_n_] denotes polymer chain. c) Zoom into the tertiary CH region of the ^1^H NMR spectrum of reaction products of (top) Table 1 run #2 and (bottom) run #1; *denotes cyclic small molecule byproduct d) GPC trace corresponding to run #1.

To systematically assess how the choice of sulfur‐containing heteroallene influences ROCOP performance, we employed a bis(orthomethoxy) Salen ligand scaffold, coordinating a heterobimetallic [Cr(III)OAc–Rb(I)OAc] catalyst. This catalyst, which has demonstrated superior activity and selectivity in CO_2_/epoxide ROCOP—particularly for poly(cyclohexene carbonate)—provides a robust and mechanistically consistent platform for comparative studies.^[^ ^60^ ^]^ Notably, carbonyl sulfide (COS) copolymerisations have not yet been explored using heterobimetallic catalysis. We therefore initiated the ROCOP of COS with cyclohexene oxide (CHO) at 4 bar COS pressure, 0.1 mol% catalyst loading (relative to CHO), and 100 °C (Table 1, Run #1 and Figure 2a). Analysis of the crude product by ^1^H NMR spectroscopy revealed a copolymer comprising approximately 80% monothiocarbonate linkages, evident from two equally intense secondary methine signals at *δ* ≈ 4.9 and 3.5 ppm, and 20% carbonate linkages (*δ* ≈ 4.7 ppm). The polymer was formed with 94% selectivity over the cyclic five‐membered monothiocarbonate byproduct. Gel permeation chromatography (GPC), calibrated against polystyrene standards, indicated an apparent number‐average molecular weight (*M*_n_) of 17.7 kg mol^−1^ with a dispersity of *Ð* = 1.4.

**Table 1:** COS/Epoxide ROCOP employing the [CrRb].

	[CrRb]: Epoxide	*T* (°C)	t (h)	DCM/ Tol (mL)	Conv.(%)^d)^	Pol. (%)^e)^	*M*_n_ *_,_* (kDa) (*Ð*)^f)^
#1^a)^	1:1000	100	3	–	53	94	17.7 (1.4)
#2	1:1000	50	3	–	44	>99	17.2 (1.4)
#3	1:1000	RT	24	–	45	>99	15.5 (1.4)
#4^b)^	1:1000	100	2	1	98	99	26.2 (1.3)
#5	1:1000	50	2	1	99	99	26.9 (1.3)
#6^c)^	1:1000	50	2	1	97	>99	58.6 (1.1)
#7^c)^	1:1000	RT	24	1	16	>99	12.5 (1.2)
#8^c)^	1:2000	50	2	1	97	>99	76.9 (1.2)
#9	1:2000	50	2	1	88	>99	28.0 (1.4)

Copolymerisations were carried out using 4 bar COS.

^a)^ 20% and

^b)^ 10% polycarbonate formation alongside thiocarbonate formation.

^c)^ PO was used in place of CHO.

^d)^ Relative integral of resonances from residual epoxide versus reaction products in the normalized ^1^H NMR (CDCl_3_, 400 MHz) spectrum of crude mixture.

^e)^ Relative integral in the normalized ^1^H NMR spectrum of deconvoluted resonances from small molecule byproduct versus polymer signals

^f)^ Determined by GPC (gel permeation chromatography) measurements in THF versus a narrow polystyrene standard.

The formation of carbonate linkages—formally representing an oxygen‐enriched deviation from the ideal alternating poly(monothiocarbonate) structure—raises mechanistic questions about their origin. Notably, cyclohexene sulfide was detected in the reaction mixture (see, Figure S62), implicating an O/S scrambling mechanism. Clearly, not only alternating insertion of COS and epoxides occur that produce catalyst bound monothiocarbonate and alkoxide intermediates (see Figure 2b). We propose that alkoxide‐terminated chains adjacent to thiocarbonate units undergo intramolecular attack to form metal thiolate species, which subsequently eliminate cyclohexene sulfide, generating carbonate‐terminated chains that then continue to propagate to form carbonate links. This mechanistic scenario, illustrated in Figure 2b, mirrors pathways previously proposed for CS_2_/epoxide ROCOP, but has not yet been described unambiguously for COS‐based systems.^[^ ^44^ ^]^ Lowering the polymerization temperature to 50 °C (Run #2) resulted in perfectly alternating poly(monothiocarbonate) with quantitative polymer selectivity. In contrast to the reaction at 100 °C, no formation of cyclohexene sulfide was detected in the crude mixture, confirming its origin as a side product of the O/S scrambling process. These observations support the conclusion that scrambling is thermodynamically favored, with lower temperatures suppressing the backbiting pathways that lead to thiolate formation and cyclohexene sulfide elimination. Perfect selectivity was also achieved at room temperature. However, significantly longer reaction times were required to reach comparable CHO conversion. Notably, when the reaction conditions of Run #1 were repeated with added solvent (Run #4), the extent of scrambling was substantially reduced and CHO conversion improved yielding a polymer with higher molecular weight (*M*_n_ = 26.2 kg mol^−1^, *Ð* = 1.3). This enhancement is attributed to an increased relative concentration of COS, which likely shifts the insertion equilibrium and suppresses side reactions. Encouraged by the high selectivity observed in COS/CHO ROCOP, we turned to propylene oxide (PO), an industrially relevant epoxide known for its increased propensity toward side reactions during ROCOP. Under optimized conditions (Run #6, 50 °C), COS/PO ROCOP proceeded with near‐quantitative monomer conversion and exclusive formation of alternating poly(propylene monothiocarbonate). The resulting polymer displayed a high molecular weight (*M*_n_ = 58.6 kg mol^−1^) and exceptionally narrow dispersity (*Ð* = 1.1). At room temperature, the system retained high selectivity but exhibited sluggish kinetics, yielding only low conversion after 24 h. Reducing the catalyst loading to 0.05 mol% (Run #9) further increased the molecular weight to 76.9 kg mol^−1^ (*Ð* = 1.2), demonstrating the efficiency of the CrRb system even at low catalyst concentrations.

Having established that the CrRb catalyst enables highly selective and efficient copolymerisations of COS with both CHO and PO, we next sought to evaluate its performance with other sulfur‐containing heteroallenes under comparable conditions. This assessment aimed to uncover fundamental monomer‐dependent trends and test the broader applicability of our heterobimetallic platform across challenging sulfur‐containing ROCOP systems.

**Figure 3:**
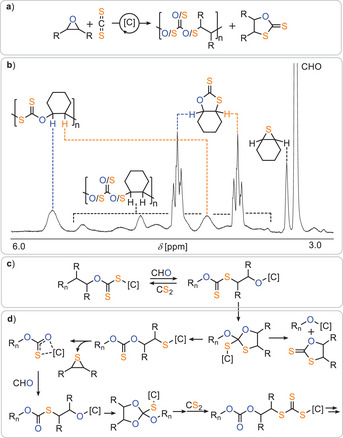
a) Mixed reaction products for CS_2_/epoxide ROCOP occurring under all investigated conditions. b) ^1^H NMR spectrum (CDCl_3_) of product mixture produced at 1 eq. CrRb:1000 eq. CHO: 1000 eq. CS_2_ and 50 °C. c) Main propagation cycling producing dithiocarbonate links. d) Side‐reactions leading to O/S scrambled links and small molecule by‐products.

We next turned to the ROCOP of carbon disulfide (CS_2_) with epoxides to directly compare its reactivity and selectivity with that of COS under identical heterobimetallic catalysis (see Figure 3a). From the outset, CS_2_/CHO ROCOP proved considerably more challenging. Whereas COS ROCOP tolerated, and even benefitted from, the presence of co‐solvent, CS_2_ ROCOP was entirely suppressed under otherwise identical conditions—no CHO turnover was observed. Furthermore, unlike COS, CS_2_ showed no reactivity at room temperature, even under neat conditions. At 50 °C, under solvent‐free conditions with equimolar CHO and CS_2_ (1 equiv. CrRb:1000 equiv. CHO:1000 equiv CS_2_), only partial monomer conversion (57%) was achieved after 20 h, and selectivity was markedly diminished. Analysis of the crude product mixture revealed that the major product was the cyclic dithiocarbonate (63%), while only a minor fraction (37%) constituted polymeric material. Moreover, the polymer was heavily scrambled, with ^1^H and ^13^C NMR spectra indicating a mixture of carbonate (─O─C(═O)─O─), thionocarbonate (─O─C(═S)─O─), and trithiocarbonate (─S─C(═S)─S─) linkages, with only a minor fraction of the ideal alternating dithiocarbonate (─O─C(═S)─S─) motif (see Figure 3b). Cyclohexene sulfide was also detected in the reaction mixture, consistent with a backbiting‐mediated O/S scrambling mechanism that occurs in addition to alternating propagation (see Figure 3c,d) in agreement with previous reports.^[^ ^44^ ^]^ In addition to reduced selectivity, the polymer showed a significantly lower molecular weight (*M*_n_ = 10.4 kg mol^−1^) and broader dispersity (*Ð* = 1.7) compared to COS‐based polymers under analogous conditions (e.g., *M*_n_ = 17.2 kg mol^−1^, *Ð* = 1.4 for COS/CHO; Table 1, Run #2). Attempts to improve CS_2_ ROCOP outcomes by increasing temperature or extending reaction time resulted in further deterioration of selectivity and polymer quality (see, Table S4). CS_2_/PO ROCOP followed similar trends: no polymerization occurred at room temperature, regardless of solvent presence, and the best outcome was achieved at 50 °C over 20 h, yielding a copolymer with 60% selectivity, low molecular weight (*M*_n_ = 13.2 kDa), and significant O/S scrambling. Cyclic trithiocarbonate and other low‐molecular‐weight byproducts were again predominant (see, Figure S19) Taken together, these results clearly indicate that CS_2_ ROCOP is inherently more prone to side reactions—including O/S scrambling and cyclic byproduct formation—than its COS analogue. The greater scrambling and lower selectivity suggest that CS_2_ is both kinetically and thermodynamically more susceptible to competitive chain‐end transformations, limiting its utility for generating sequence‐defined sulfur‐containing polymers under the current catalytic regime.

**Figure 4:**
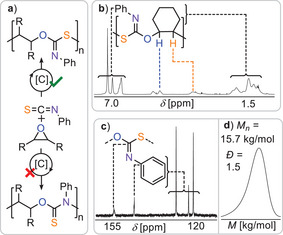
a) Synthetic scheme for PhNCS/epoxide ROCOP. b) ^1^H, c) ^13^C NMR (CDCl_3_) and d) GPC curve of isolated polymer produced at 1 eqv. CrRb:1000 eqv. CHO: 1000 eqv. PhNCS at 100 °C after 24 h.

Next, we investigated the ROCOP of phenyl isothiocyanate (PhNCS) with epoxides, wherein the oxygen atom of COS is formally replaced with a PhN group (see Figure 4). Using a 1:1000:1000 ratio of CrRb:CHO:PhNCS, the overall reactivity was again found to be lower than for COS. At room temperature, no detectable monomer turnover was observed. Upon increasing the temperature to 50 °C, CHO conversion reached 57% after 20 h, yielding copolymer with 87% selectivity. These values are comparable to those obtained for CS_2_ in terms of activity, though polymer selectivity is improved. However, both parameters remain lower than those achieved with COS under identical conditions. Strikingly, raising the temperature to 100 °C significantly enhanced the performance of PhNCS/CHO ROCOP. After 24 h, CHO conversion reached 83%, with 96% polymer selectivity, approaching the selectivity seen for COS and substantially outperforming CS_2_. The resulting polymer exhibited an apparent number‐average molecular weight of *M*_n_ = 15.7 kDa and a dispersity of *Ð* = 1.5. ^13^C NMR analysis revealed exclusive formation of monothioimidocarbonate linkages (─O─C(═NPh)─S─), indicated by a distinct quaternary resonance at *δ* = 155.7 ppm. Importantly, no signals were observed at ∼188 ppm, which would correspond to the isomeric ─O─C(═S)─NPh─ linkages, indicating complete regioselectivity.^[^ ^21^ ^]^ In ROCOP of PhNCS with propylene oxide (PO), reactivity trends paralleled those observed with CHO. No polymerization occurred at room temperature. At 50 °C, 83% PO conversion was achieved after 20 h, yielding polymer in 90% selectivity with a high *M*_n_ = 45.3 kg mol^−1^ and *Ð* = 1.3 substantially outperforming CS_2_. Increasing the temperature to 100 °C accelerated the reaction to 87% conversion in just 3 h but produced a polymer of slightly lower molecular weight (*M*_n_ = 32.0 kg mol^−1^) and broader dispersity (*Ð* = 1.5). Again, ^13^C NMR confirmed exclusive formation of monothioimidocarbonate (─O─C(═NPh)─S─) linkages, with a quaternary carbon resonance at *δ* = 155.9 ppm, confirming high chemoselectivity of PhNCS ROCOP across both epoxide substrates.

**Figure 5:**
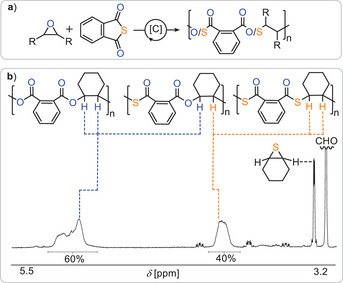
a) Synthetic scheme for PTA/epoxide ROCOP. b) ^1^H NMR spectrum (CDCl_3_) of product mixture produced at 1 eq. CrRb:1000 eq. CHO: 1000 eq. PTA and 100 °C.

Finally, we investigated the ROCOP of phthalic thioanhydride (PTA) with epoxides (see Figure 5). Although PTA is not a heteroallene, it follows a mechanistically analogous alternating insertion pathway during copolymerisation with epoxides. As with other comonomers tested, PTA/CHO ROCOP did not proceed at room temperature under the conditions employed (1 equiv. CrRb:1000 equiv. CHO:1000 equiv. PTA). At 50 °C, 26% CHO conversion was achieved after 24 h, yielding a copolymer with *M*_n_ = 10.2 kDa and *Ð* = 1.2. ^13^C NMR analysis indicated the formation of approximately equal proportions of ester and thioester linkages. However, the microstructure was scrambled, with diester and dithioester motifs forming alongside the desired alternating ester–thioester sequence. Due to overlapping resonances, a precise quantification of individual linkage types was not possible. In contrast, under comparable conditions, COS ROCOP delivered perfectly alternating polymers at higher rates and greater molar mass, while with PhNCS high sequence selectivity was obtained. Increasing the temperature to 100 °C significantly improved the PTA/CHO reaction rate, with 74% monomer conversion after 24 h and a polymer of *M*_n_ = 14.5 kDa (*Ð* = 1.7). However, this was accompanied by increased scrambling, favoring ester‐enriched linkages. Mechanistically, this is attributed to thiirane elimination, a pathway that converts alternating sequences into diester motifs. Consistent with this, cyclohexyl sulfide was detected in the crude product mixture. Similar behavior was observed in PTA/PO ROCOP. At room temperature, only minimal turnover (6%) occurred after 24 h. At 50 °C, a copolymer comprising a mixture of diester, dithioester, and ester–thioester links was formed, with *M*_n_ = 42.0 kDa and *Ð* = 1.5. Under these same conditions, COS/PO ROCOP yielded a perfectly alternating polymer. Interestingly, the ^13^C NMR spectrum of the PTA/PO polymer displayed fewer quaternary carbon resonances in the 150–200 ppm region compared to PTA/CHO, suggesting that PO is less prone to scrambling reactions than CHO in this system. Integration of the diagnostic ^13^C signals revealed that the alternating ester–thioester motif constituted 64% of the total linkage population—a quantification method we previously validated for this class of polymers.^[^ ^39^ ^]^ At elevated temperatures (100 °C), ROCOP rates increased further, but again at the expense of molecular weight and microstructural integrity (see, Table S1 Runs #7–#9).

Comparing the results from the previous sections summarized in Table 2 (run #1‐ #8), let us formulate clear guidelines for sulfur‐containing ROCOP. COS is the most active and linkage‐selective comonomer, delivering perfectly alternating copolymers with near‐quantitative polymer selectivity and the highest molecular weights. PhNCS also performs well, offering high selectivity and molecular weights—particularly with PO—and comparable thermal properties. While slightly less efficient than COS, PhNCS is more practical to handle due to its liquid state compared to gaseous COS, facilitating application. In contrast, CS_2_ leads to extensive scrambling and low polymer content, while PTA shows moderate performance with increased side reactions at elevated temperatures. Overall, COS sets the benchmark, but PhNCS emerges as a viable and user‐friendly alternative for precision sulfur polymer synthesis.

**Table 2:** Comparison of sulfurated ROCOPs.

Run	Monomers	*T* (°C)	*t* (h)	Conv. (%)^a)^	TOF (h^−1^)^b)^	Polymer (%)^c)^	Alternation (%)^d)^	*M*_n,GPC_ (kg/mol) (*Ð*)^e)^	*T*_d,5%_ (°C)	*T*_g_ (°C)^f)^
#1	COS/PO	50	2	97	485	99	99	76.9 (1.2)	201.9	25.4
#2	CS_2_/PO	50	20	99	50	60	16	13.2 (1.7)	151.2	17.9
#3	PhNCS/PO	50	20	83	42	90	>95	45.3 (1.3)	165.8	61.6
#4	PTA/PO	50	24	92	38	99	64	42.0 (1.5)	262.2	69.2
#5	COS/CHO	50	2	88	440	99	99	28.0 (1.4)	230.0	102.6
#6	CS_2_/CHO	50	20	57	29	37	41	10.4 (1.7)	159.6	88.4
#7	PhNCS/CHO	100	24	83	35	98	>95	15.7 (1.5)	198.4	95.5
#8	PTA/CHO	100	24	74	31	99	54	14.5 (1.7)	269.0	135.6
#9	COS/PGE	50	2	99	495	99	99	43.9 (1.4)	230.6	45.7
#10	CS_2_/PGE	50	24	72	30	25	36	5.4 (2.0)	171.7	34.6
#11	PhNCS/PGE	50	24	26	11	>95	>95	17.6 (1.3)	215.3	42.6
#12	PTA/PGE	50	24	38	16	99	59	17.3 (1.5)	282.3	65.4
#13	PhNCS/EGE	50	24	69	29	99	>95	19.4 (1.2)	197.4	14.8
#14	PhNCS/DO	50	24	42	18	97	>95	28.9 (1.2)	190.1	−12.4

Copolymerisations were carried out using [CrRb] (1 equiv.), CHO or PO (1000 equiv.), and 4 bar COS (with 1 mL cosolvent) or 1000 equiv. comonomer and runs presented in the Table were selected from Table S1 to S7 of the supporting information that yielded highest *M*_n_ polymers.

^a)^ Relative integral of resonances from residual epoxide versus reaction products in the normalized ^1^H NMR (CDCl_3_, 400 MHz) spectrum of crude mixture.

^b)^ Turn over frequency as in equivalents of consumed epoxide per equivalent of catalyst per hour.

^c)^ Relative integral in the normalized ^1^H NMR spectrum of deconvoluted resonances from small molecule byproduct versus polymer signals.

^d)^ Relative integrals in the ^13^C NMR spectrum (CDCl_3_, 126 MHz) from carbonyl resonances due to alternating links compared to other carbonyl resonances.

^e)^ Determined by GPC (gel permeation chromatography) measurements in THF versus a narrow polystyrene standard.

^f)^ Glass transition temperature *T*_g_ determined from the second heating cycle by differential scanning calorimetry. *T*_d,5%_ degradation temperature at 5% polymer degradation determined by thermogravimetric analysis (TGA)

To confirm that our guidelines hold true for other epoxides we turned to the ROCOP of phenyl glycidylether (PGE) (see Table 2 run #9 ‐ #12). COS ROCOP again delivers perfectly alternating copolymer in highest molecular weight, followed by PhNCS ROCOP yielding reduced molecular weights at albeit near perfect selectivity and excellent polymer yield. PTA produces a more scrambled copolymer in quantitative yield, while employing CS_2_ results in predominantly small molecule by‐products. Finally demonstrating the utility of PhNCS ROCOP, we employed epoxides with flexible side chains, i.e., ethyl glycidyl ether (EGE) and decyl oxide (DO), modulating thermal properties (run #13 and #14). Indeed sulfur‐containing copolymers in excellent selectivity were obtained, which, in contrast to the PhNCS copolymers investigated so far with PO and CHO, show glass transitions below room‐temperature.

Furthermore, our comparative study provides unique insights into the degradability of sulfur‐containing ROCOP copolymers. In terms of thermal stability, PTA‐based copolymers consistently exhibit the highest degradation onset temperatures (*T*_d,5%_), followed by COS and PhNCS copolymers, while CS_2_‐derived materials show the lowest thermal stability, and these trends can be observed regardless of the epoxide comonomer employed. Preliminary hydrolysis studies on CHO‐based copolymers were conducted by monitoring weight loss after dispersion in aqueous media at 40 °C over 7 days (see ESI Section S4). Under acidic conditions, all copolymers showed modest degradation (5–26 wt%). In basic media, more pronounced degradation was observed for COS, PTA, and CS_2_ copolymers (42–62 wt%), whereas the PhNCS copolymer remained completely stable. Aminolysis using 7 M ammonia in methanol proved to be the most effective degradation route, resulting in 82–99 wt% degradation for COS, PTA, and CS_2_ copolymers, while the PhNCS‐derived polymer again remained unaffected.

**Figure 6:**
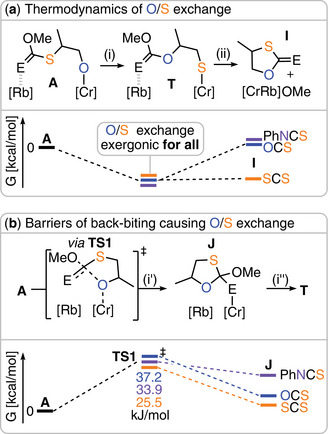
a) Energetics of O/S exchange reaction and small molecule by‐product formation for ROCOP of PO with different heteroallenes (E = S for CS_2_, E = O for COS, and E = NPh for PhNCS). b) Kinetic barriers of back‐biting reaction representing the central initial step of the O/S exchange reaction; note that the structure of **J** changes depending on E as described in the main text.

To elucidate the origins of the observed selectivity trends, we turned to density functional theory (DFT) and computed the relative Gibbs free energies for i) O/S exchange (thiolates **T** relative to alkoxides **A**) and ii) the formation of cyclic small‐molecule byproducts (II), using Gaussian16 at the B97D3/def2‐TZVP level of theory (see Figure 6a). The structures of the intermediates were modelled based on established coordination modes of bimetallic ROCOP catalysts, considering both the chain‐end and the adjacent polymer linkage.^[^ ^44^, ^61^ ^]^ For computational efficiency PO ROCOP was investigated and the polymer chain was abbreviated as Ph in the case of PTA ROCOP and as OMe for heteroallene ROCOP.

Surprisingly, O/S exchange in the gas phase was found to be thermodynamically favorable for all monomers investigated, with relative Gibbs energies of –6.3 kcal mol^−1^ (CS_2_), –7.5 kcal mol^−1^ (PhNCS), –8.7 kcal mol^−1^ (COS), and –33.6 kcal mol^−1^ (PTA). The formation of cyclic five‐membered byproducts (**I** in (ii)) was calculated to be slightly endergonic for COS (Δ*G* = +1.4 kcal mol^−1^) and PhNCS (Δ*G* = +1.7 kcal mol^−1^), while for CS_2_, elimination of cyclic dithiocarbonates was thermodynamically favorable (Δ*G* = –6.2 kcal mol^−1^). These results suggest that the degree of alternation in COS, CS_2_, and PhNCS copolymers is primarily governed by kinetic factors, whereas PTA exhibits a stronger thermodynamic drive toward scrambling. For the formation of small molecule cyclic byproducts, a clear thermodynamic tendency is revealed, in line with experimental results, where this pathway is most pronounced for CS_2_.

The O/S exchange process occurring in (i) comprises two separate events (see Figure 6b). In (i′), the Cr‐bound alkoxide **A** back‐bites into the neighboring carbonyl atom via **TS1** to form a cyclic intermediate **J**, which then collapses in (ii′) to **T**. Hence, **TS1** represents the kinetic barrier to entering the O/S scrambling process. In the case of COS, the Gibbs free energy barrier for **TS1** relative to **A** (Δ*G* ^‡^ = 37.2 kcal mol^−1^) was found to be substantially higher than for PhNCS (Δ*G* ^‡^ = 33.9 kcal mol^−1^) and CS_2_ (Δ*G* ^‡^ = 25.5 kcal mol^−1^), clearly indicating a kinetic preference for back‐biting in the reaction with CS_2_. The nucleophilic attack results in a cyclic intermediate **J**, which in the case of CS_2_ (Δ*G* = 6.5 kcal mol^−1^) is coordinated via a sulfur center, and in the case of COS (Δ*G* = 11.9 kcal mol^−1^) is coordinated via an oxygen center. The higher stability of the sulfur‐bound intermediate in the CS_2_ reaction may be due to the relatively weak C═S double bond, which is converted into a single bond upon nucleophilic attack. For PhNCS, coordination via the resulting amido nitrogen atom was not favorable; instead, an intermediate is formed (Δ*G* = 32.2 kcal mol^−1^) in which the chromium atom is coordinated by the oxygen atom of the MeO substituent. Combined, our computational results indicate that although O/S scrambling is thermodynamically favorable regardless of the sulfur‐containing comonomer, it is governed kinetically. To avoid scrambling, the barrier for back‐biting of the chain end into a neighboring polymer link must be maximized—this occurs most efficiently with COS.

## Conclusion

In conclusion, we present the first systematic and mechanistically unified comparison of sulfur‐containing ring‐opening copolymerisations (ROCOP) using a single heterobimetallic Cr(III)/Rb(I) catalyst. By investigating COS, CS_2_, PhNCS, and PTA under identical conditions, we reveal striking monomer‐dependent trends in activity, selectivity, and polymer microstructure. COS emerges as the benchmark monomer, enabling perfectly alternating, high‐molecular‐weight copolymers with minimal side reactions—even at low catalyst loadings. PhNCS, while slightly less reactive, offers similarly high selectivity and practical advantages as a liquid monomer. Other monomers exhibit extensive O/S scrambling and reduced polymer fidelity, limiting their utility for precision polymer synthesis which is most pronounced for CS_2_. These findings establish heterobimetallic catalysis as a broadly applicable platform for selective sulfur‐containing polymer synthesis and offer a foundational guide for monomer selection in next‐generation sustainable polymer materials.

## Conflict of Interests

The authors declare no conflict of interest.

## Supporting information



Supporting Information

## Data Availability

The data that support the findings of this study are available in the supplementary material of this article.
