# Supplementary material for: Monomer‐Dependent Selectivity in Sulfur‐Containing Ring‐Opening Copolymerisation: Bimetallic Catalysis for Predictive Design of Degradable Polymers
Source: Angew Chem Int Ed Engl. 2025 Aug 26;64(42):e202508985. doi: 10.1002/anie.202508985 (PMC12518690; doi:10.1002/anie.202508985)
Supplement: Supplementary file 1 — Supporting Information [file ANIE-64-e202508985-s001.pdf]

Electronic Supporting Information for

Monomer-Dependent Selectivity in Sulfur-Containing Ring-Opening Copolymerisation: Bimetallic Catalysis for Predictive Design of Degradable Polymers

Bhargav R. Manjunatha,[a] Mani Sengoden,[b] Merlin R. Stühler,[a] Robert Langer,[c]

Donald J. Darensbourg [b,*] Alex J. Plajer [a,d*]

[a] Makromolekulare Chemie 1, Universität Bayreuth, Universitätsstraße 30, 95447, Bayreuth Germany; *email: alex.plajer@uni-bayreuth.de; [b] Department of Chemistry, Texas A&M University, 3255 TAMU, College Station, TX, 77843 USA *email: djdarens@chem.tamu.edu; [c]Institute for Chemistry, Martin-Luther-University Halle- Wittenberg, Kurt-Mothes-Str. 2, 06120 Halle; [d]Bayrisches Polymer Institut (BPI), Universität Bayreuth, Universitätsstraße 30, 95447 Bayreuth.

Table of Contents

Section S1: ROCOP tables from different monomer combinations **3**

Section S2: General methods **10**

Section S3: Polymer characterisation **11**

a. **PTA/PO ROCOP** **11**
b. **PTA/CHO ROCOP** **15**
c. **PTA/PGE ROCOP** **19**
d. **CS_2/PO ROCOP** **23**
e. **CS_2/CHO ROCOP** **25**
f. **CS_2/PGE ROCOP** **27**
g. **PhNCS/epoxide ROCOP** **31**
h. **COS/epoxide ROCOP** **51**

Section S4: Degradation studies **64**

Section S5: Computational details **65**

Section S6: References **65**

Section S1: ROCOP tables of different monomer combinations

Table S1: PTA/PO ROCOP

No.	Temp. (°C)	Time (h)	PTA conv. (%) [a]	Selectivity (%) [b]	M_n ($Đ$) [c] (kg/mol)
1	30	2	0	-	-
2	30	4	0	-	-
3	30	24	6	91	7.6 (1.20)
4	50	2	6	92	6.4 (1.12)
5	50	4	8	92	7.1 (1.25)
**6**	**50**	**24**	**92**	**92**	**42.0 (1.46)**
7	100	2	76	96	20.6 (1.70)
8	100	4	92	80	19.2 (1.76)
9	100	24	>99	64	25.7 (1.84)
10#	100	24	>99	72	23.1 (1.84)

Copolymerization was carried out using [CrRb] cat. (3.0 mg, 1 eq., 5.16 µmol), PTA (0.848 g, 1000 eq., 5.16 mmol) and PO (0.361 mL, 1000 eq., 5.16 mmol). **#** 1500 eq. PO. **[a]** Relative integral of aromatic resonances from residual PTA versus polymer in the normalised ^{1}H NMR ($CDCl_3$) spectrum of crude polymer. **[b]** Selectivity (%) determined by relative integral of ester/thioester vs. ether/thioether resonances in normalised ^{1}H NMR spectrum of the isolated polymer. **[c]** Determined by GPC measurements in THF calibrated against a narrow polystyrene standard. $Đ = M_w / M_n$.

Table S2: PTA/CHO ROCOP

No.	Temp. (°C)	Time (h)	PTA conv. (%) [a]	Selectivity (ester: thioester) [b]	M_n ($Đ$) [c] (kg/mol)
1	30	2	0	-	-
2	30	4	0	-	-
3	30	24	-	-	-
4	50	2	0	-	-
5	50	4	4	53:47	4.8 (1.09)
6	50	24	26	53:47	10.2 (1.36)
7#	50	24	24	53:47	8.5 (1.32)
8	100	2	24	61:39	8.4 (1.26)
9	100	4	38	63:37	9.2 (1.36)
**10**	**100**	**24**	**74**	**60:40**	**14.5 (1.69)**
11#	100	24	92	63:37	14.2 (1.67)

Copolymerization was carried out using [CrRb] cat. (3.0 mg, 1 eq., 5.16 µmol), PTA (0.848 g, 1000 eq., 5.16 mmol) and CHO (0.522 mL, 1000 eq., 5.16 mmol). **#** 1500 eq. CHO. **[a]** Relative integral of aromatic resonances from residual PTA versus polymer in the normalised ^{1}H NMR ($CDCl_3$) spectrum of crude polymer. **[b]** Relative integral of ester vs. thioester resonances in normalised ^{1}H NMR spectrum of the isolated polymer. **[c]** Determined by GPC measurements in THF calibrated against a narrow polystyrene standard. $Đ = M_w / M_n$.

Table S3: CS_2/PO ROCOP

No.	Temp. (°C)	Time (h)	PO conv. (%) [a]	Selectivity (%) vs. cyc. bp. [b]	M_n ($Đ$) [c] (kg/mol)
1	30	2	5	--	**--**
2	30	3	3	-	**--**
3	30	24	0	--	**--**
4	50	2	10	42	**--**
5	50	3	4	--	**--**
**6**	**50**	**20**	**>99**	**60**	**13.2 (1.69)**
7	50	24	>99	55	9.5 (1.61)
8	100	2	>99	5	--
9	100	3	>99	6	--
10	100	24	>99	--	3.5 (1.62)

Copolymerization was carried out using [CrRb] cat. (3.0 mg, 1 eq., 5.16 μmol), CS_2 (0.310 mL, 1000 eq., 5.16 mmol) and PO (0.361 mL, 1000 eq., 5.16 mmol). **[a]** Relative integral of methyl resonances from residual PO versus polymer in the normalised ^{1}H NMR ($CDCl_3$) spectrum of crude polymer. **[b]** Selectivity (%) determined by relative integral of -C*H* resonances from PO in polymer vs. cyclic by-products in normalised ^{1}H NMR spectrum of the crude polymer. **[c]** Determined by GPC measurements in THF calibrated against a narrow polystyrene standard. $Đ = M_w / M_n$.

Table S4: CS_2/CHO ROCOP

No.	Temp. (°C)	Time (h)	CHO conv. (%) [a]	Selectivity (%) vs. bp. [b]	M_n ($Đ$) [c] (kg/mol)
1	30	2	0	--	--
2	30	3	0	--	--
3	30	24	0	--	--
4	50	2	2	--	--
5	50	3	8	--	--
**6**	**50**	**20**	**57**	**37**	**10.4 (1.74)**
7	50	24	84	33	7.9 (1.53)
8	100	2	>99	21	6.2 (1.66)
9	100	3	>99	18	4.8 (1.61)
10	100	24	>99	0	--

Copolymerization was carried out using [CrRb] cat. (3.0 mg, 1 eq., 5.16 µmol), CS_2 (0.310 mL, 1000 eq., 5.16 mmol) and CHO (0.522 mL, 1000 eq., 5.16 mmol). **[a]** Relative integral of -*H*C-C*H*- resonances from residual CHO vs. polymer & by-products in the normalised ^{1}H NMR ($CDCl_3$) spectrum of crude polymer. **[b]** Selectivity (%) determined by relative integral of -*H*C-C*H*- resonances adjacent to ester/thioester in polymer vs. by-products in normalised ^{1}H NMR spectrum of the crude polymer. **[c]** Determined by GPC measurements in THF calibrated against a narrow polystyrene standard. $Đ = M_w / M_n$.

Table S5: PhNCS/PO ROCOP

No.	Temp. (°C)	Time (h)	PO conv. (%) [a]	Selectivity (%) vs. cyc. bp. [b]	M_n ($Đ$) [c] (kg/mol)
1	30	2	0	--	--
2	30	3	0	--	--
3	30	24	0	--	--
4	50	2	0	--	--
5	50	3	4	--	--
**6**	**50**	**20**	**83**	**90**	**45.3 (1.26)**
7	50	24	83	83	39.5 (1.21)
8	100	0.5	29	64	8.96 (1.27)
9	100	2	56	83	25.8 (1.48)
10	100	3	87	87	32.0 (1.46)
11	100	24	94	79	10.3 (1.57)

Copolymerization was carried out using [CrRb] cat. (3.0 mg, 1 eq., 5.16 μmol), PhNCS (0.618 mL, 1000 eq., 5.16 mmol) and PO (0.361 mL, 1000 eq., 5.16 mmol). **[a]** Relative integral of methyl resonances from residual PO vs. polymer in the normalised ^{1}H NMR ($CDCl_3$) spectrum of crude polymer. **[b]** Selectivity (%) was determined by relative integral of -C*H* resonances from PO in polymer vs. cyclic by-products in normalised ^{1}H NMR spectrum of the crude polymer. **[c]** Determined by GPC measurements in THF calibrated against a narrow polystyrene standard. $Đ = M_w / M_n$.

Table S6: PhNCS/CHO ROCOP

No.	Temp. (°C)	Time (h)	CHO conv. (%) [a]	Selectivity (%) vs. bp. [b]	M_n ($Đ$) [c] (kg/mol)
1	30	2	0	--	--
2	30	3	0	--	--
3	30	24	2	--	3.0 (1.12)
4	50	2	0	--	--
5	50	3	4	--	--
6	50	20	30	83	8.6 (1.55)
7	50	24	57	87	12.6 (1.49)
8	100	0.5	26	71	6.36 (1.46)
9	100	2	37	98	13.43 (1.45)
10	100	3	34	98	14.3 (1.38)
**11**	**100**	**24**	**83**	**96**	**15.7 (1.52)**

Copolymerization was carried out using [CrRb] cat. (3.0 mg, 1 eq., 5.16 μmol), PhNCS (0.618 mL, 1000 eq., 5.16 mmol) and CHO (0.522 mL, 1000 eq., 5.16 mmol). **[a]** Relative integral of -*H*C-C*H*- resonances from residual CHO vs. polymer & by-products in the normalised ^{1}H NMR ($CDCl_3$) spectrum of crude polymer. **[b]** Selectivity (%) determined by relative integral of -*H*C-C*H*- resonances adjacent to ester/thioester in polymer vs. by-products in normalised ^{1}H NMR spectrum of the crude polymer. **[c]** Determined by GPC measurements in THF calibrated against a narrow polystyrene standard. $Đ = M_w / M_n$.

Table S7: Copolymerization of PhNCS with different epoxides

Epoxides	Temp. (ºC)	Time (h)	Conv. (%) [a]	TOF (h^{-1}) [b]	Polymer (%) [c]	Alternation (%) [d]	M_n ($Đ$) [e] (kg/mol)	$T_{d,5\%}$ (°C)	T_g (ºC) [f]
**PO**	50	20	83	42	90	>95	45.3 (1.3)	165.8	61.6
**CHO**	100	24	83	35	98	>95	15.7 (1.5)	198.4	95.5
**PGE**	50	24	26	11	>95	>95	17.6 (1.3)	215.3	42.6
**EGE**	50	24	69	29	99	>95	19.4 (1.2)	197.4	14.8
**DO**	50	24	42	18	97	>95	28.9 (1.2)	190.1	-12.4

Copolymerizations were carried out using [CrRb] (1 equiv.), epoxide (1000 equiv.) and PhNCS (1000 equiv.) comonomer. **[a]** Relative integral of resonances from residual epoxide versus reaction products in the normalised ^{1}H NMR ($CDCl_3$) spectrum of crude mixture. **[b]** Turn over frequency as in equivalents of consumed epoxide per equivalent of catalyst per hour. **[c]** Relative integral in the normalised ^{1}H NMR spectrum of deconvoluted resonances from small molecule byproduct versus polymer signals. **[d]** Relative integrals in the ^{13}C NMR spectrum (CDCl3, 126 MHz) from carbonyl resonances due to alternating links compared to other carbonyl resonances. **[e]** Determined by GPC (gel permeation chromatography) measurements in THF versus a narrow polystyrene standard. **[f]** Glass transition temperature T_g determined from the second heating cycle by differential scanning calorimetry. $T_{d,5\%}$ Degradation temperature at 5% polymer degradation determined by thermogravimetric analysis (TGA). $Đ = M_w / M_n$.

Section S2: General methods

Solvents and reagents were obtained from commercial sources and used as received unless stated otherwise. If “dried solvents” were used these were obtained by different procedures. Propylene oxide (PO), carbon disulfide (CS_2) were dried with calcium hydride before distillation, then degassed and stored in a glove box filled with argon. Phthalic thioanhydride (PTA) was synthesised according to the literature.[[1]] Phenyl isothiocyanate (PhNCS) and cyclohexene oxide (CHO) were dried with calcium hydride, vacuum distilled and stored in the glovebox. [CrRb] was synthesized according to literature.[1]

Nuclear Magnetic Resonance (NMR) spectra were recorded by using Bruker Advance 300 and 500 MHz spectrometers. ^1H and $^{13}C\{^1H\}$ chemical shifts are referenced to the residual proton resonance of the deuterated solvents. Thermogravimetric Analysis (TGA) data was measured using a Mettler Toledo STAR^e System ‘’TGA/DSC 3+’’. Differential scanning calorimetry (DSC) was measured on a Mettler Toledo ‘’STAR^e System DSC 3+’’ at a heating rate of 10.0 K/min. The molecular weight and polydispersity of the polymers were determined by a Waters 515 Gel permeation chromatography (GPC) instrument equipped with two linear PLgel columns (Mixed-C) following guard column and a differential refractive index detector using tetrahydrofuran as the eluent at a flow rate of 1.0 mL/min at 30 °C and a series of narrow polystyrene standards for the calibration of the columns. Each polymer sample was dissolved in HPLC-grade THF (2 mg/mL) and filtered through a 0.20 µm porous filter frit prior to analysis.

General Polymerization Procedure:

The appropriate amount of catalyst (1 eq., 5.16 µmol, 3.0 mg) and the monomers (1000 eq., 5.16 mmol each) were added to an oven dried vial equipped with a magnetic stirrer and sealed with a melamine cap containing a Teflon inlay inside an argon filled glovebox. The vial was then brought outside the glovebox and placed in a pre-heated aluminium block at the specified temperature for the specified time. At the specified end point, the polymerisation mixture was cooled down to ambient temperature, an aliquot was removed and analysed by ^1H NMR for the determination of conversion. The mixture was then dissolved in 5 mL DCM and added dropwise to 40 mL of MeOH causing precipitation of the polymer, which was then isolated by centrifugation. This precipitation was repeated twice, and the obtained polymer was dried in a vacuum for 24 h oven before further analysis.

Section S3: Polymer Characterisation

a. PTA/PO ROCOP

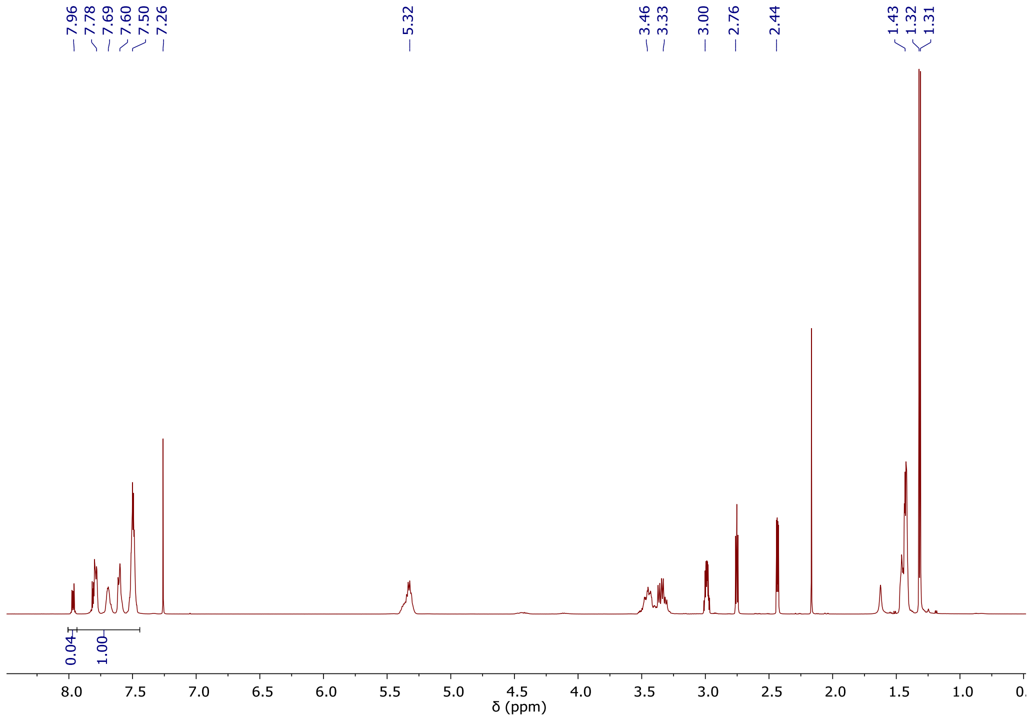

Figure S 1: ^{1}H NMR spectrum (500 MHz, $CDCl_3$) of the final aliquot from PTA/PO ROCOP (table S1, run 6). Peak assignments according to literature. [2]

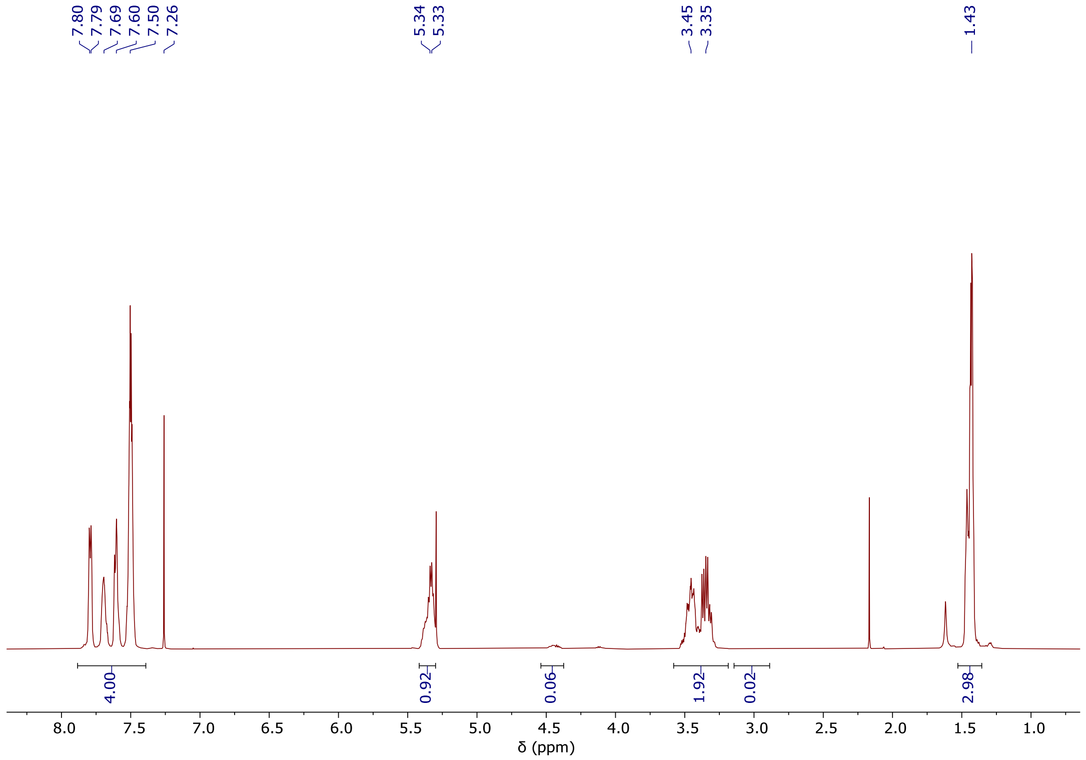

Figure S 2: ^{1}H NMR spectrum (500 MHz, $CDCl_3$) of the isolated PTA/PO copolymer (table S1, run 6).

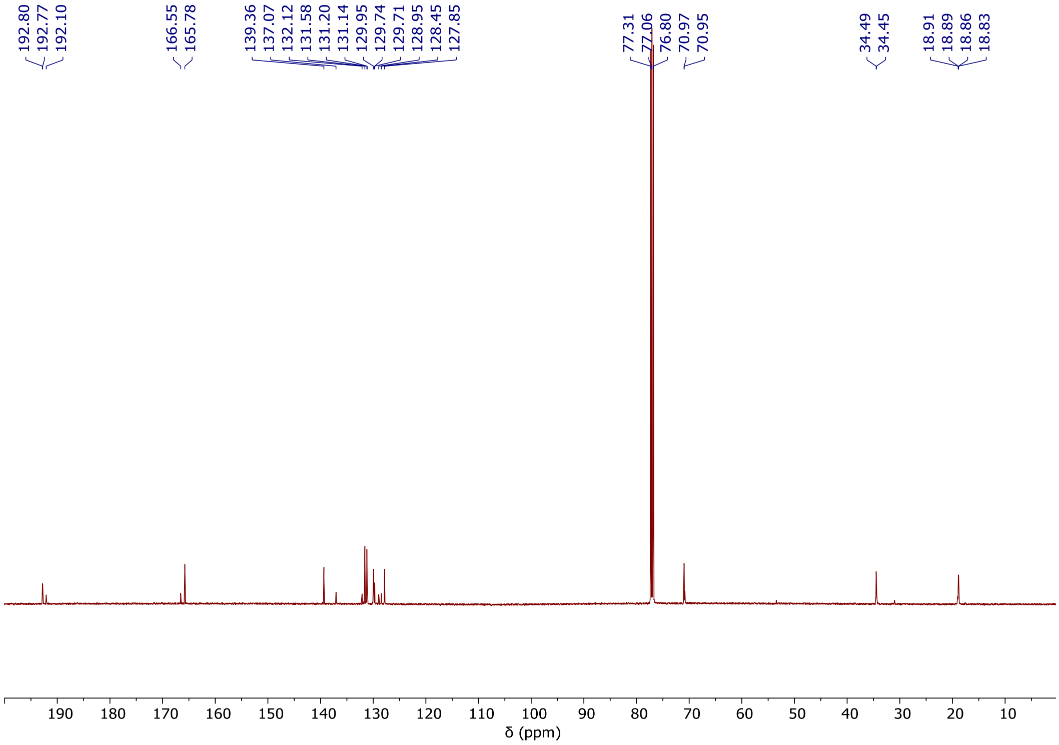

Figure S 3: ^{13}C NMR spectrum (125 MHz, $CDCl_3$) of the isolated PTA/PO copolymer (table S1, run 6). Peak assignments according to literature. [2]

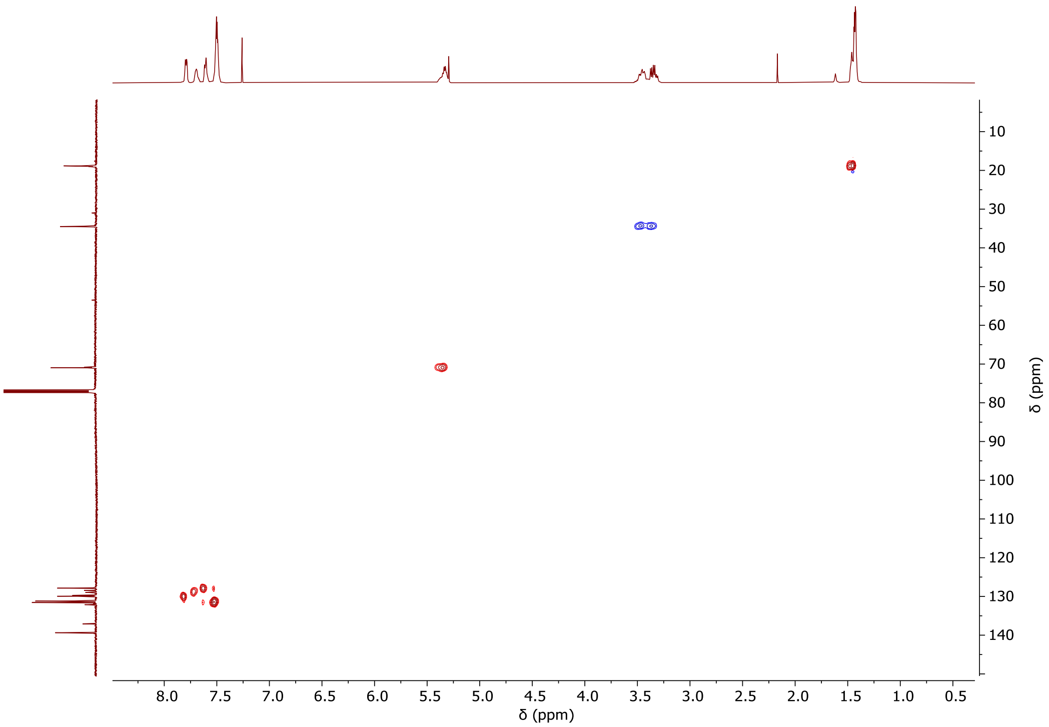

Figure S 4: ^{1}H - ^{13}C HSQC NMR spectrum ($CDCl_3$) of isolated PTA/PO copolymer (table S1, run 6).

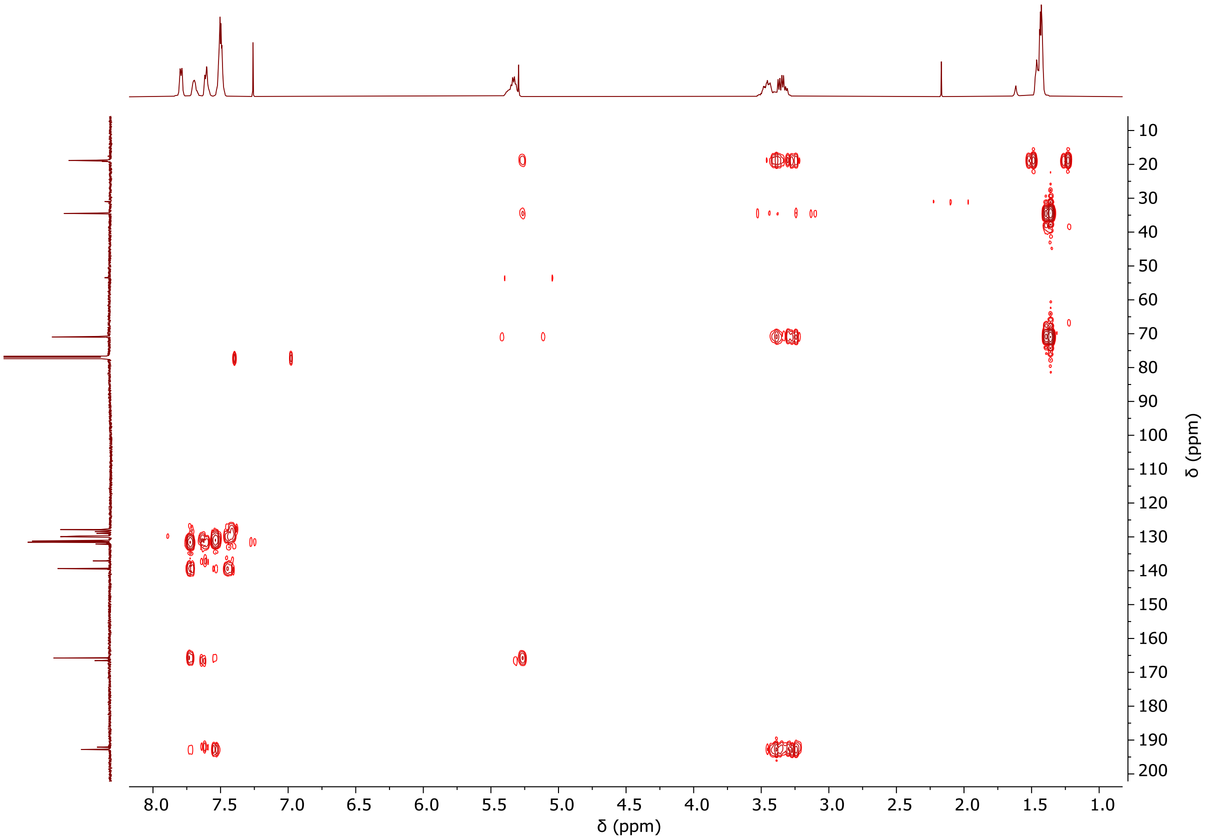

Figure S 5: ^{1}H - ^{13}C HMBC NMR spectrum ($CDCl_3$) of isolated PTA/PO copolymer (table S1, run 6).

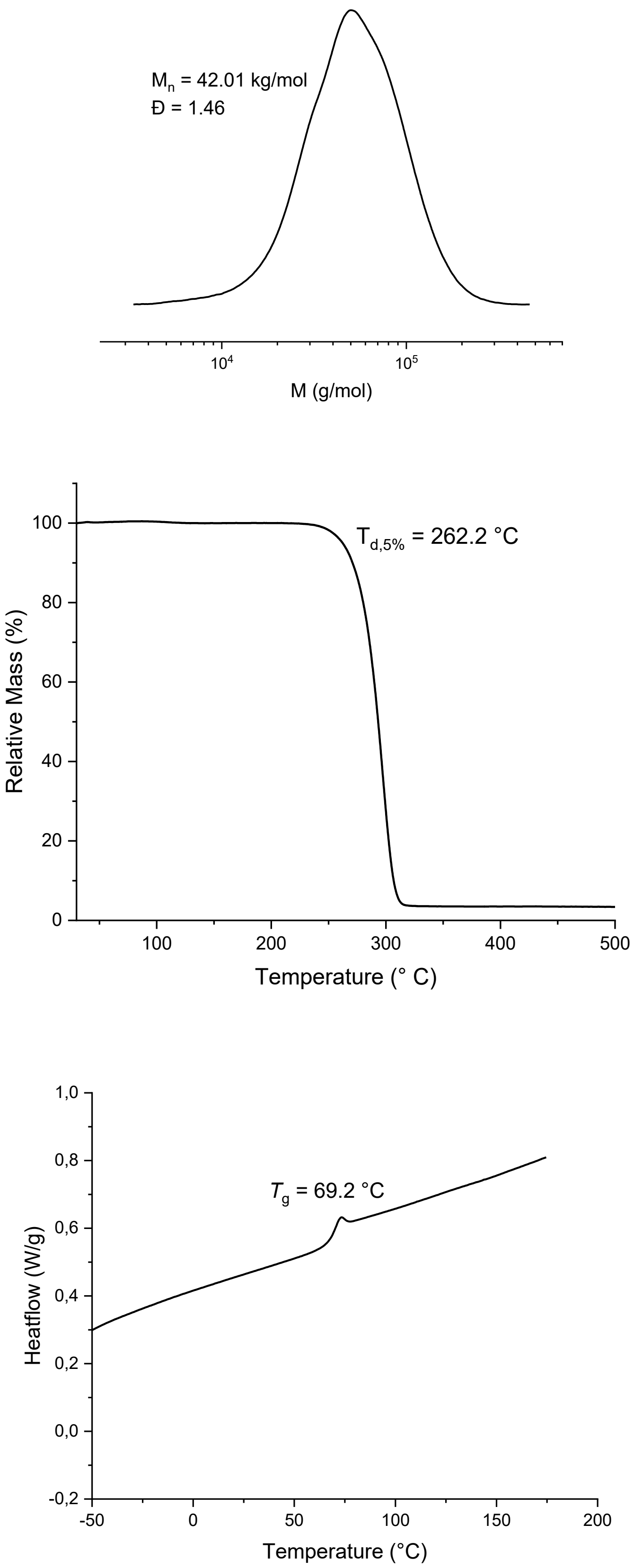

Figure S 6: GPC, TGA and DSC data for the isolated PTA/PO copolymer from table S1, run 6.

b. PTA/CHO ROCOP

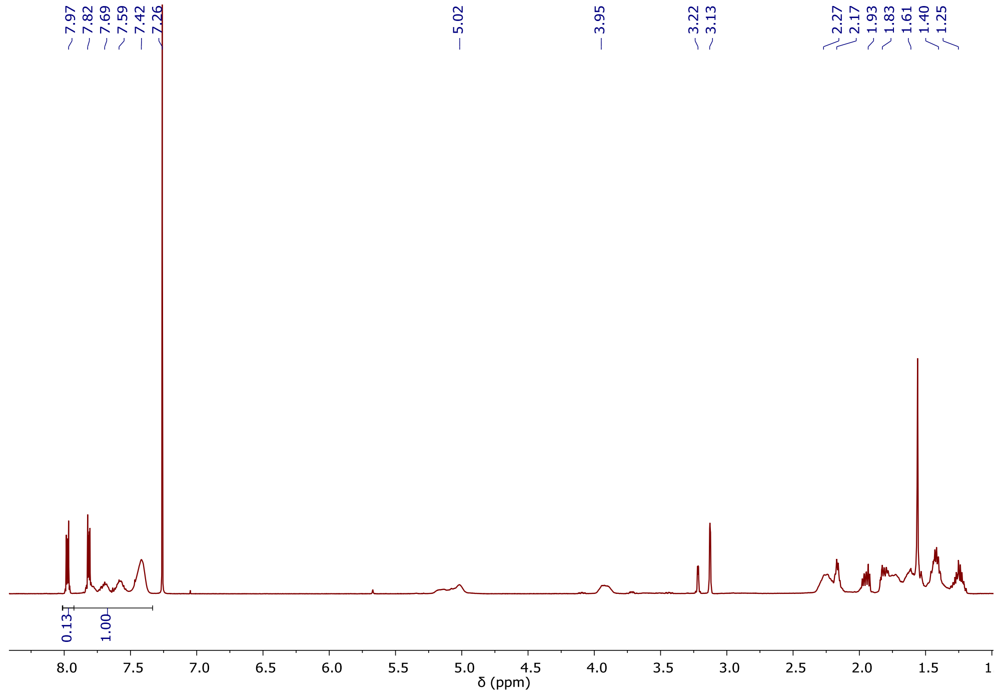

Figure S 7: ^{1}H NMR spectrum (500 MHz, $CDCl_3$) of the final aliquot from PTA/CHO ROCOP (table S2, run 10). Peak assignments according to literature. [1]

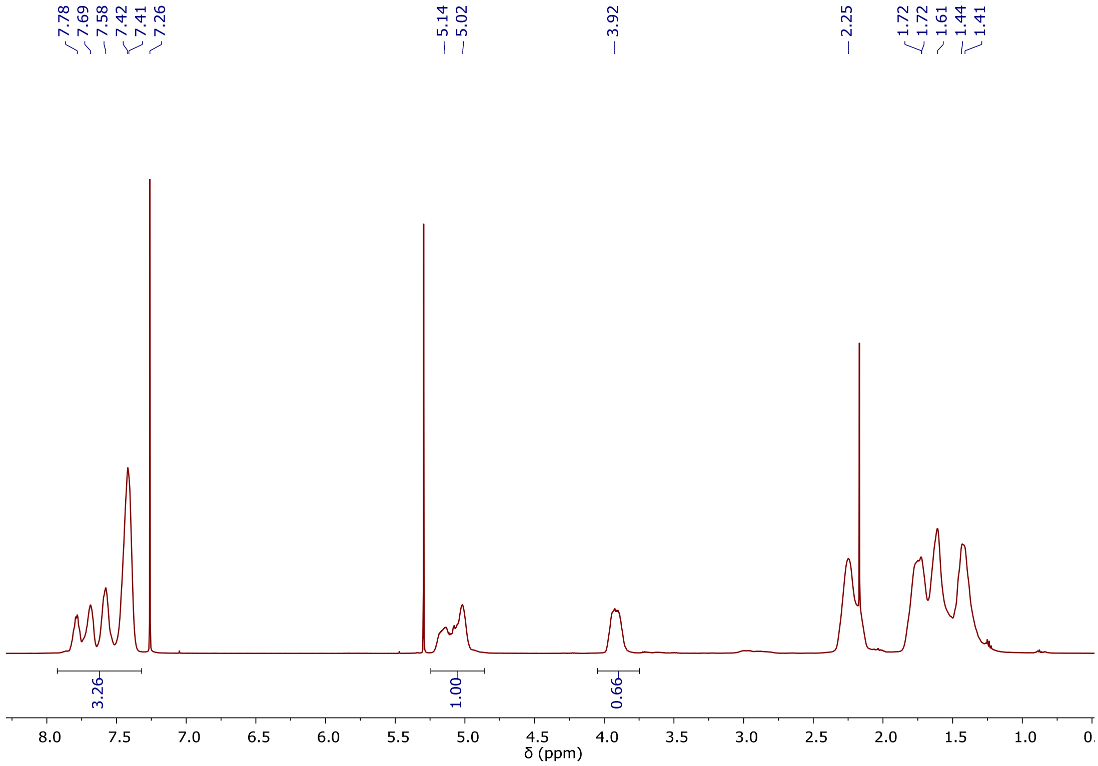

Figure S 8: ^{1}H NMR spectrum (500 MHz, $CDCl_3$) of the isolated PTA/CHO copolymer (table S2, run 10).

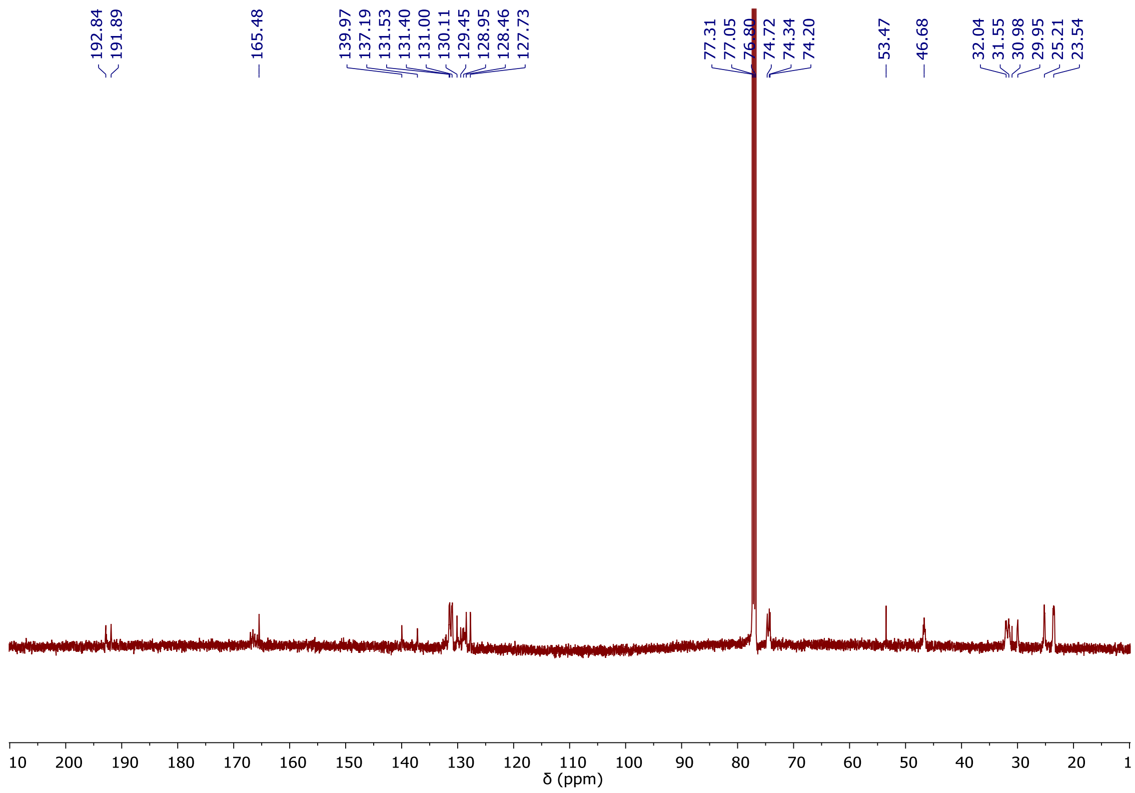

Figure S 9: ^{13}C NMR spectrum (125 MHz, $CDCl_3$) of the isolated PTA/CHO copolymer (table S2, run 10). Peak assignments according to literature. [1]

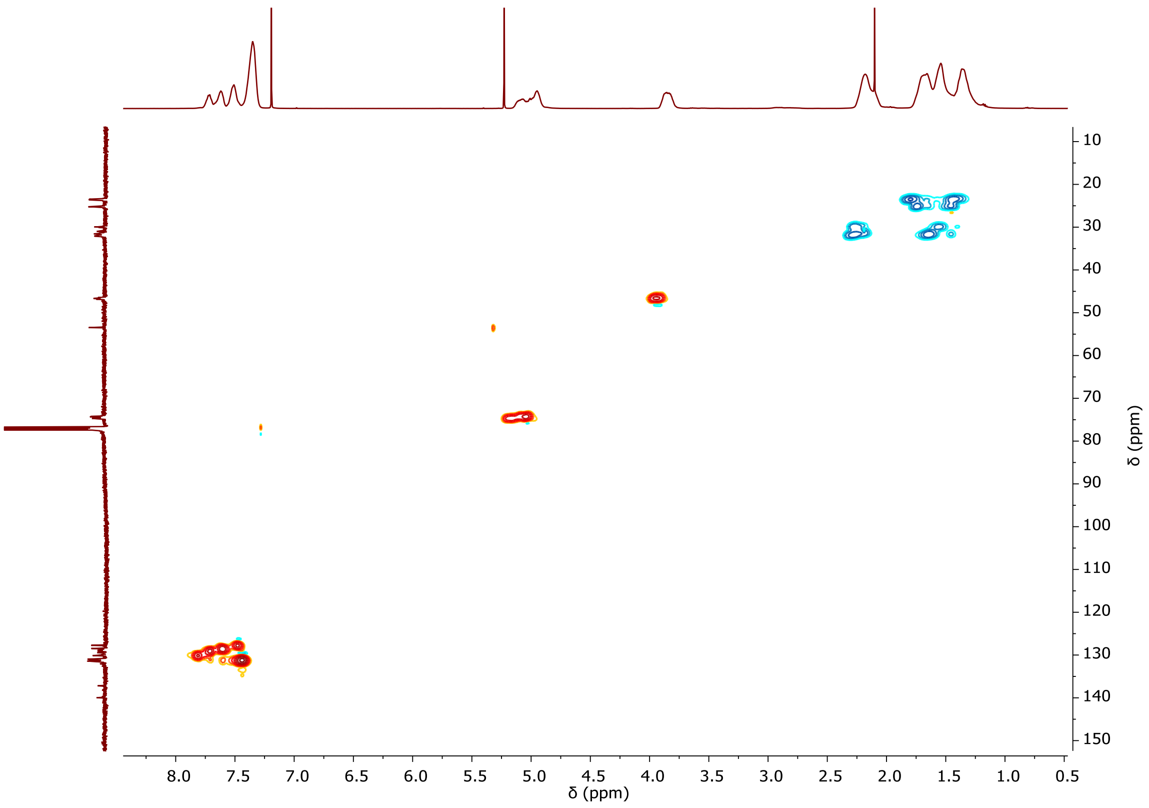

Figure S 10: ^{1}H - ^{13}C HSQC NMR spectrum ($CDCl_3$) of isolated PTA/CHO copolymer (table S2, run 10).

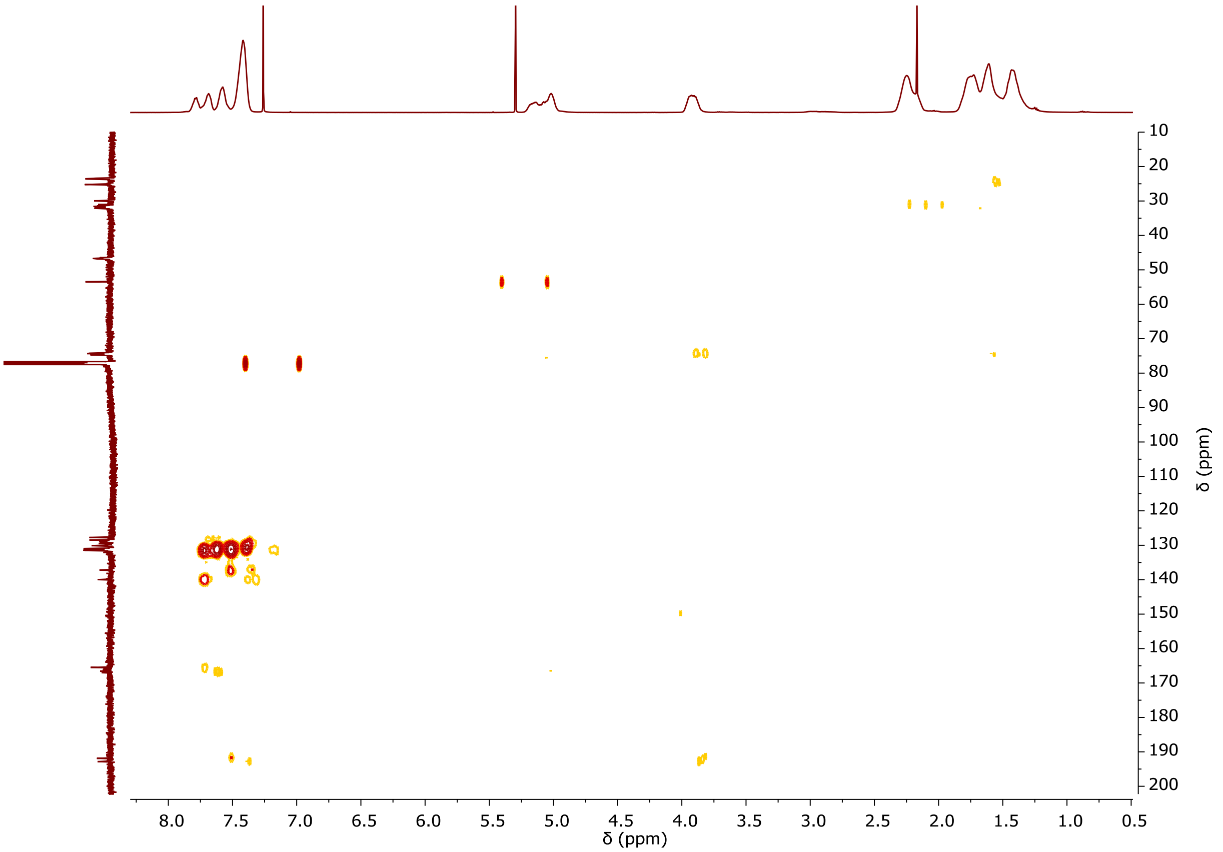

Figure S 11: ^{1}H - ^{13}C HMBC NMR spectrum ($CDCl_3$) of isolated PTA/CHO copolymer (table S2, run 10).

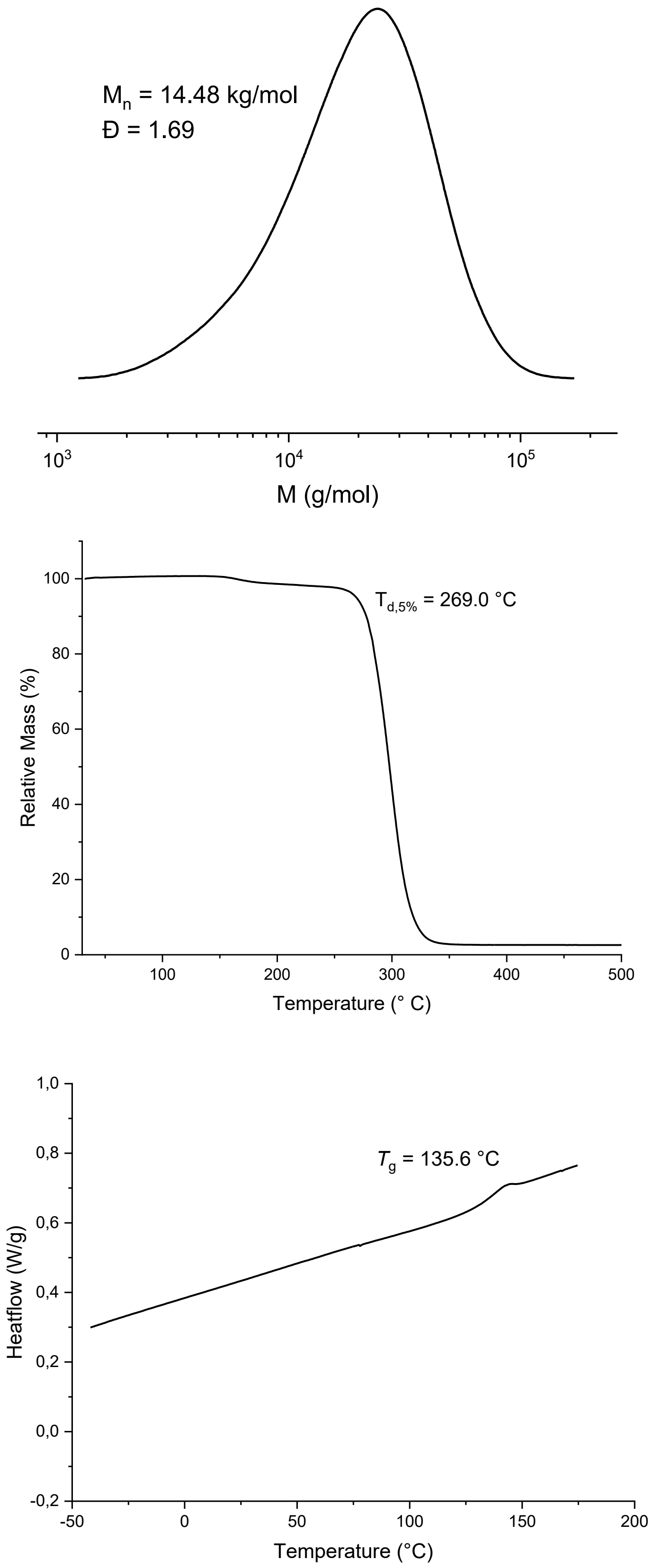

Figure S 12: GPC, TGA and DSC data for the isolated PTA/CHO copolymer from table S2, run 10.

c. PTA/PGE ROCOP

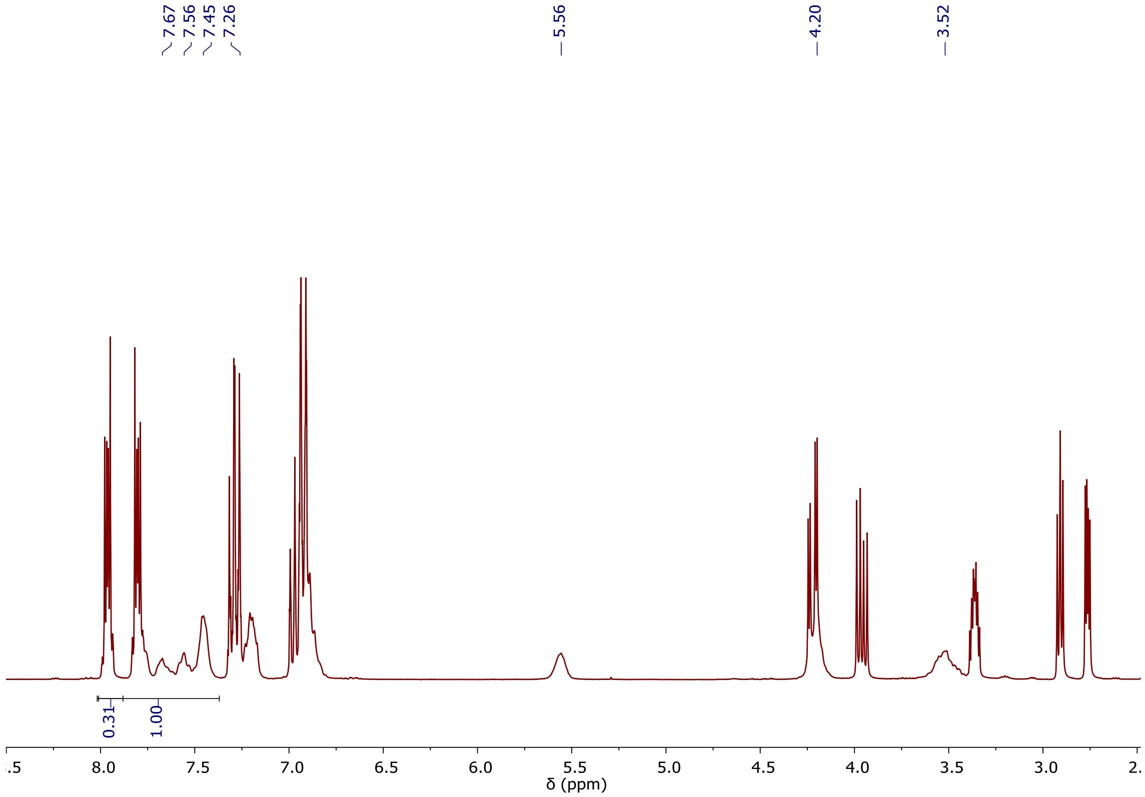

Figure S 13: ^{1}H NMR spectrum (300 MHz, $CDCl_3$) of the final aliquot from PTA/PGE ROCOP (table 2, run 12). Peak assignments according to Figure S 14.

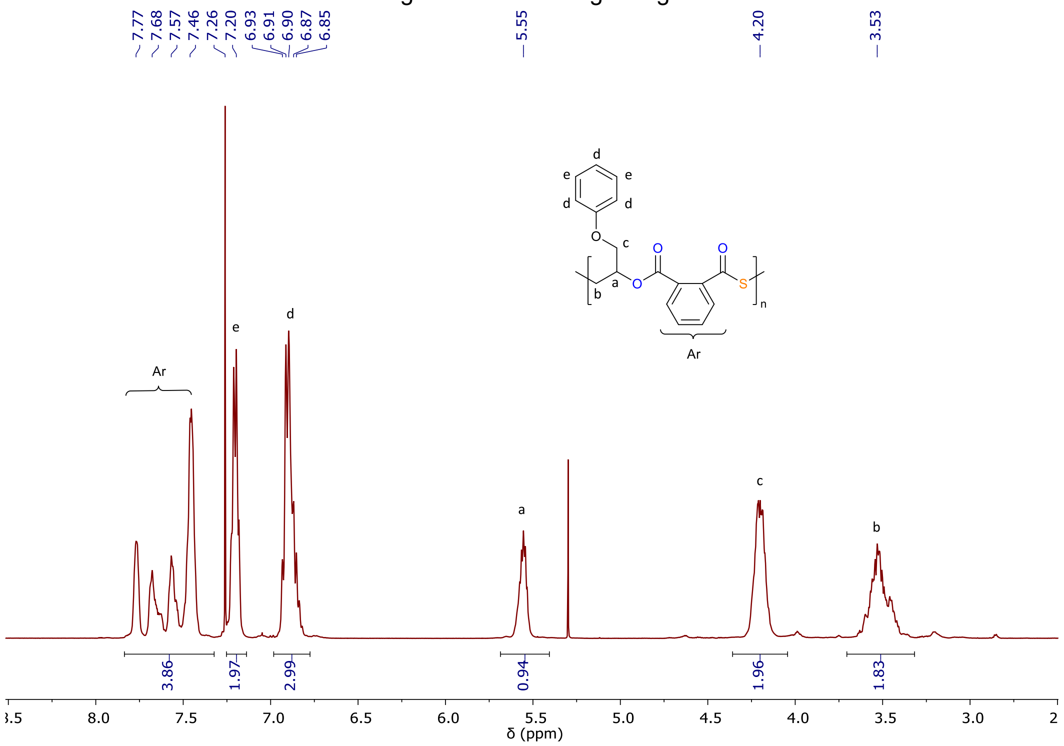

Figure S 14: ^{1}H NMR spectrum (500 MHz, $CDCl_3$) of the isolated PTA/PGE copolymer (table 2, run 12).

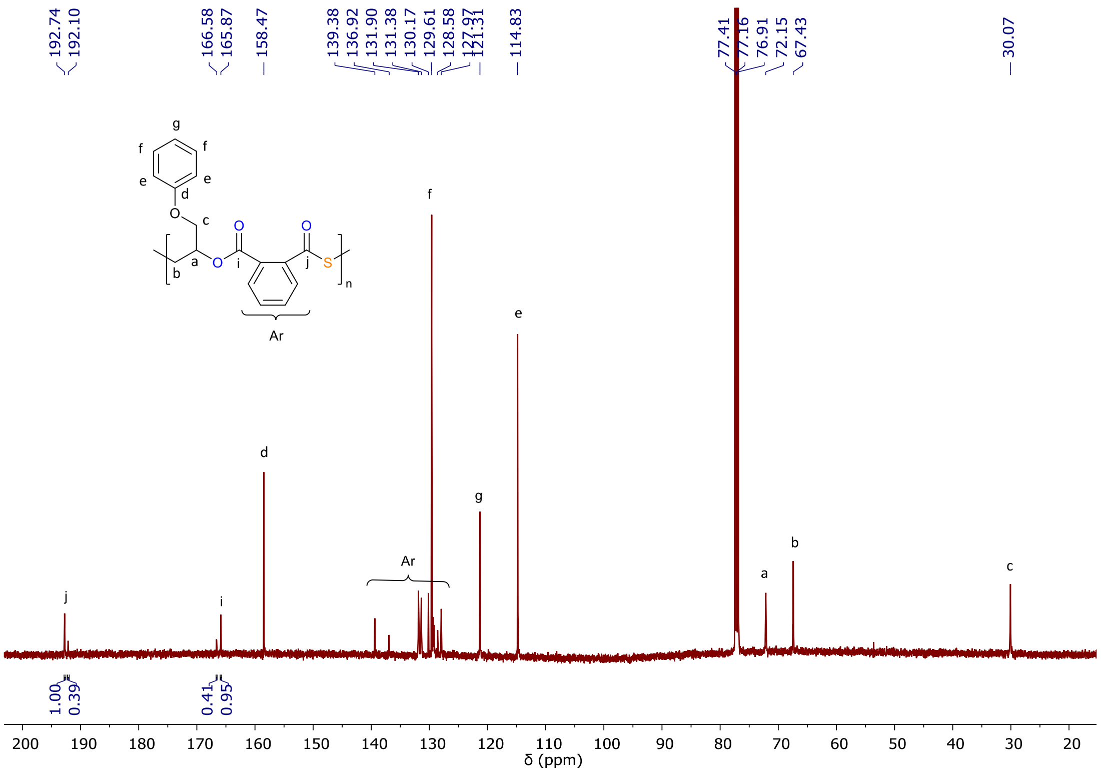

Figure S 15: ^{13}C NMR spectrum (125 MHz, $CDCl_3$) of the isolated PTA/PGE copolymer (table 2, run 12)

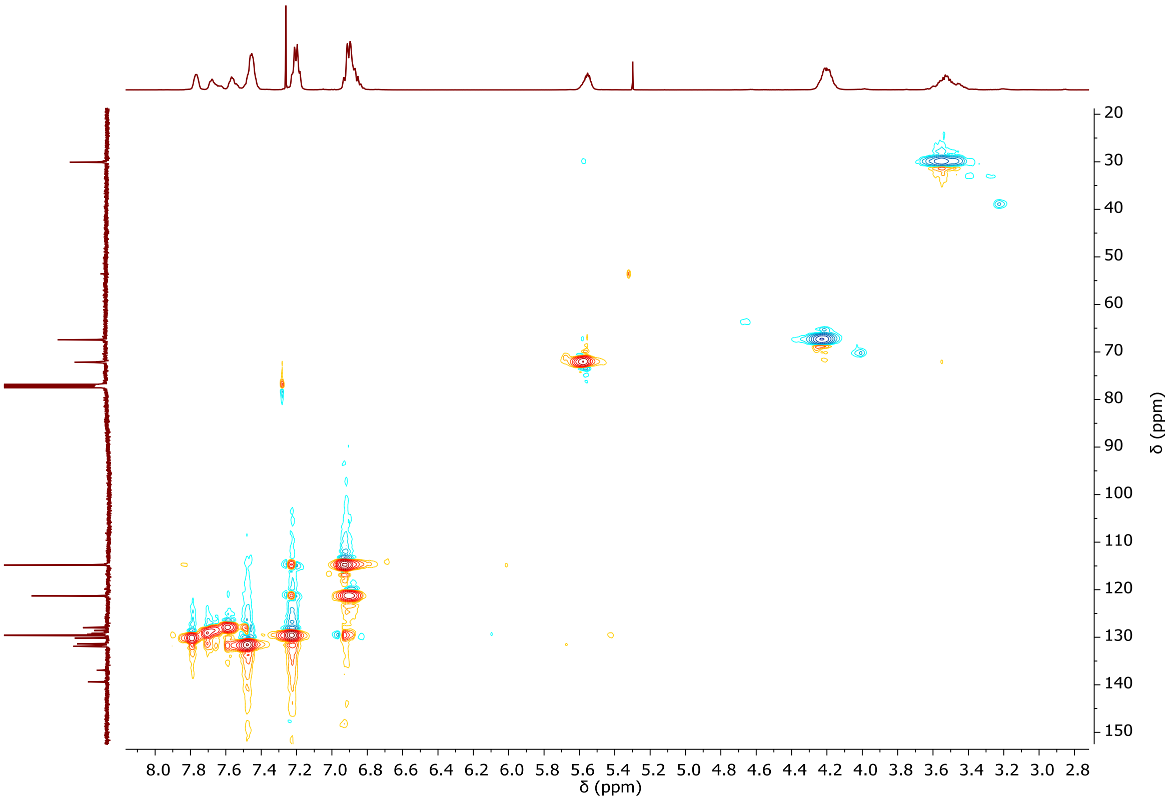

Figure S 16: ^{1}H - ^{13}C HSQC NMR spectrum ($CDCl_3$) of isolated PTA/PGE copolymer (table 2, run 12).

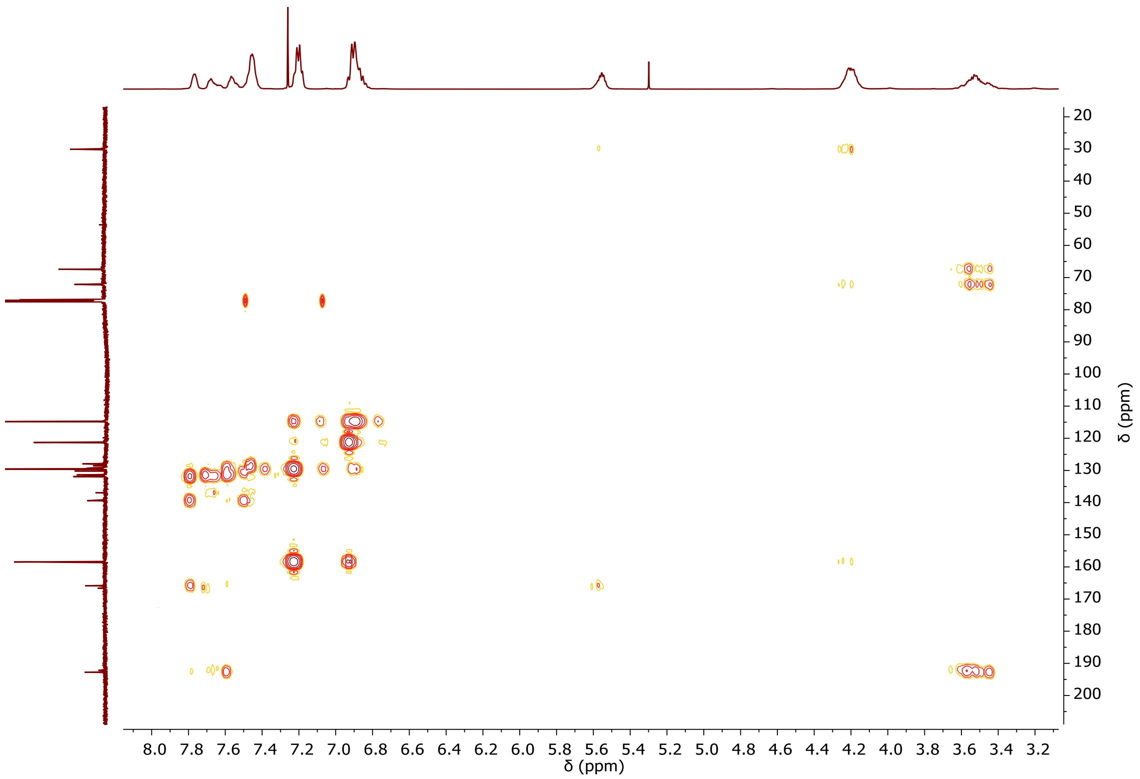

Figure S 17: ^{1}H - ^{13}C HMBC NMR spectrum ($CDCl_3$) of isolated PTA/PGE copolymer (table 2, run 12).

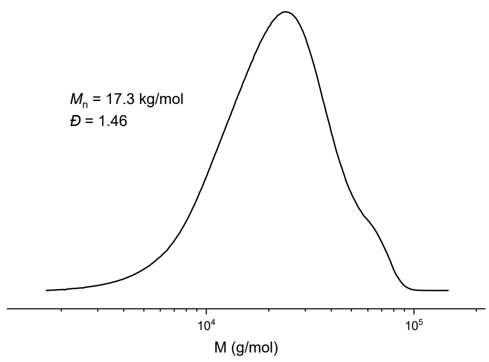

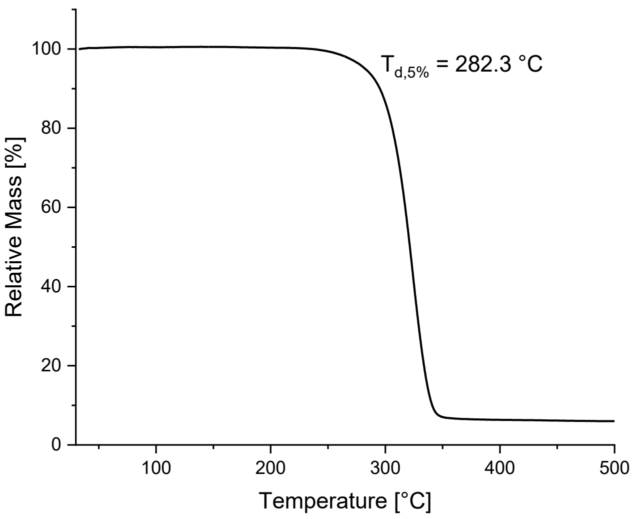

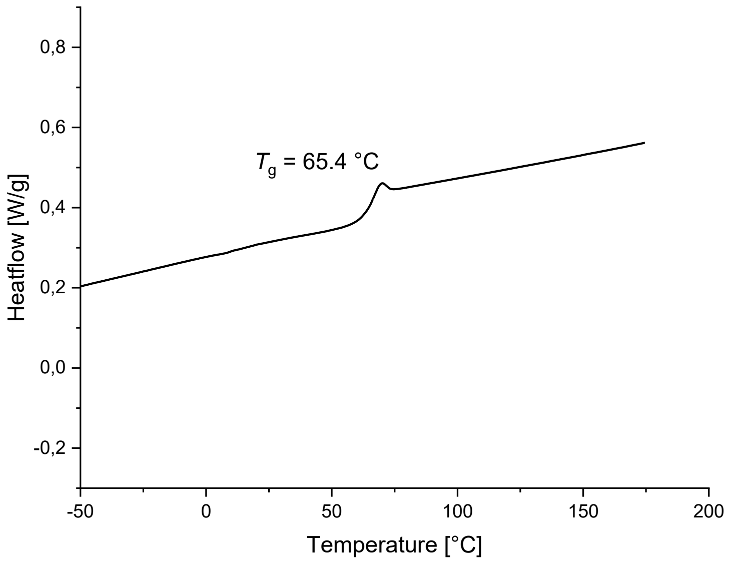

Figure S 18: GPC, TGA and DSC data for the isolated PTA/PGE copolymer from table 2, run 12.

d. CS_2/PO ROCOP

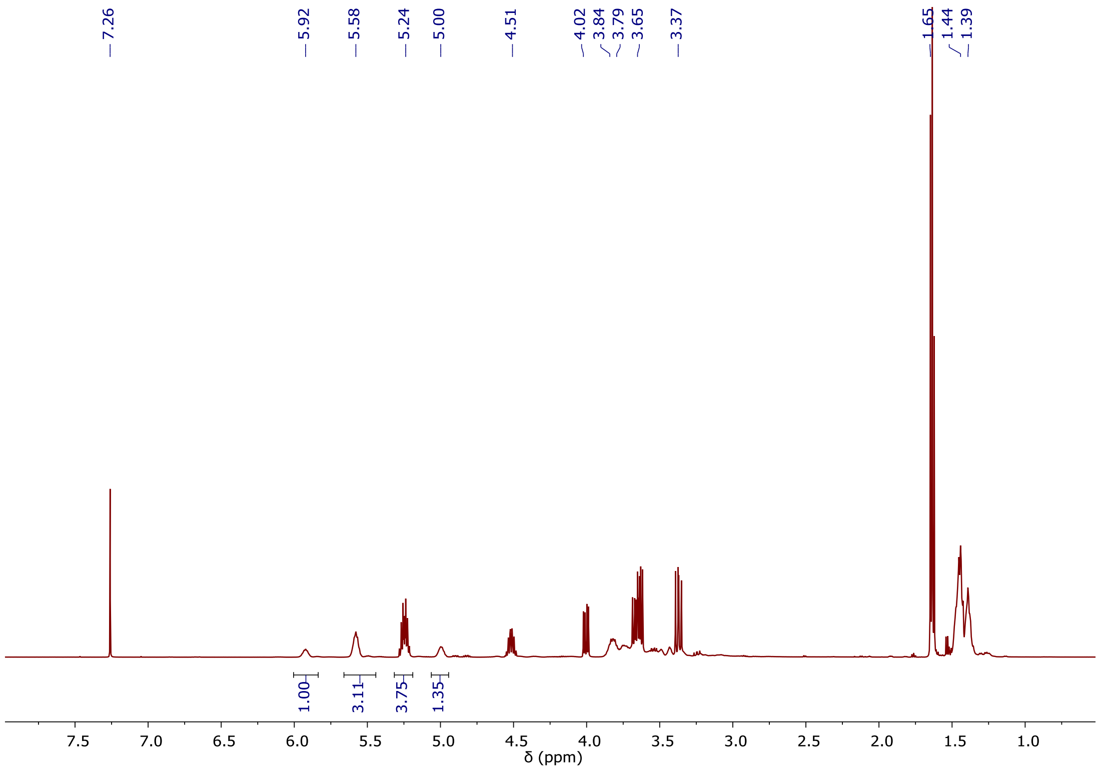

Figure S 19: ^{1}H NMR spectrum (500 MHz, $CDCl_3$) of the final aliquot from CS_2/PO ROCOP (table S3, run 6). Peak assignments according to literature. [3]

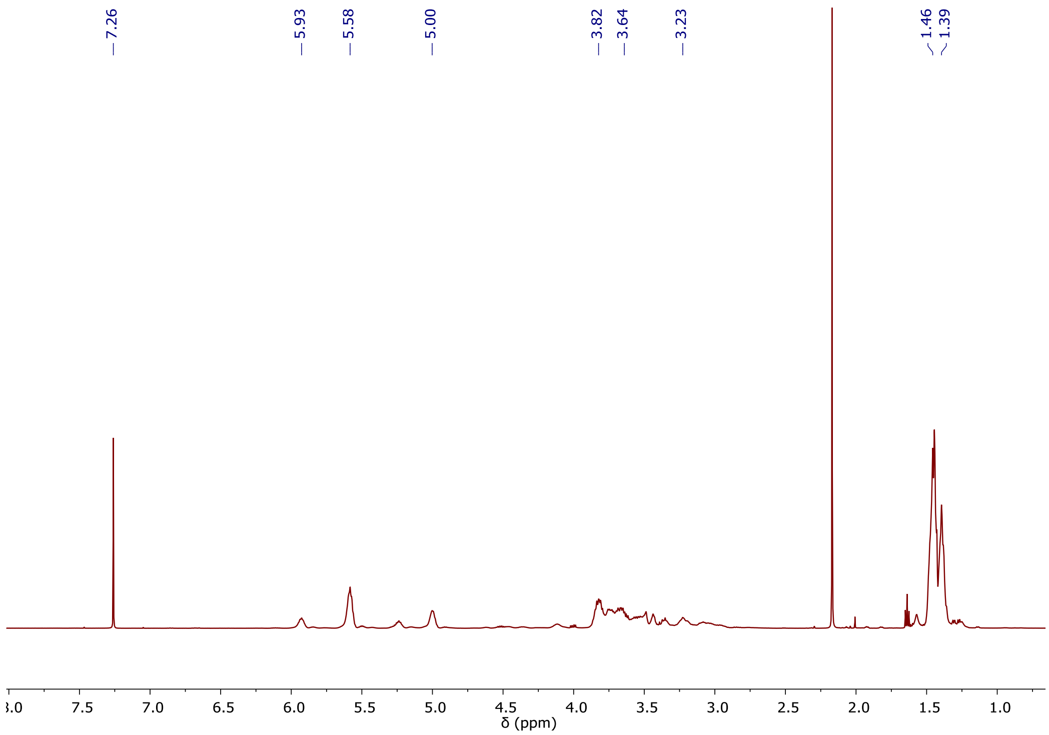

Figure S 20: ^{1}H NMR spectrum (500 MHz, $CDCl_3$) of the isolated CS_2/PO copolymer (table S3, run 6).

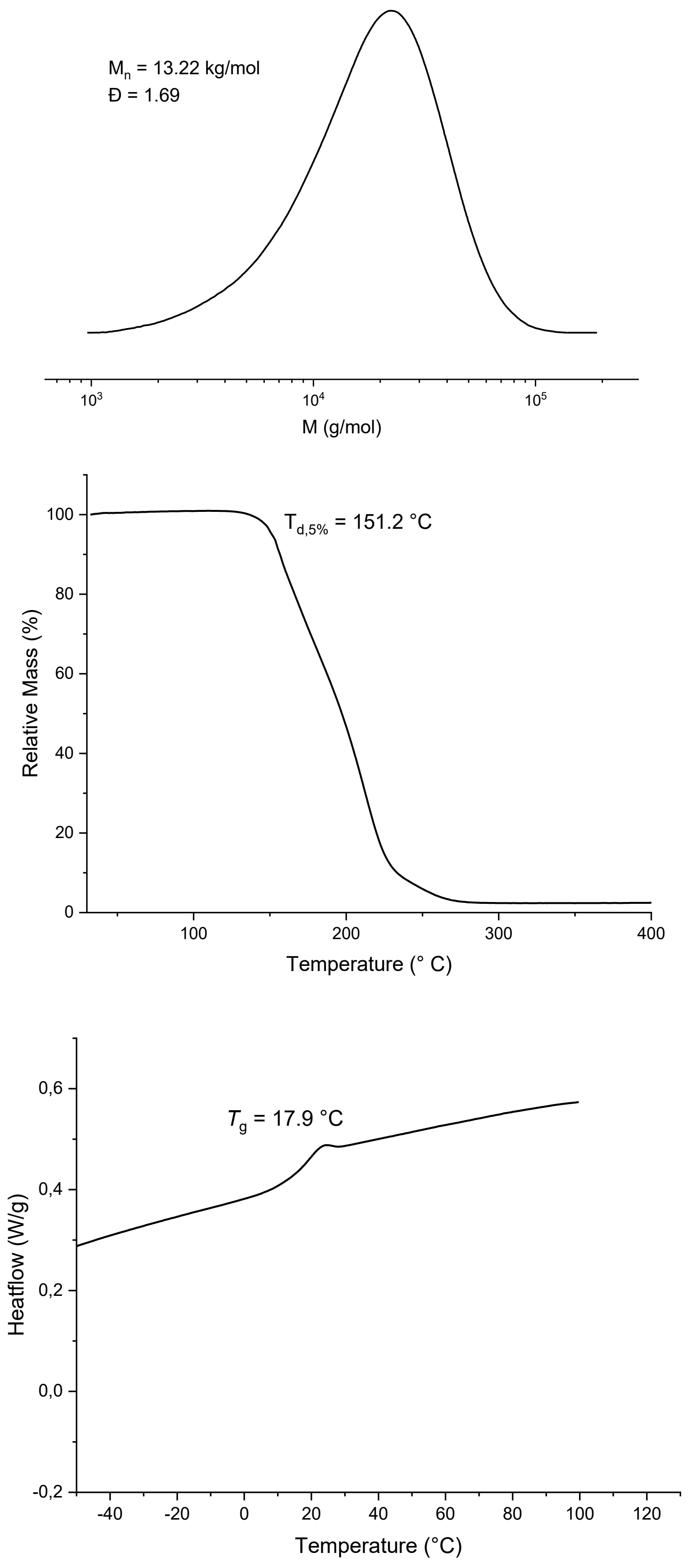

Figure S 21: GPC, TGA and DSC data for the isolated CS_2/PO copolymer from table S3, run 6.

e. CS_2/CHO ROCOP

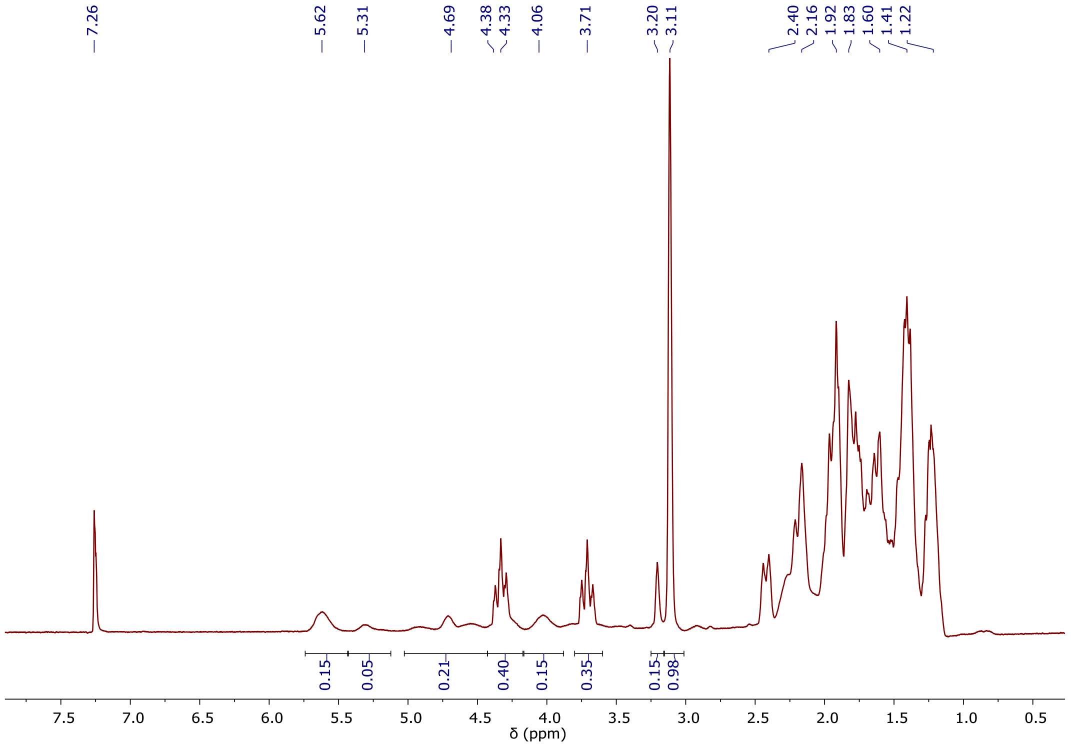

Figure S 22: ^{1}H NMR spectrum (500 MHz, $CDCl_3$) of the final aliquot from CS_2/CHO ROCOP (table S4, run 6). Peak assignments according to literature. [4]

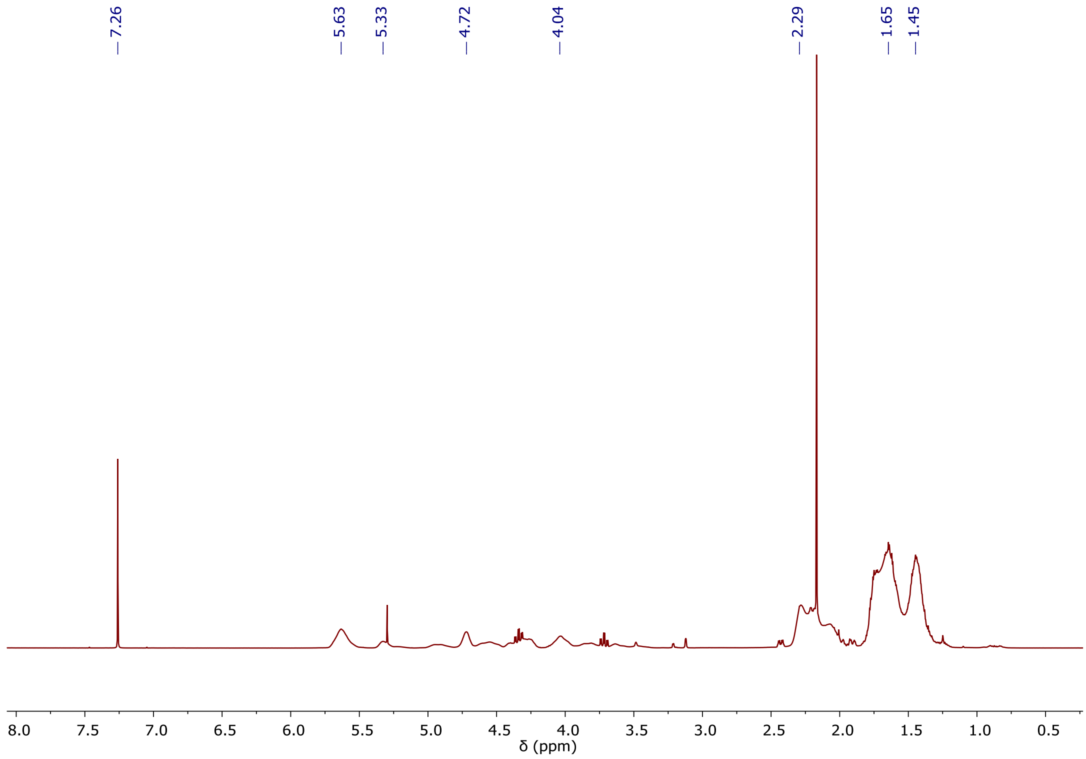

Figure S 23: ^{1}H NMR spectrum (500 MHz, $CDCl_3$) of the isolated CS_2/CHO copolymer (table S4, run 6).

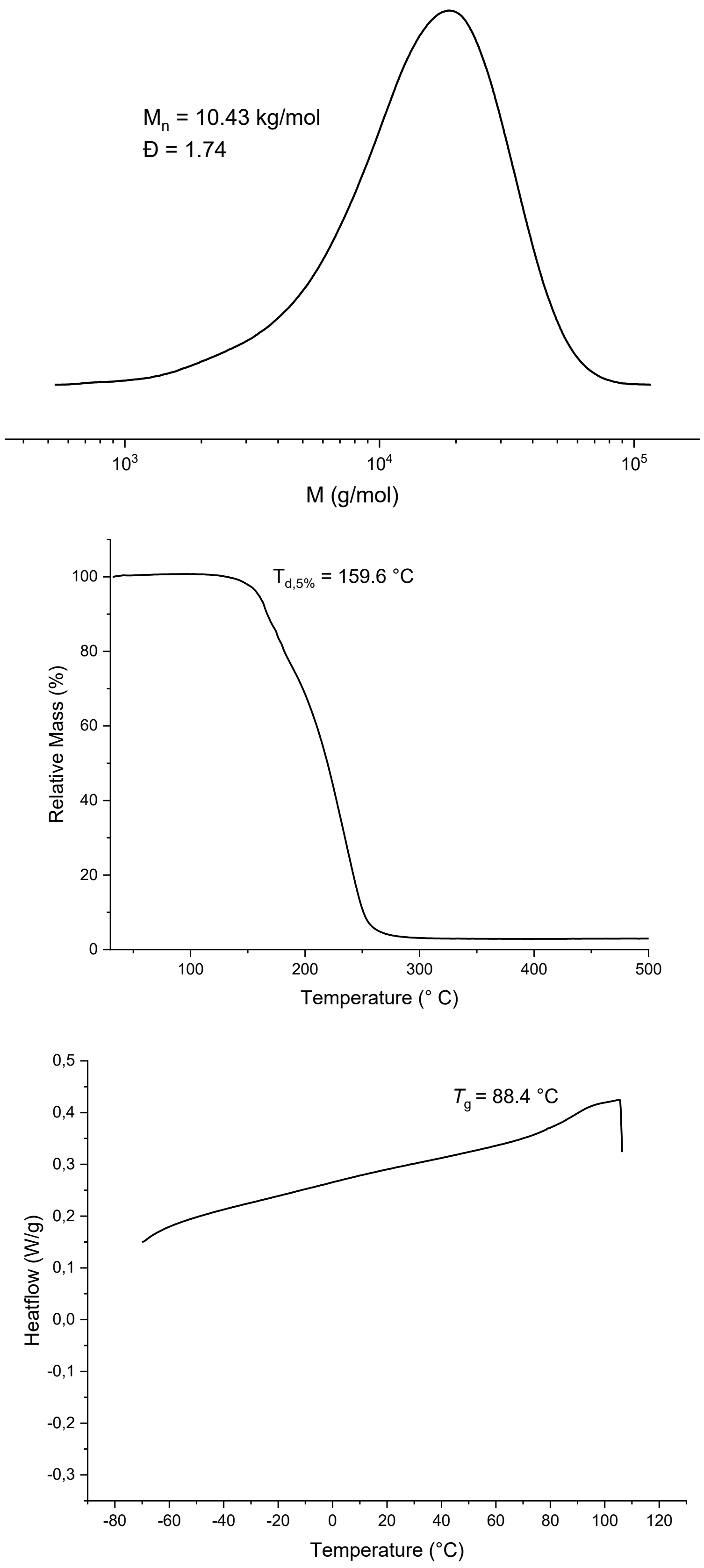

Figure S 24: GPC, TGA and DSC data for the isolated CS_2/CHO copolymer from table S4, run 6.

f. CS_2/PGE ROCOP

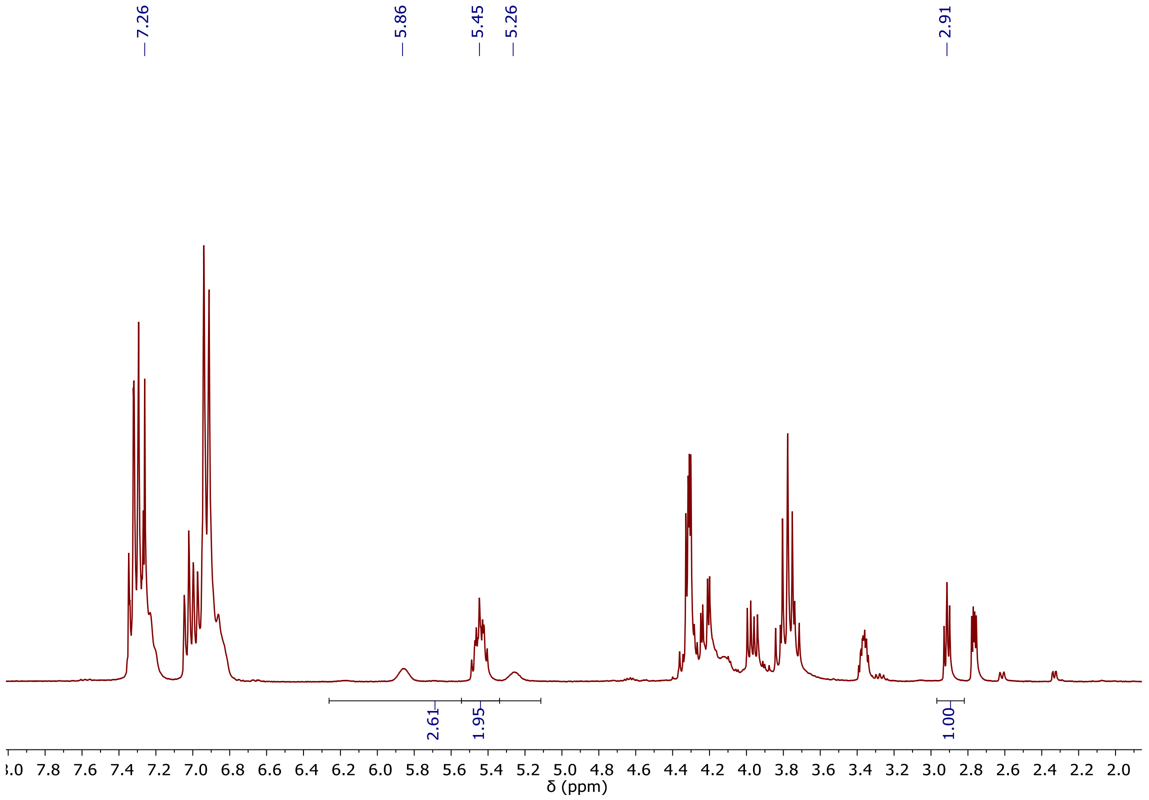

Figure S 25: ^{1}H NMR spectrum (300 MHz, $CDCl_3$) of the final aliquot from CS_2/PGE ROCOP (table 2, run 10).

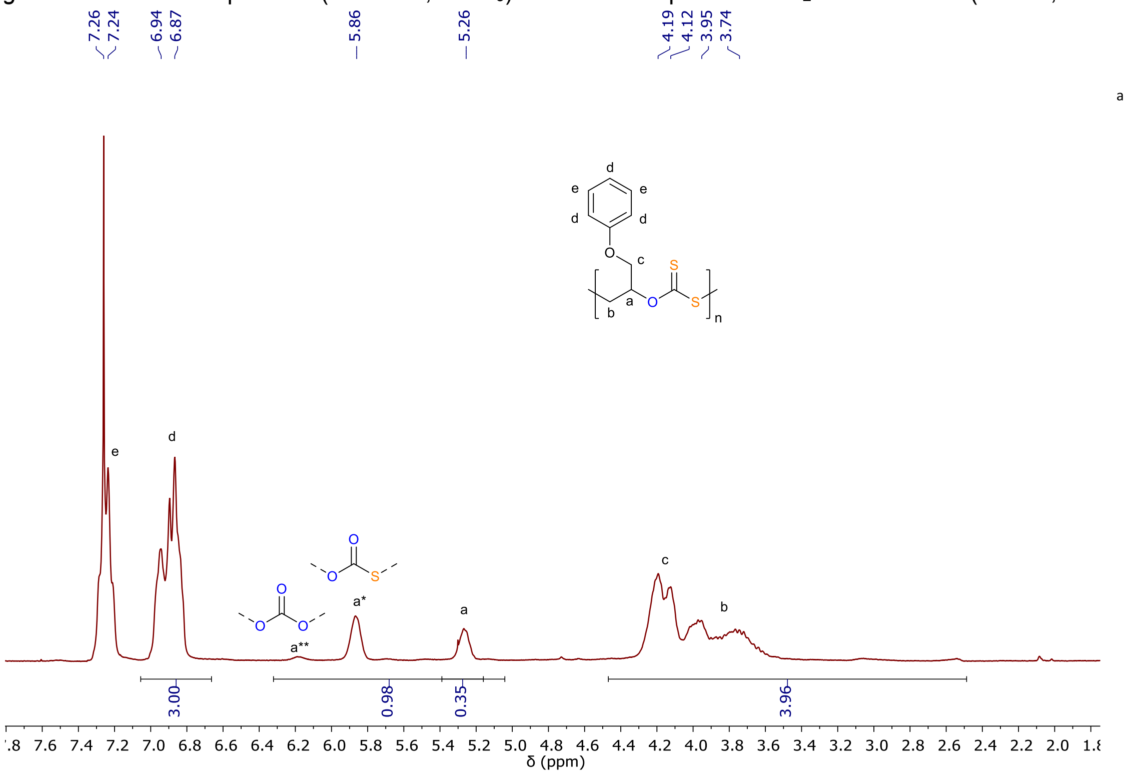

Figure S 26: ^{1}H NMR spectrum (300 MHz, $CDCl_3$) of the isolated CS_2/PGE copolymer (table 2, run 10).

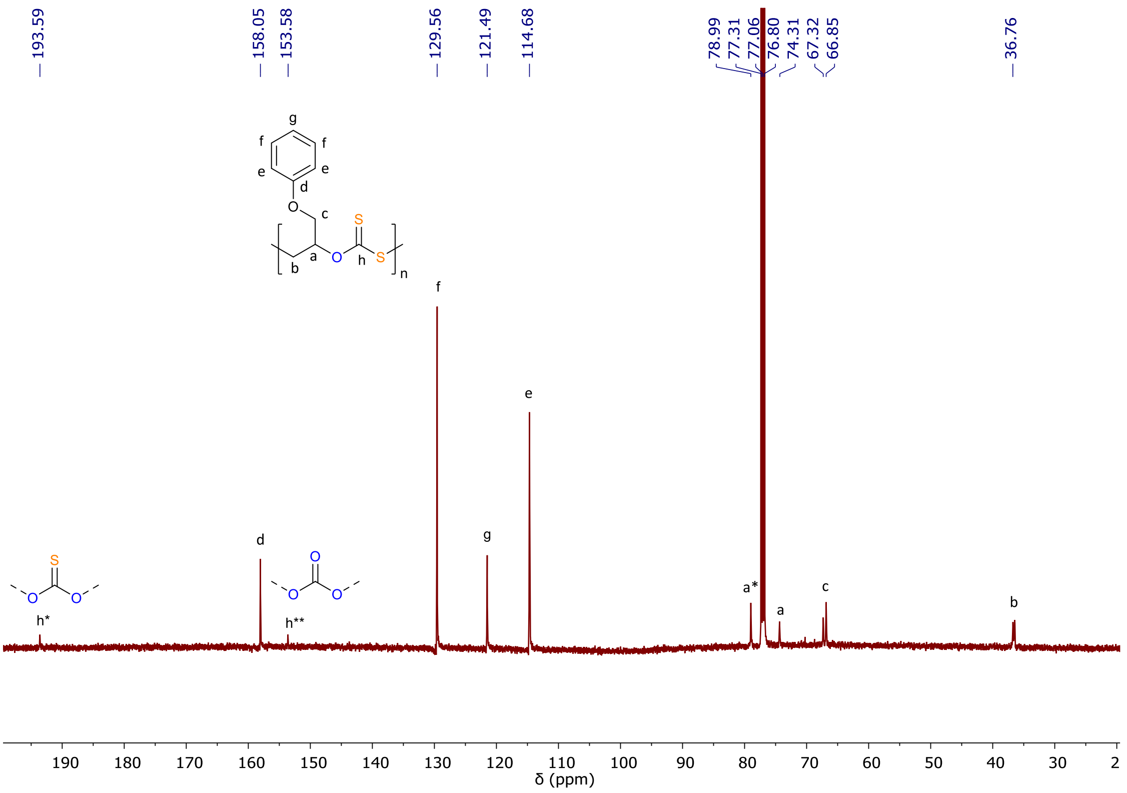

Figure S 27: ^{13}C NMR spectrum (125 MHz, $CDCl_3$) of the isolated CS_2/PGE copolymer (table 2, run 10)

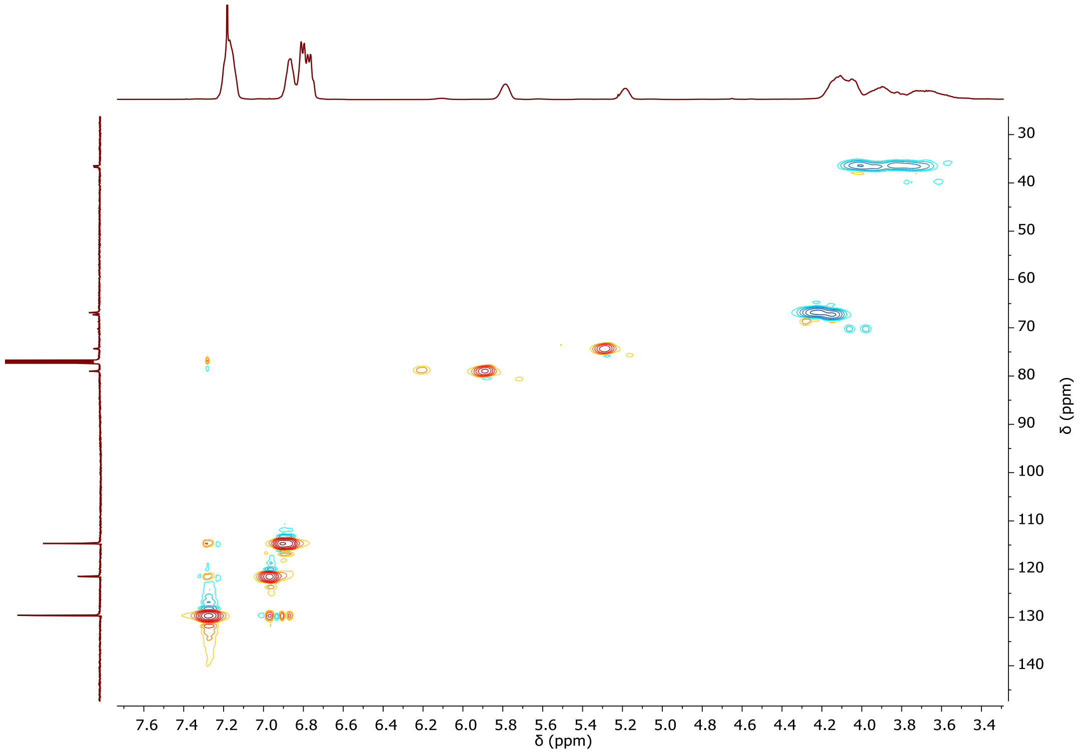

Figure S 28: ^{1}H - ^{13}C HSQC NMR spectrum ($CDCl_3$) of isolated CS_2/PGE copolymer (table 2, run 10).

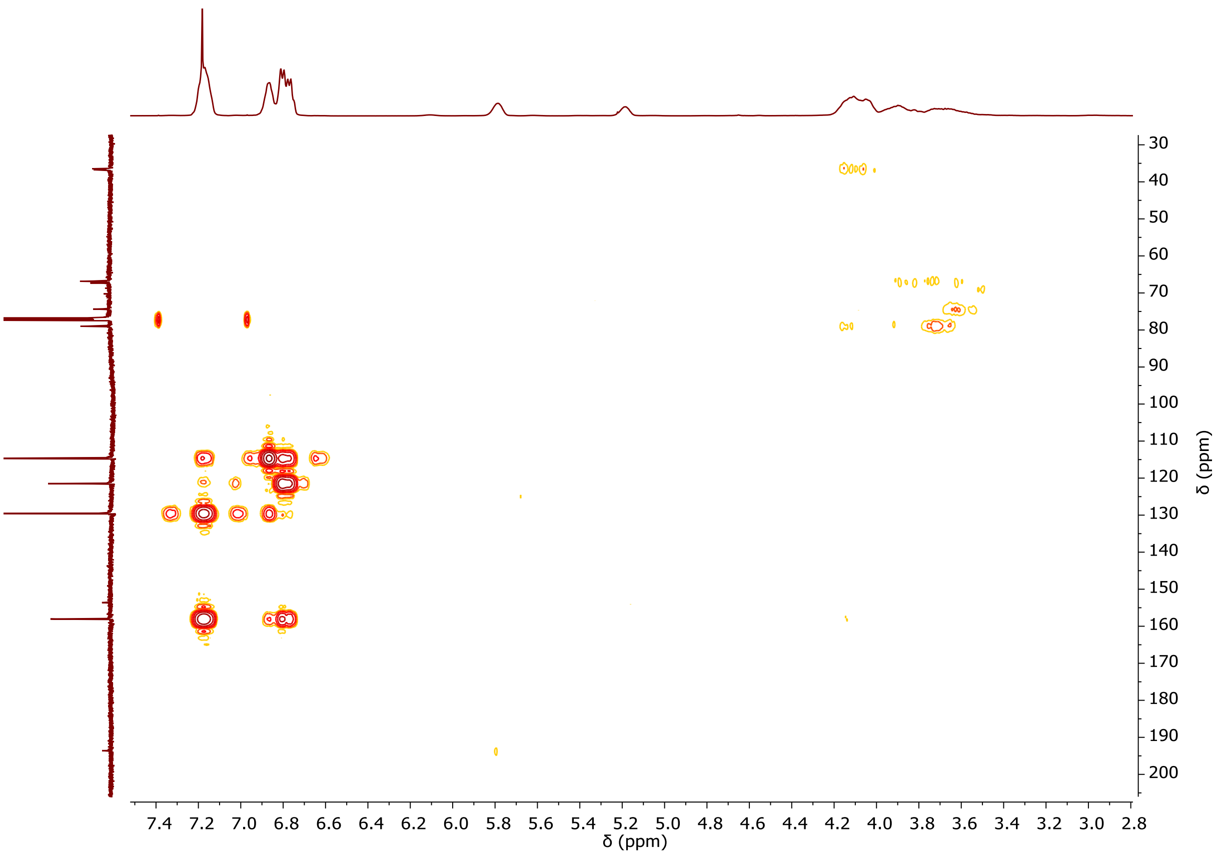

Figure S 29: ^{1}H - ^{13}C HMBC NMR spectrum ($CDCl_3$) of isolated CS_2/PGE copolymer (table 2, run 10).

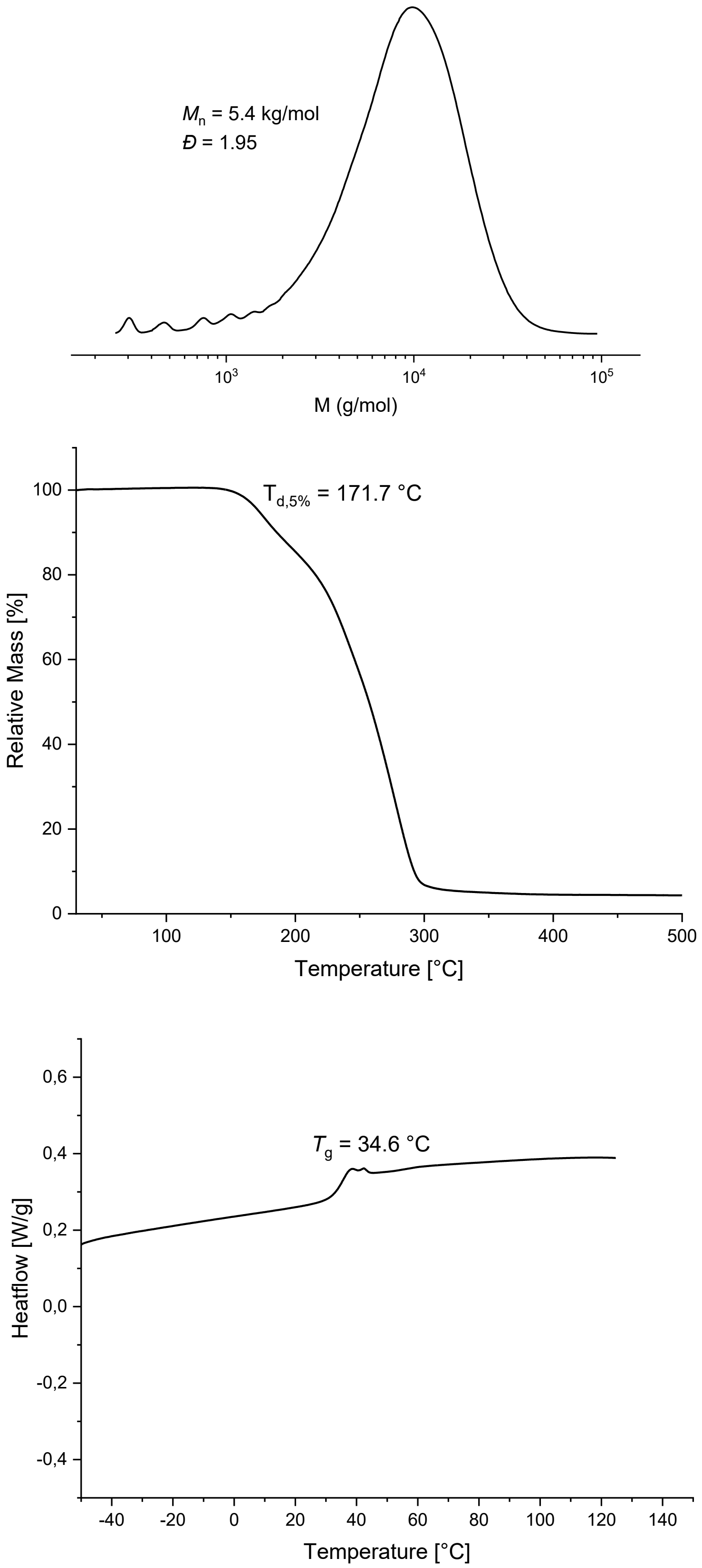

Figure S 30: GPC, TGA and DSC data for the isolated CS_2/PGE copolymer from table 2, run 10.

g. PhNCS/epoxide ROCOP

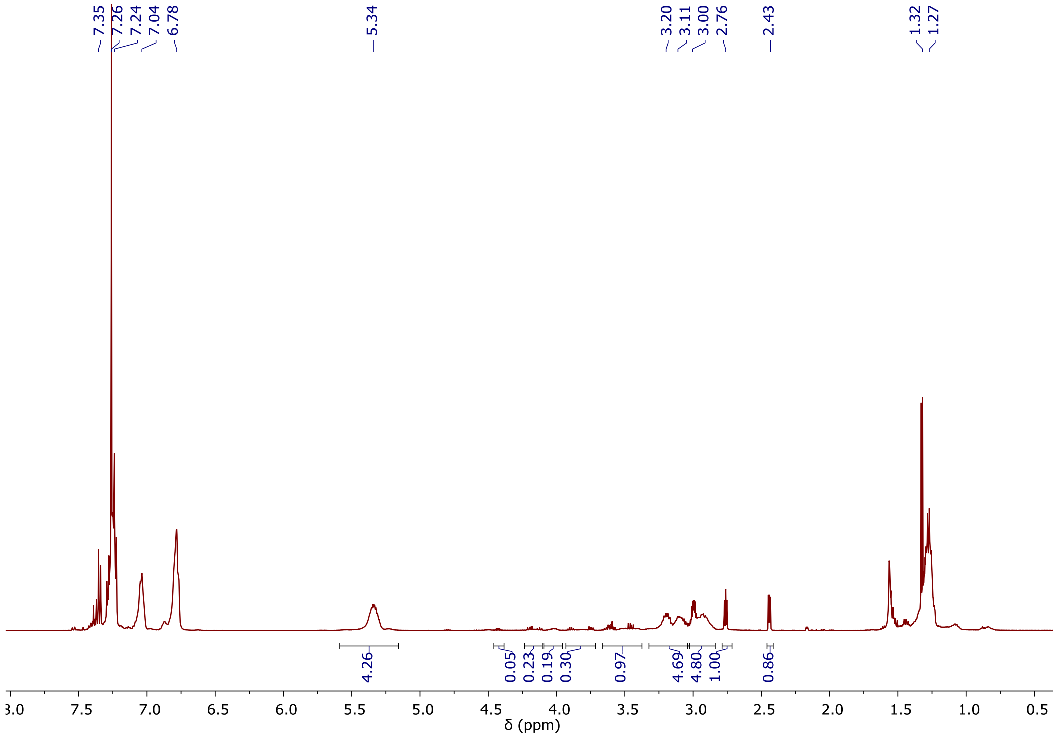

Figure S 31: ^{1}H NMR spectrum (500 MHz, $CDCl_3$) of the final aliquot from PhNCS/PO ROCOP (table S7, run 1).

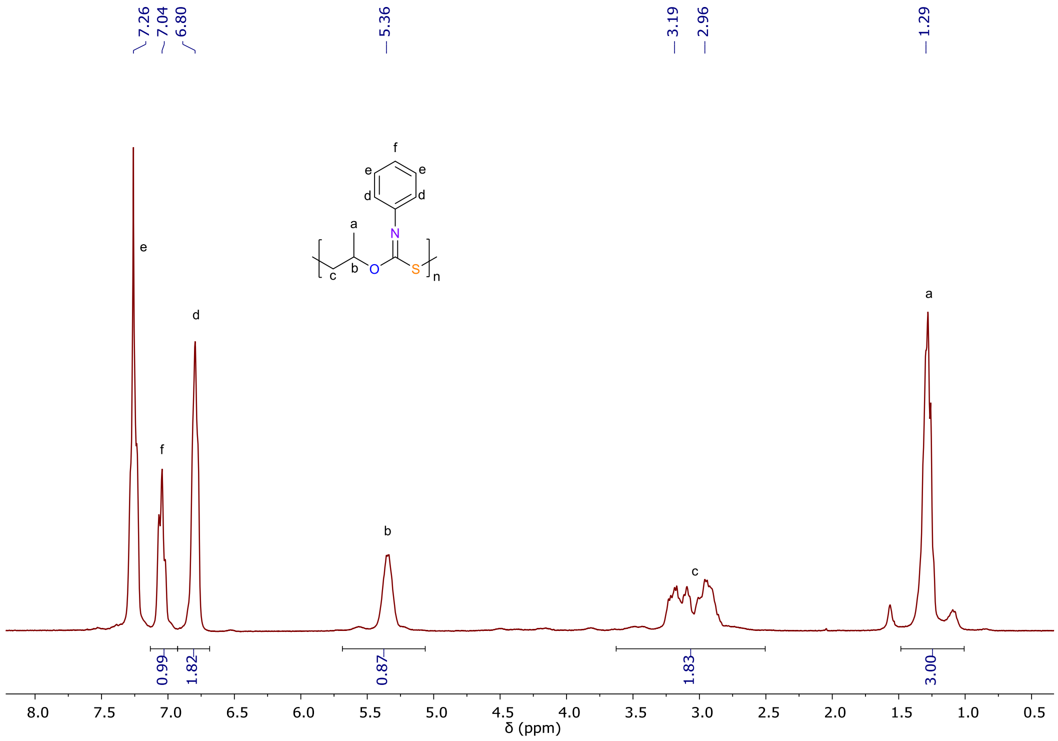

Figure S 32: ^{1}H NMR spectrum (500 MHz, $CDCl_3$) of the isolated PhNCS/PO copolymer (table S7, run 1).

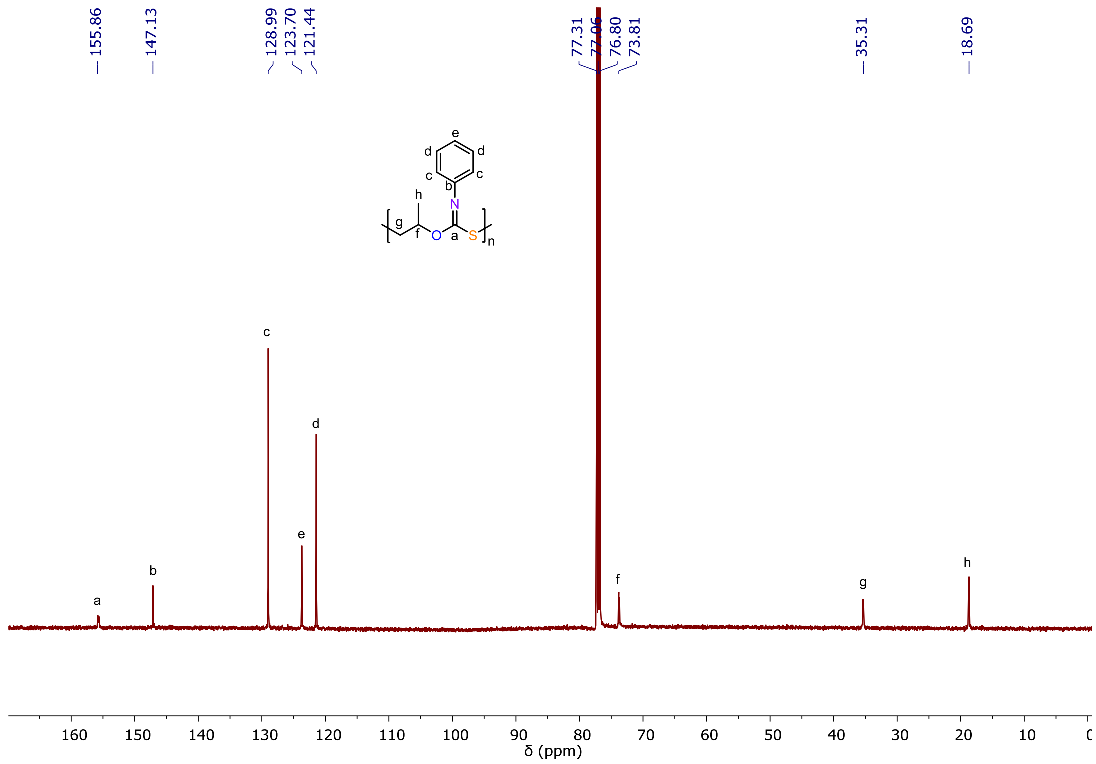

Figure S 33: ^{13}C NMR spectrum (125 MHz, $CDCl_3$) of the isolated PhNCS/PO copolymer (table S7, run 1).

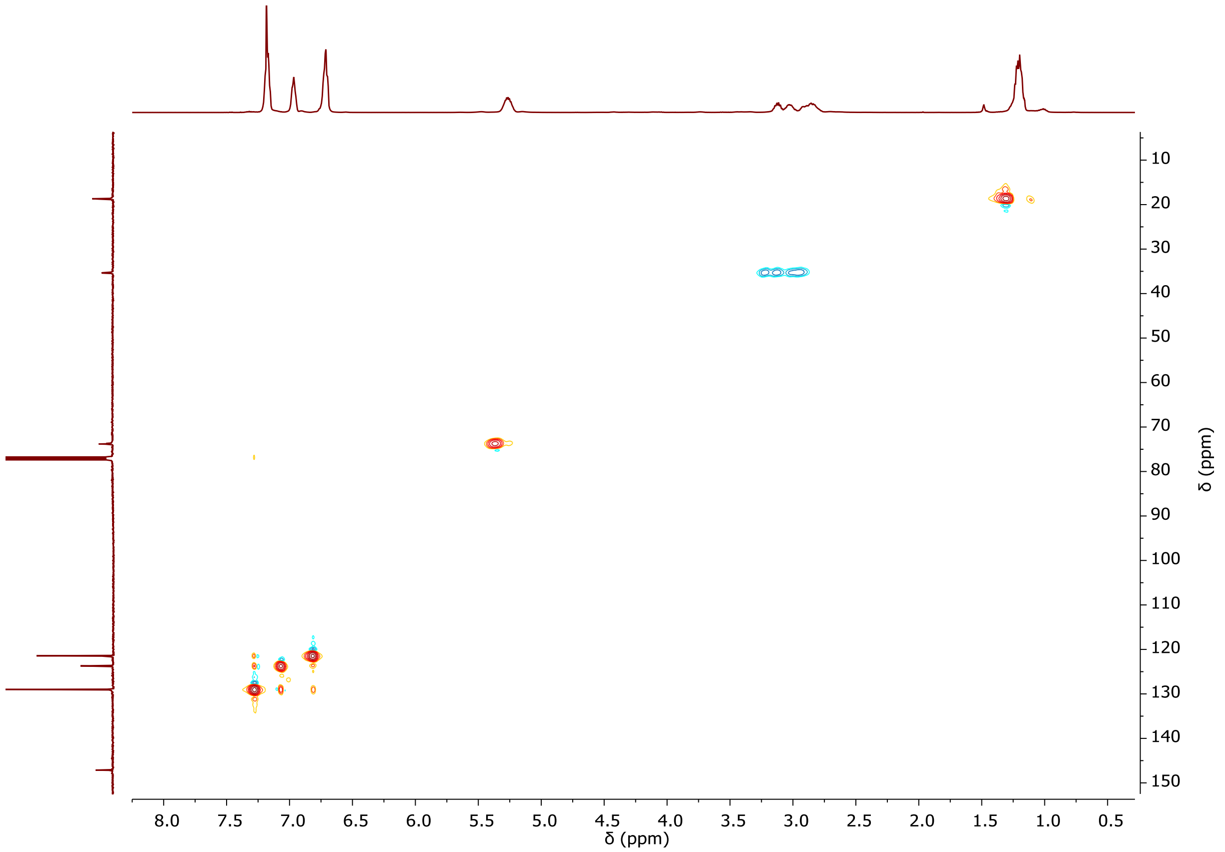

Figure S 34: ^{1}H - ^{13}C HSQC NMR spectrum ($CDCl_3$) of isolated PhNCS/PO copolymer (table S7, run 1).

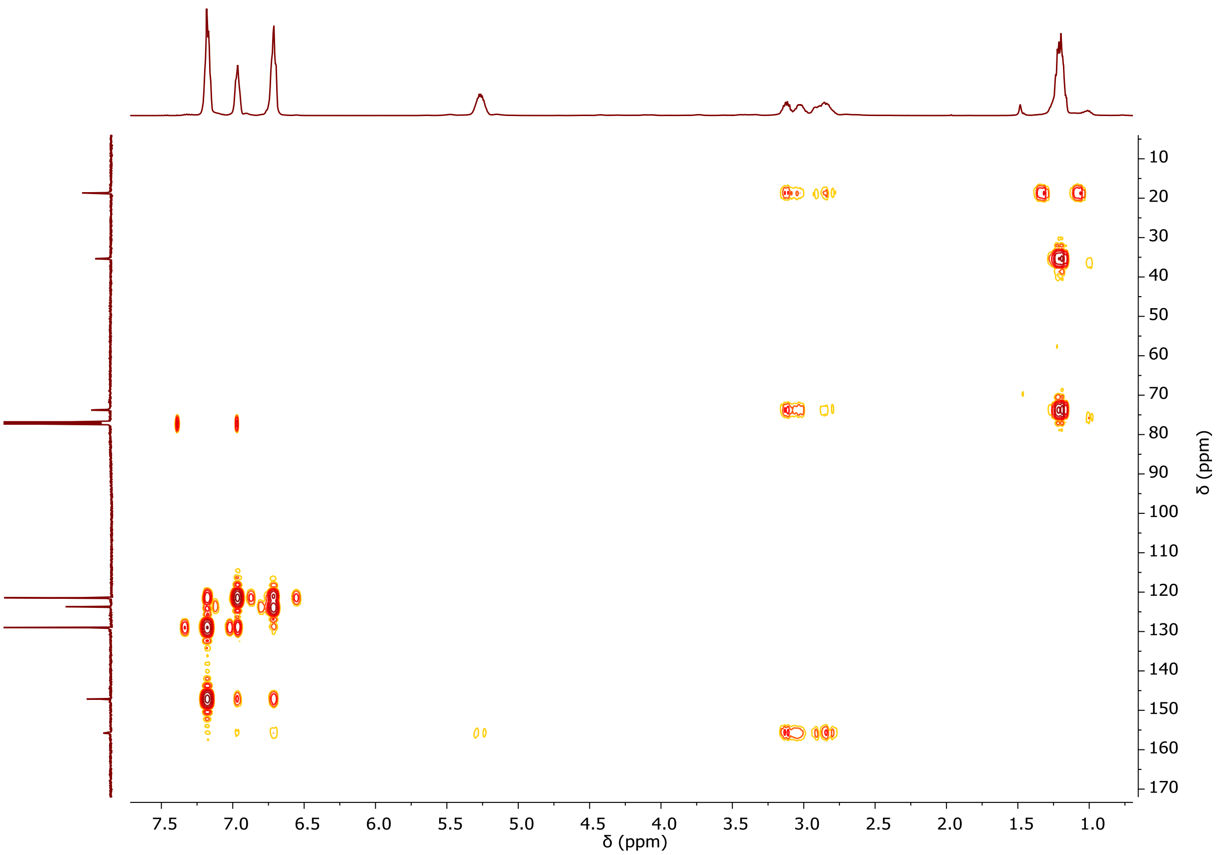

Figure S 35: ^{1}H - ^{13}C HMBC NMR spectrum ($CDCl_3$) of isolated PhNCS/PO copolymer (table S7, run 1).

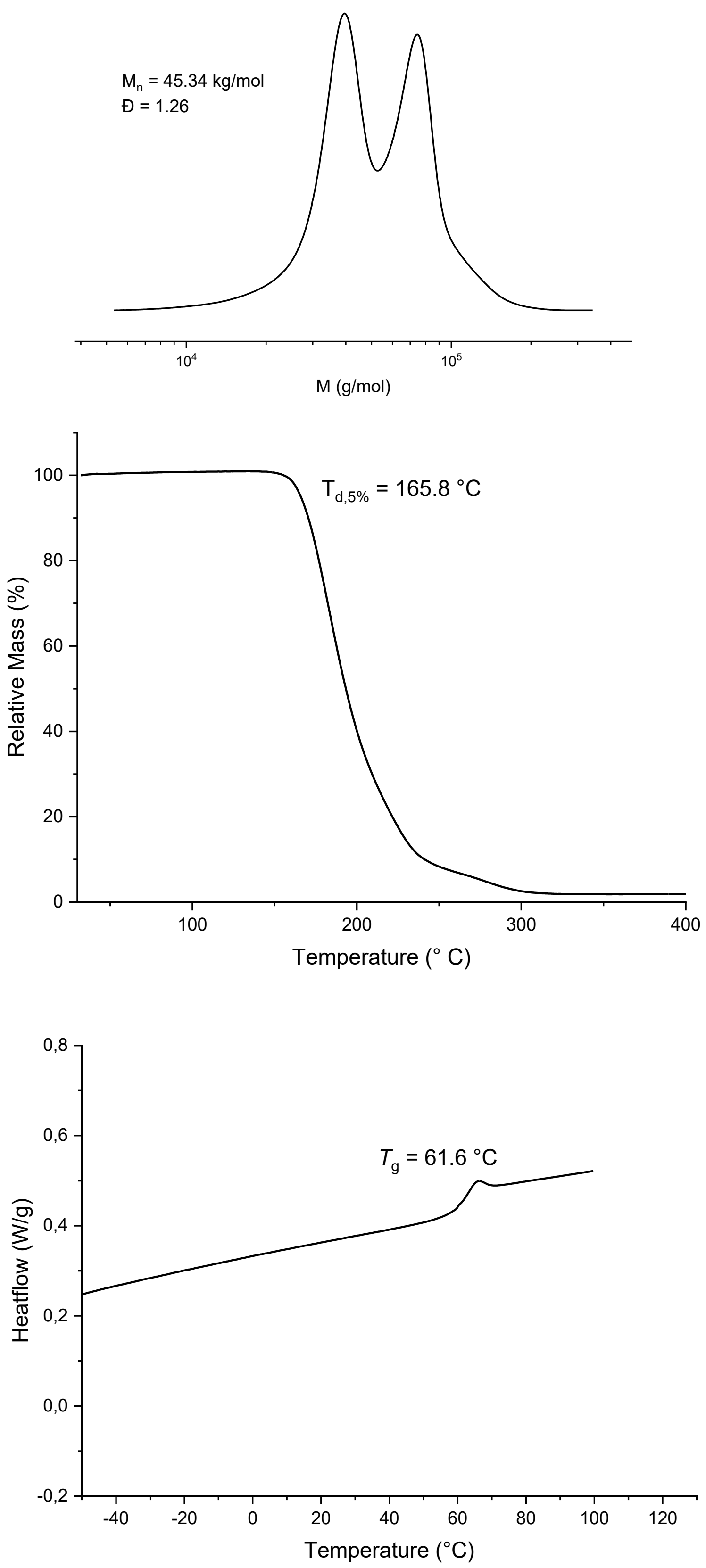

Figure S 36: GPC, TGA and DSC data for the isolated PhNCS/PO copolymer from table S7, run 1.

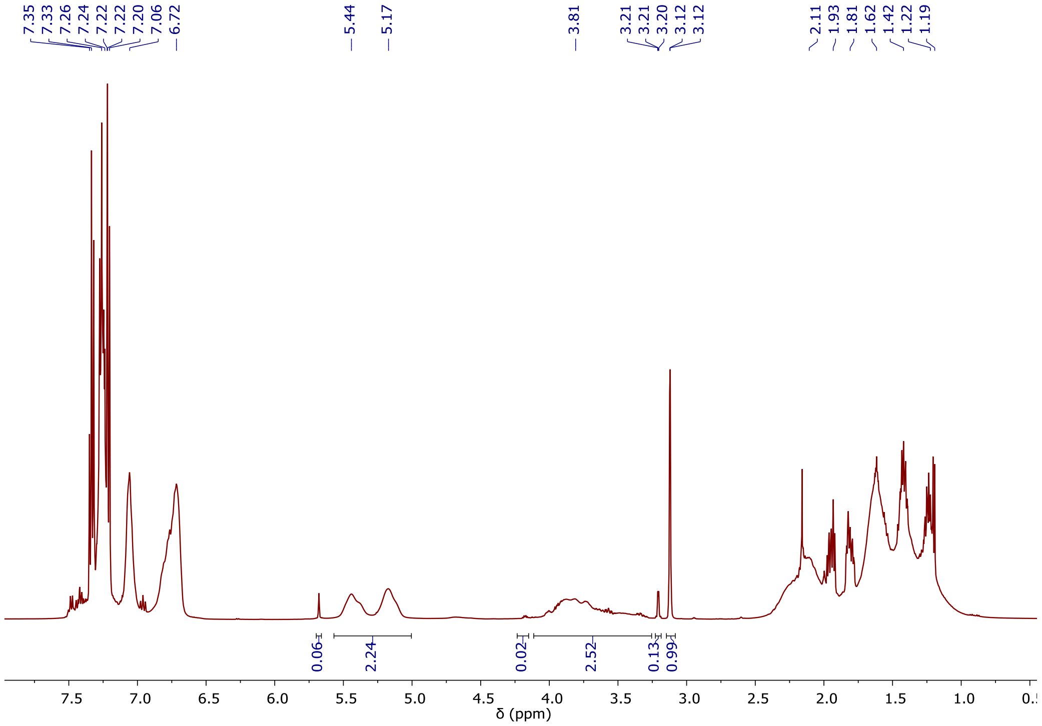

Figure S 37: ¹H NMR spectrum (500 MHz, $CDCl_3$) of the final aliquot from PhNCS/CHO ROCOP (table S7, run 2).

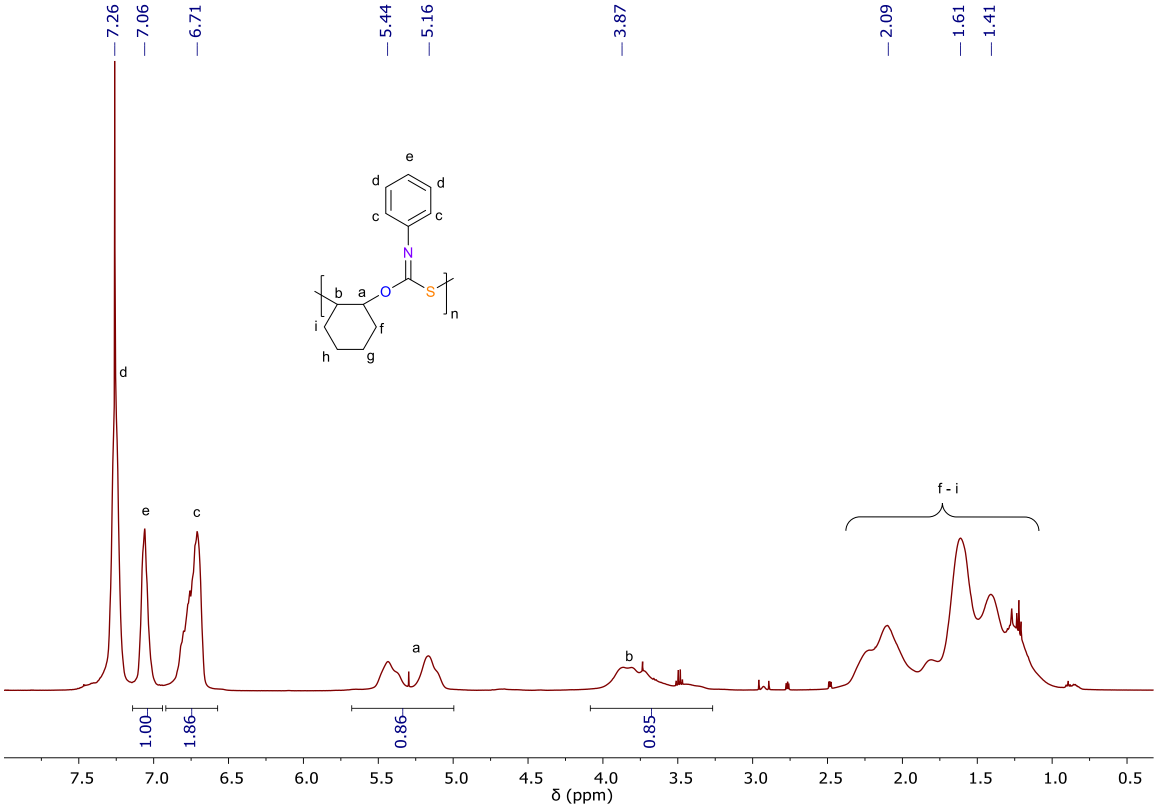

Figure S 38: ¹H NMR spectrum (500 MHz, $CDCl_3$) of the isolated PhNCS/CHO copolymer (table S7, run 2).

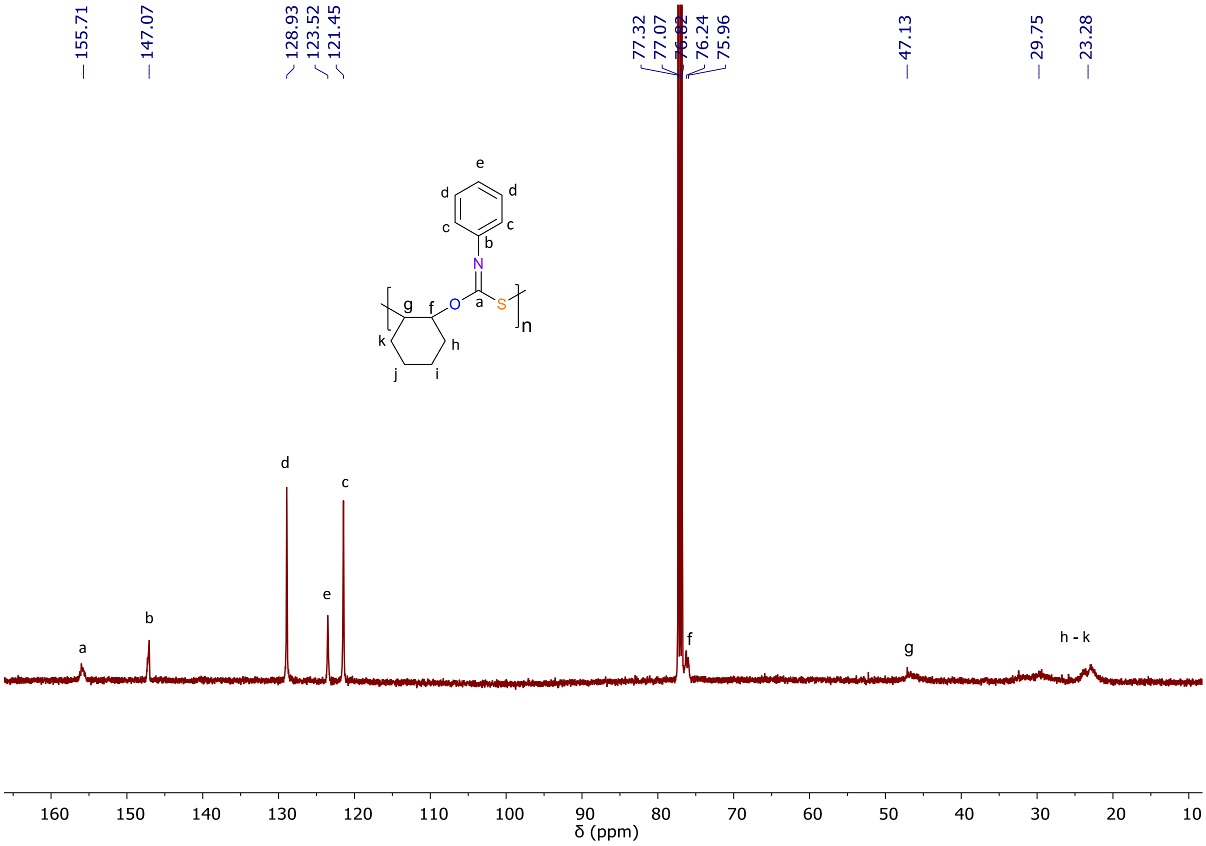

Figure S 39: ^{13}C NMR spectrum (125 MHz, $CDCl_3$) of the isolated PhNCS/CHO copolymer (table S7, run 2).

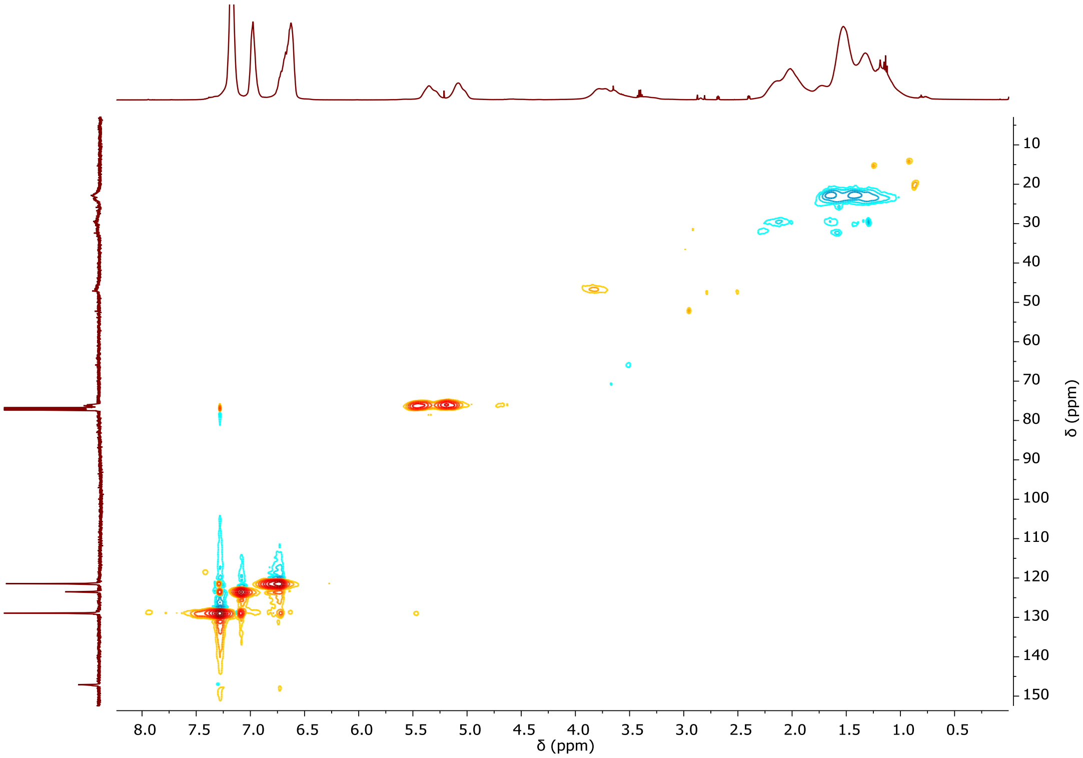

Figure S 40: ^{1}H - ^{13}C HSQC NMR spectrum ($CDCl_3$) of isolated PhNCS/CHO copolymer (table S7, run 2).

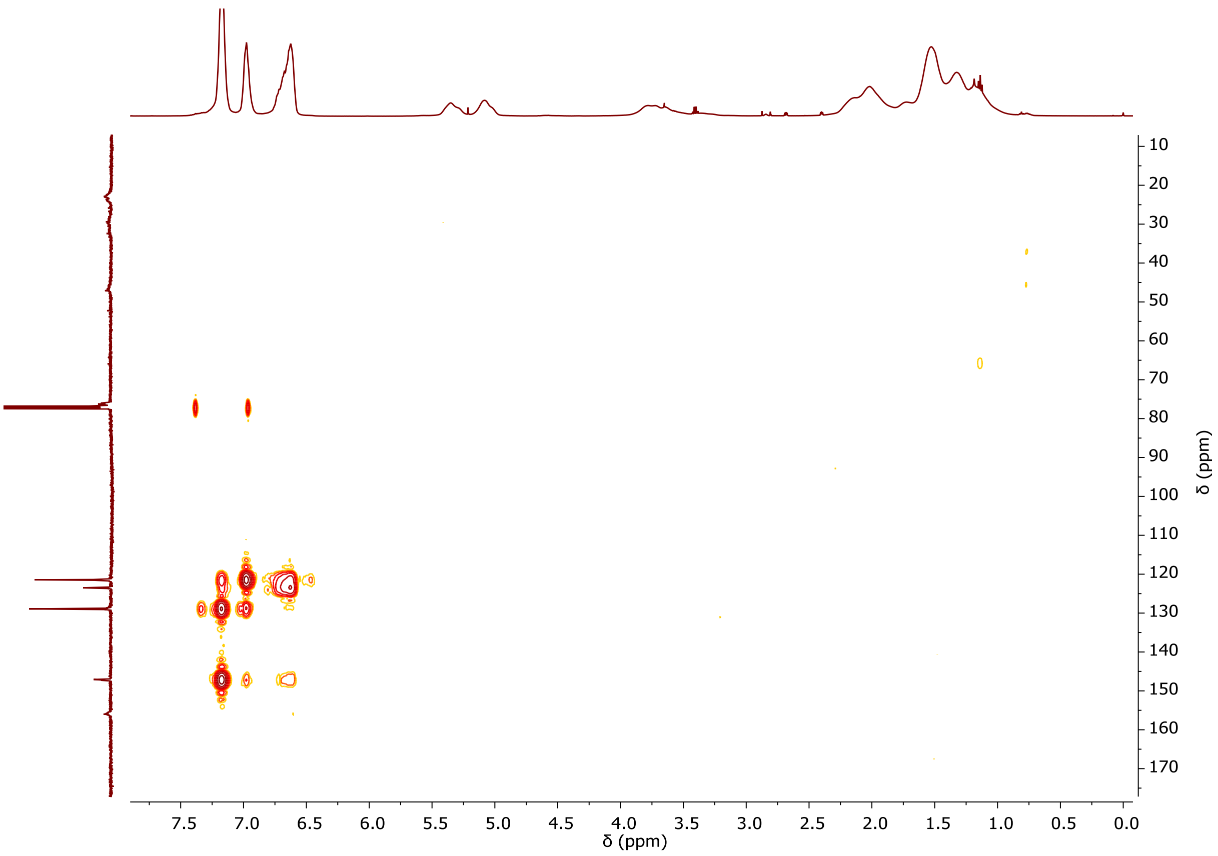

Figure S 41: ^{1}H - ^{13}C HMBC NMR spectrum ($CDCl_3$) of isolated PhNCS/CHO copolymer (table S7, run 2)

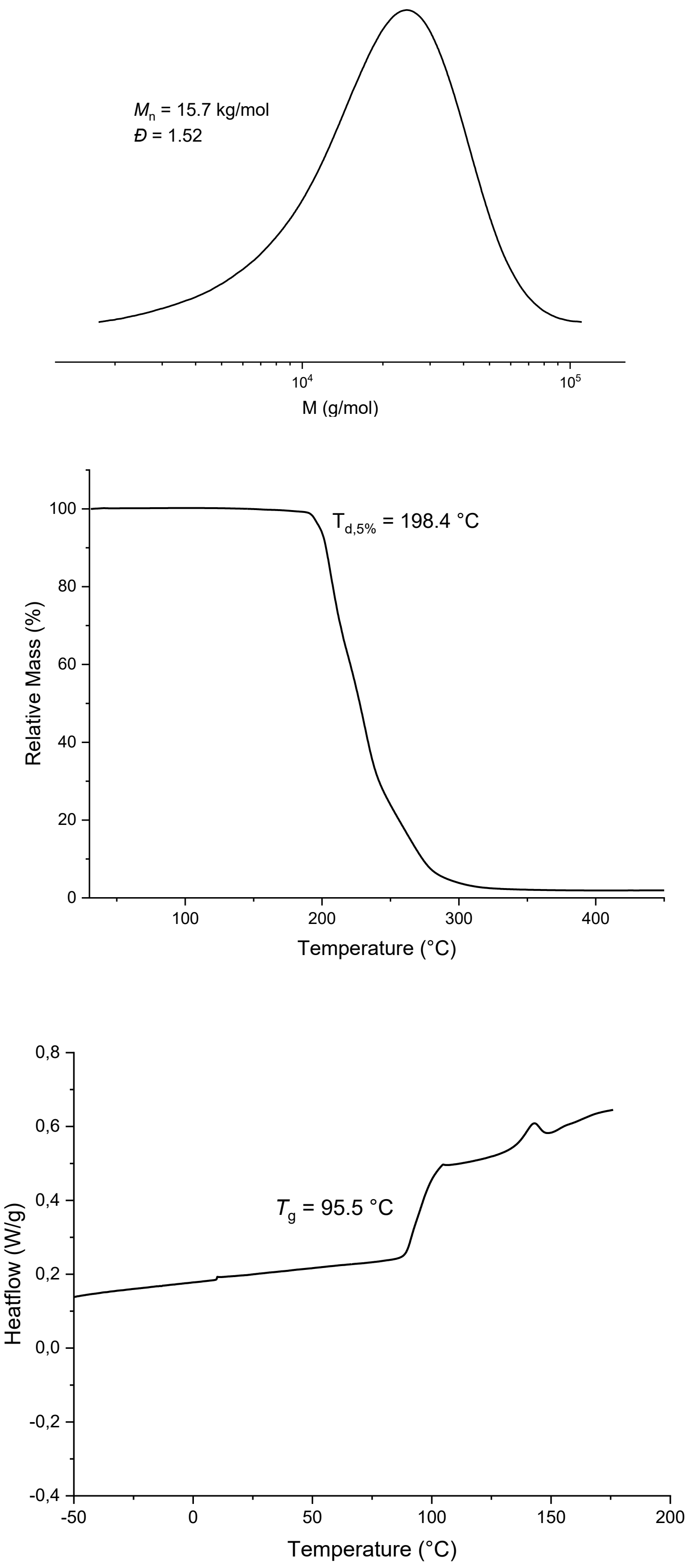

Figure S 42: GPC, TGA and DSC data for the isolated PhNCS/CHO copolymer from table S7, run 2.

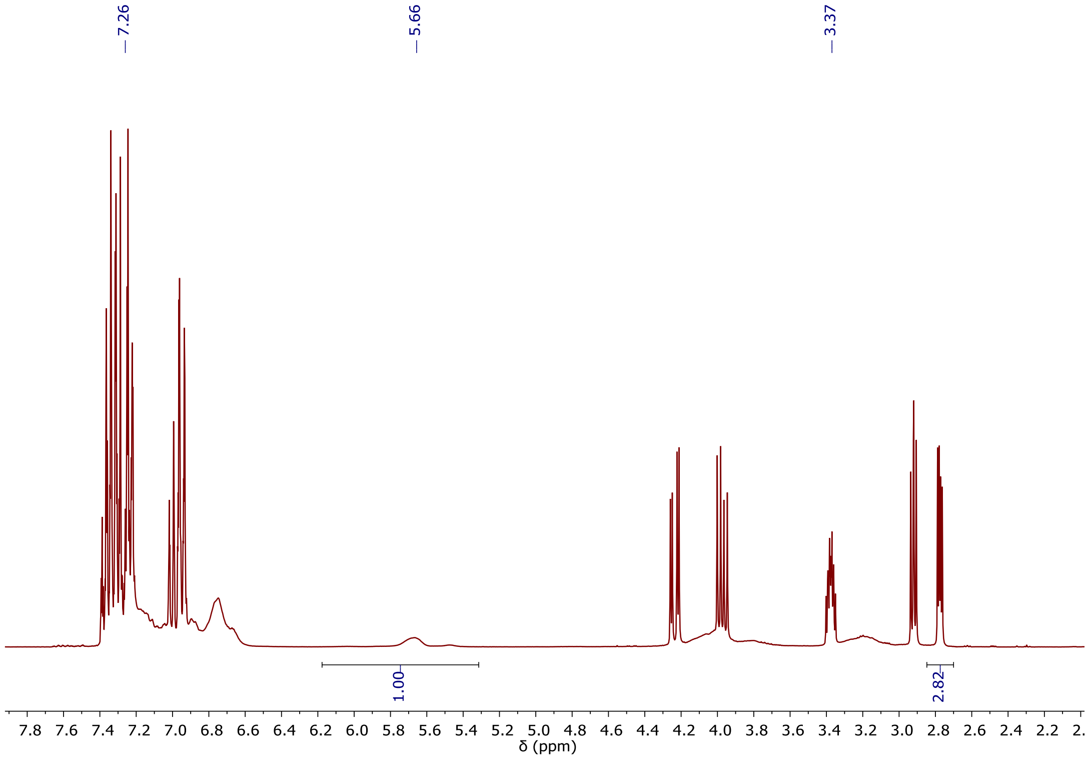

Figure S 43: ^{1}H NMR spectrum (300 MHz, $CDCl_3$) of the final aliquot from PhNCS/PGE ROCOP (table S7, run 3).

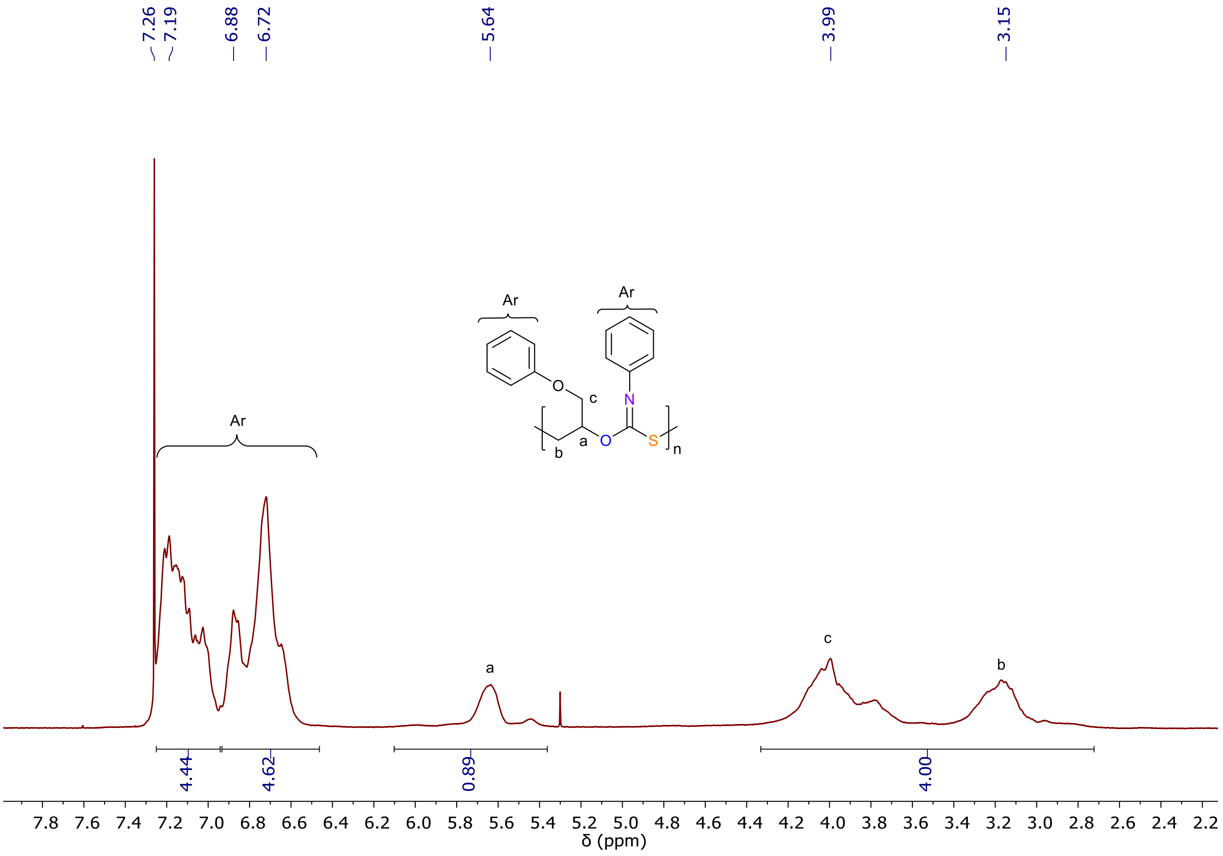

Figure S 44: ^{1}H NMR spectrum (500 MHz, $CDCl_3$) of the isolated PhNCS/PGE copolymer (table S7, run 3).

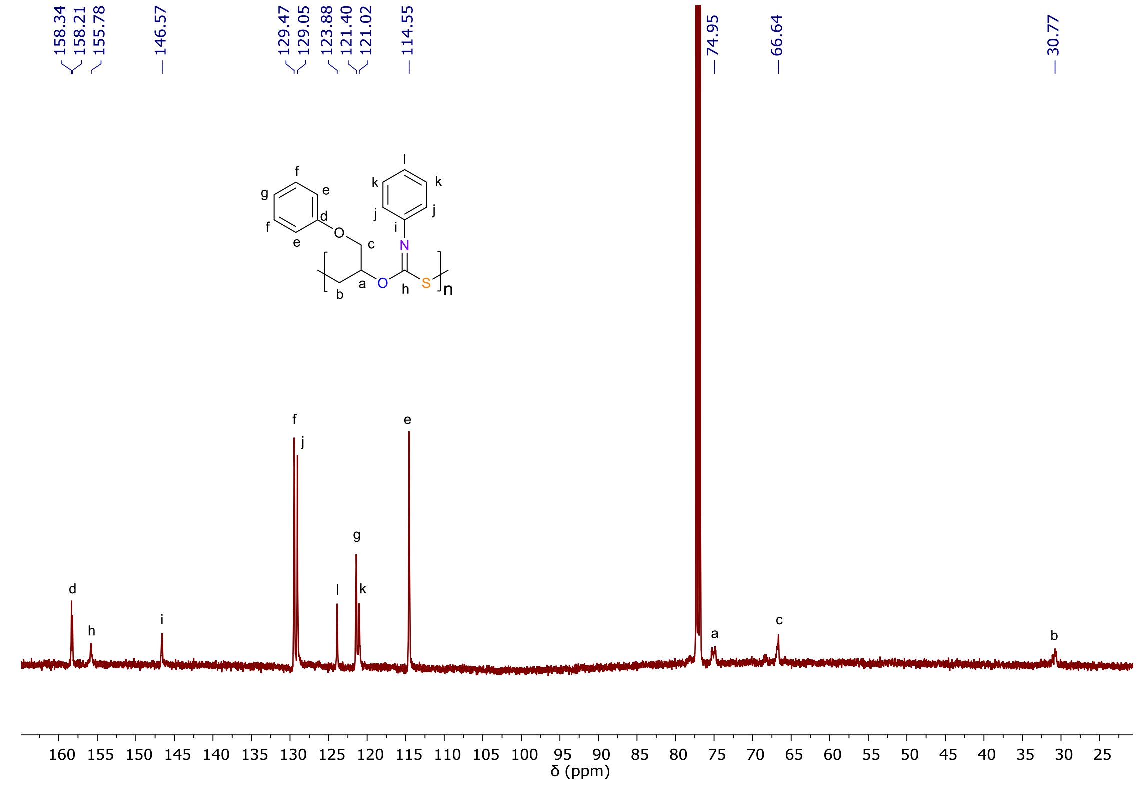

Figure S 45: ^{13}C NMR spectrum (125 MHz, $CDCl_3$) of the isolated PhNCS/PGE copolymer (table S7, run 3).

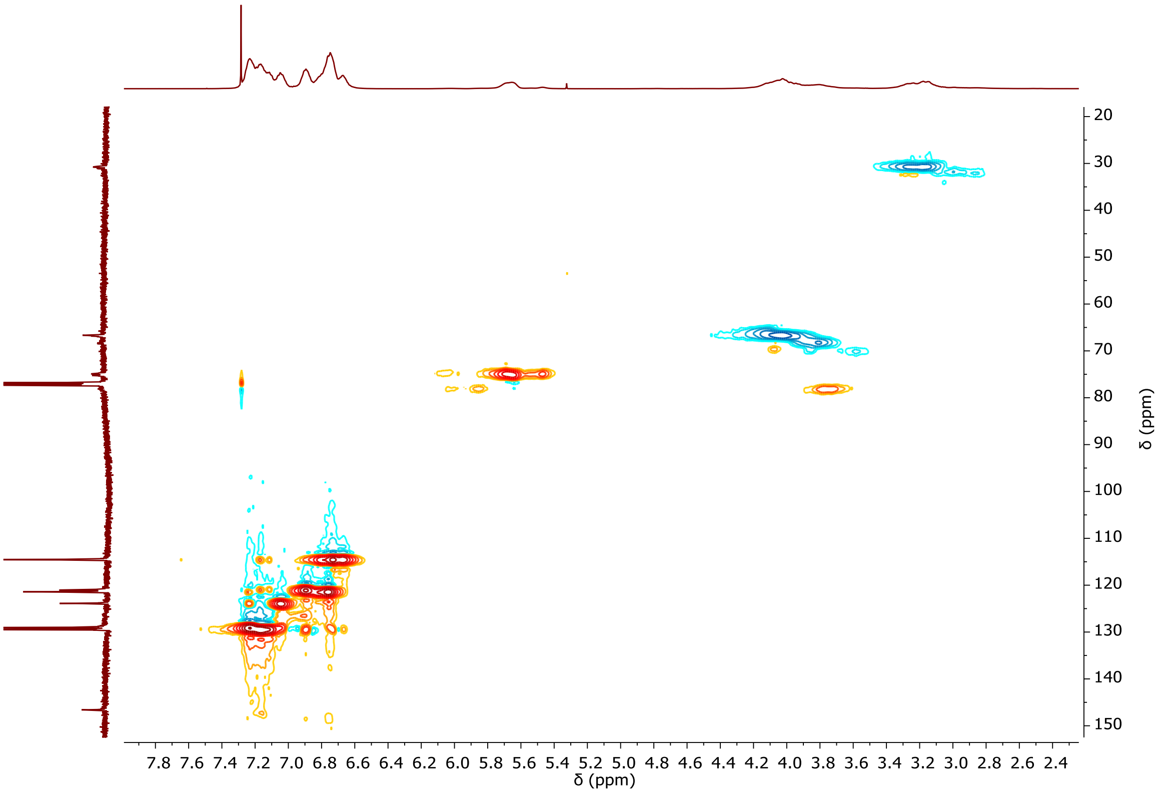

Figure S 46: ^{1}H - ^{13}C HSQC NMR spectrum ($CDCl_3$) of isolated PhNCS/PGE copolymer (table S7, run 3).

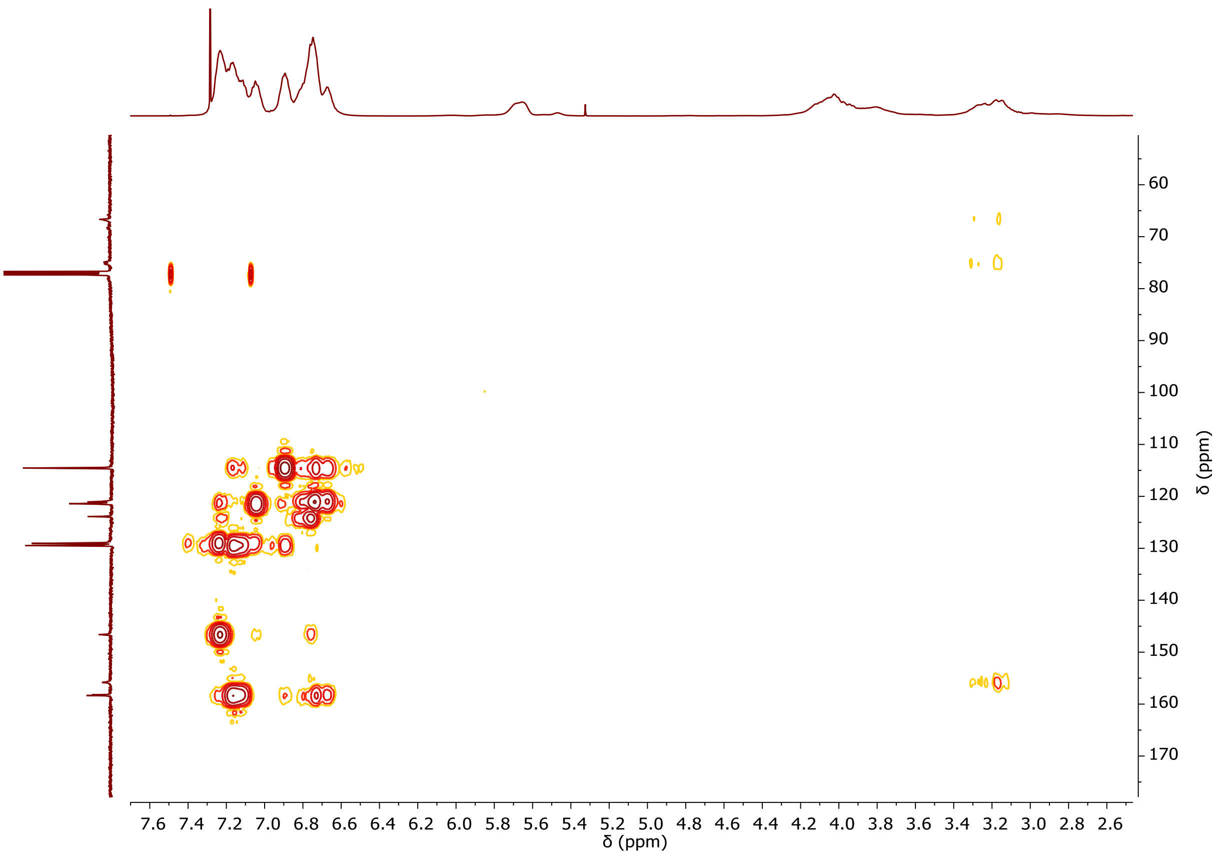

Figure S 47: ^{1}H - ^{13}C HMBC NMR spectrum ($CDCl_3$) of isolated PhNCS/PGE copolymer (table S7, run 3).

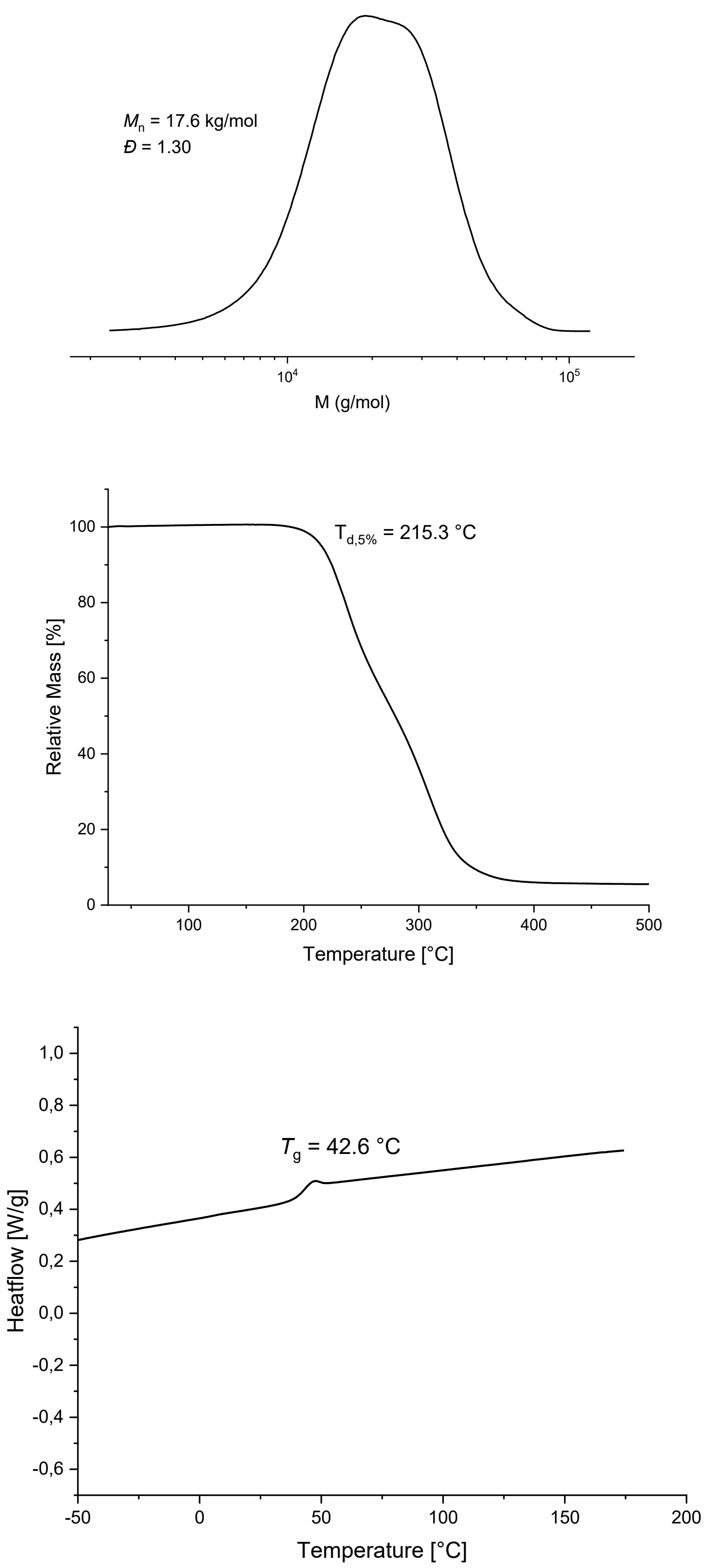

Figure S 48: GPC, TGA and DSC data for the isolated PhNCS/PGE copolymer from table S7, run 3.

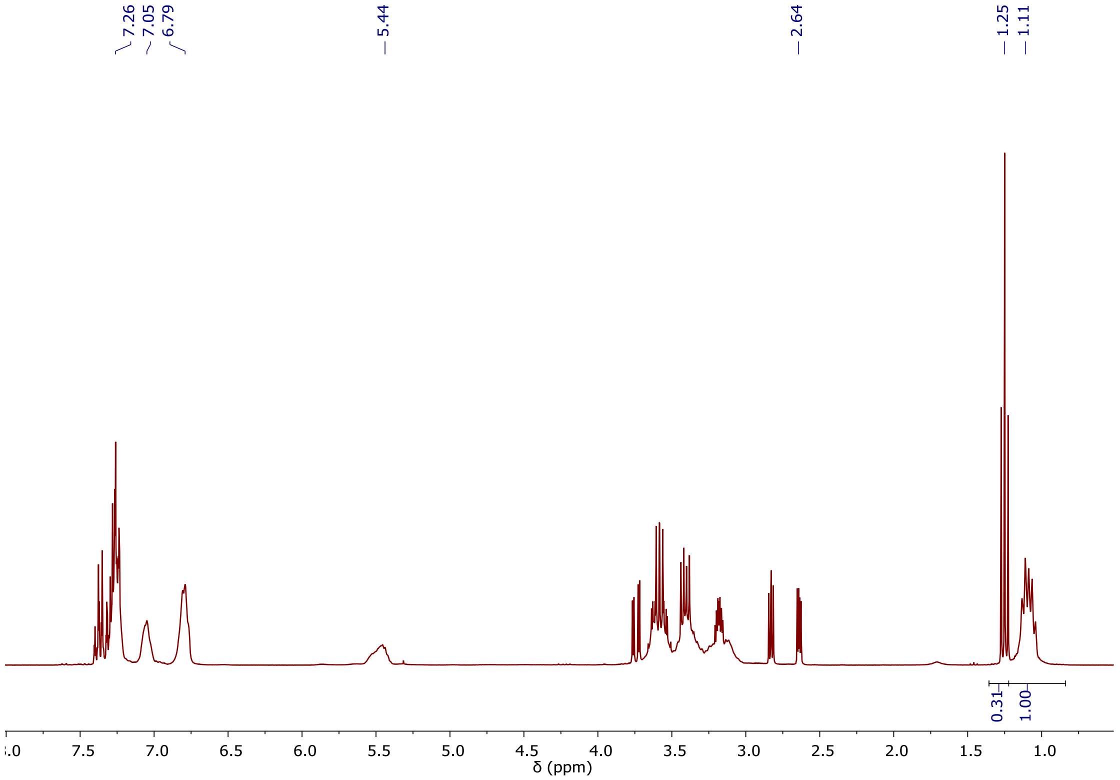

Figure S 49: ^{1}H NMR spectrum (300 MHz, $CDCl_3$) of the final aliquot from PhNCS/EGE ROCOP (table S7, run 4).

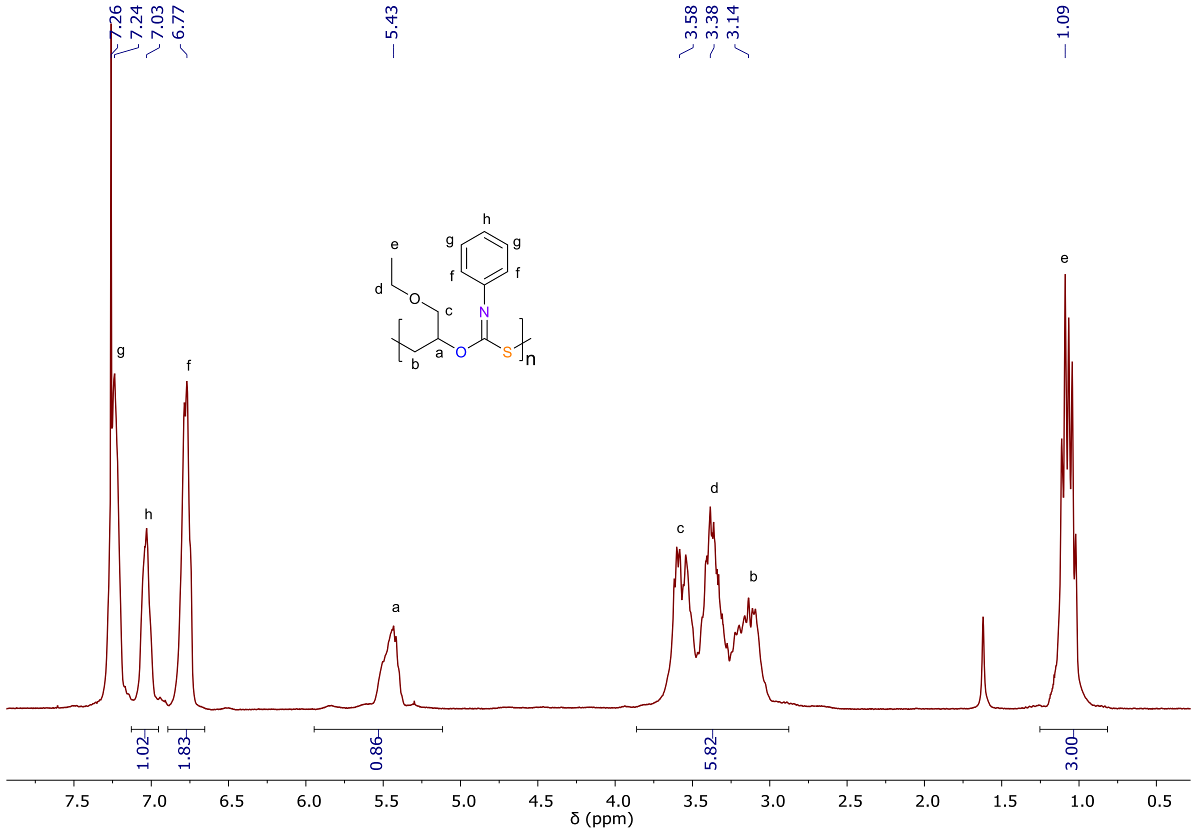

Figure S 50: ^{1}H NMR spectrum (500 MHz, $CDCl_3$) of the isolated PhNCS/EGE copolymer (table S7, run 4).

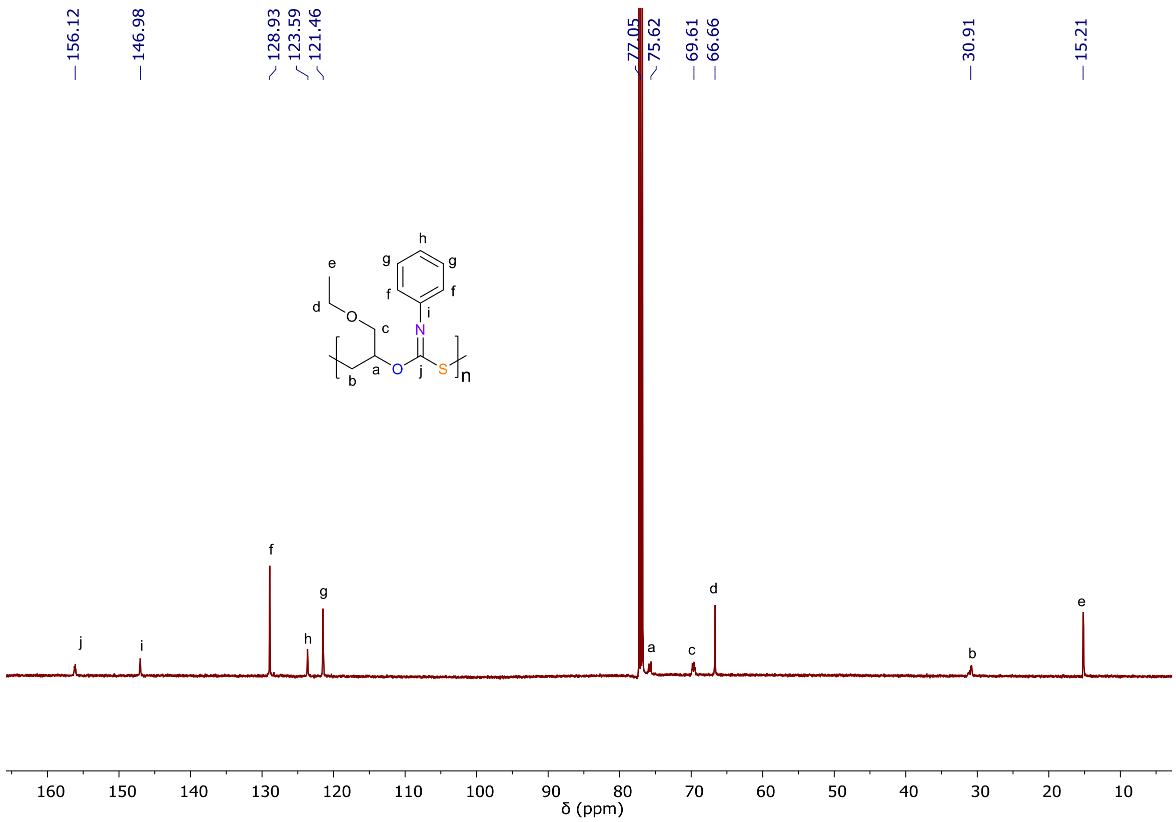

Figure S 51: ^{13}C NMR spectrum (125 MHz, $CDCl_3$) of the isolated PhNCS/EGE copolymer (table S7, run 4).

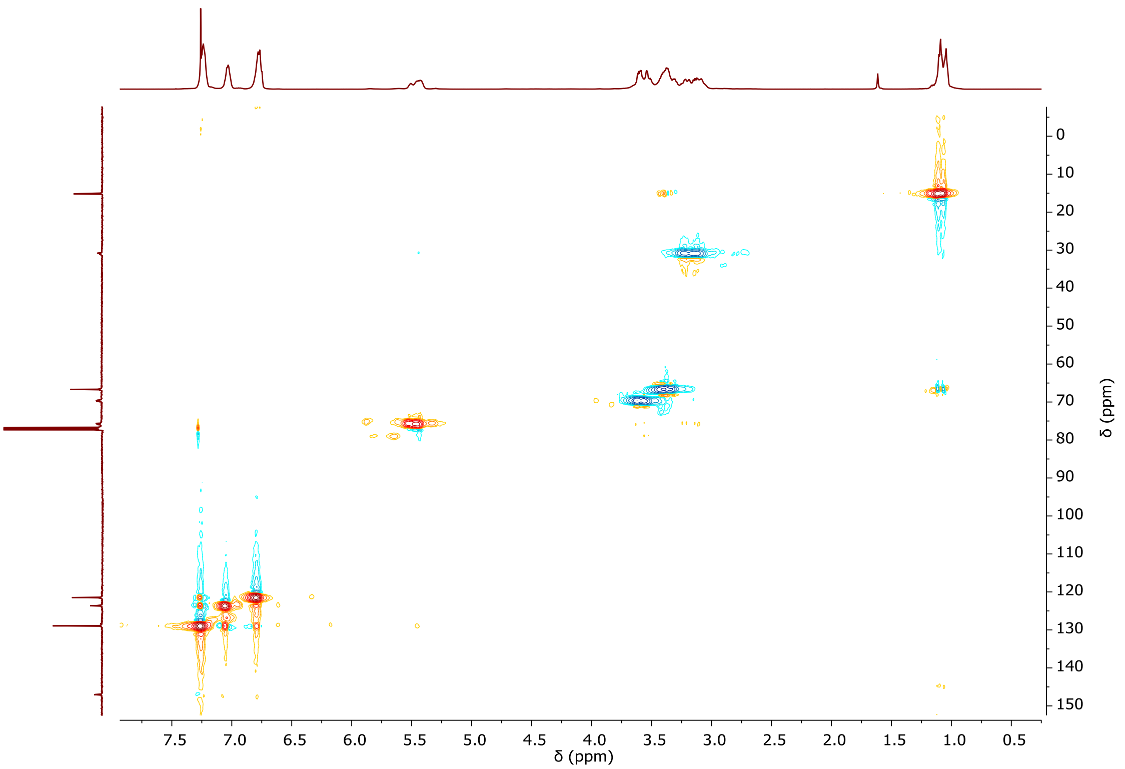

Figure S 52: ^{1}H - ^{13}C HSQC NMR spectrum ($CDCl_3$) of isolated PhNCS/EGE copolymer (table S7, run 4).

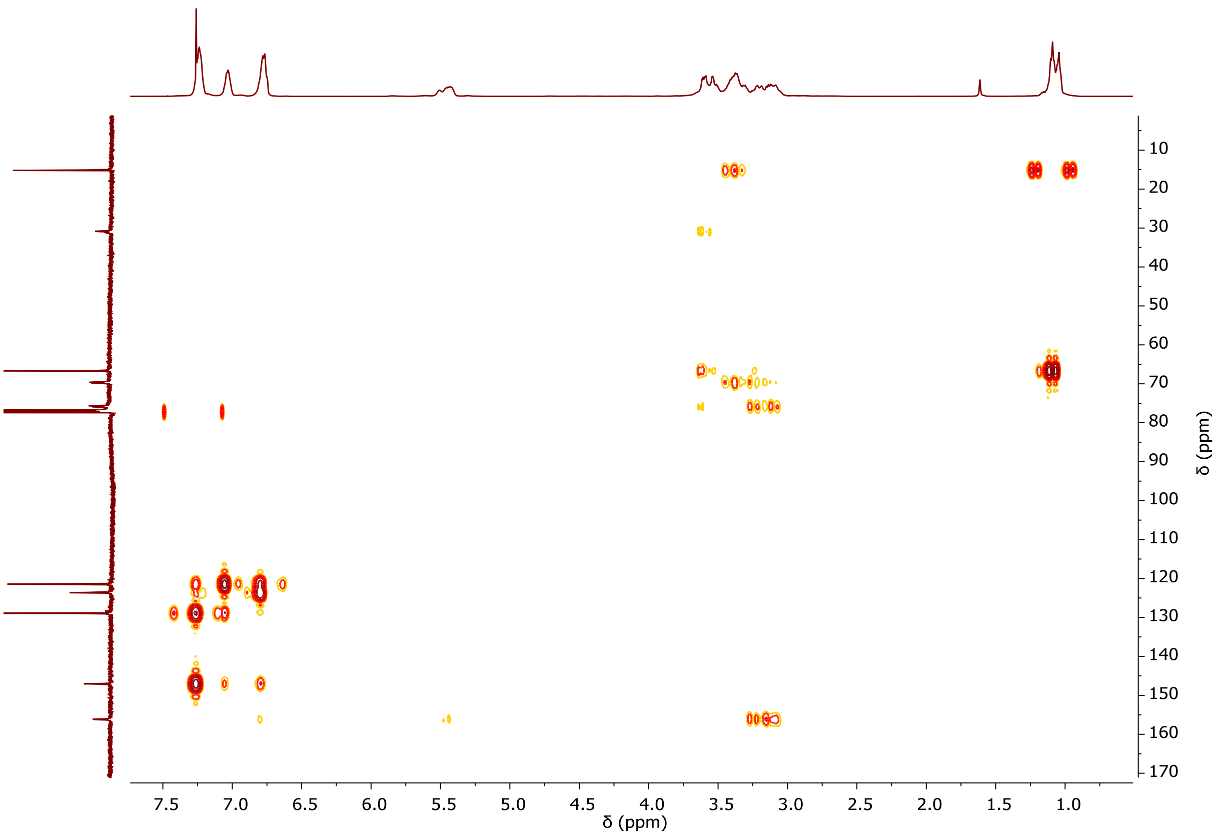

Figure S 53: ^{1}H - ^{13}C HMQC NMR spectrum ($CDCl_3$) of isolated PhNCS/EGE copolymer (table S7, run 4).

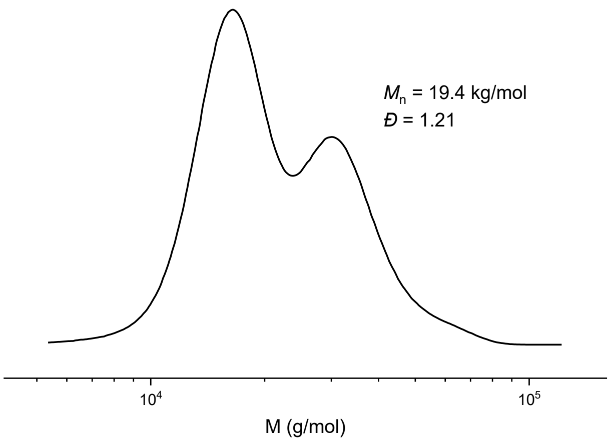

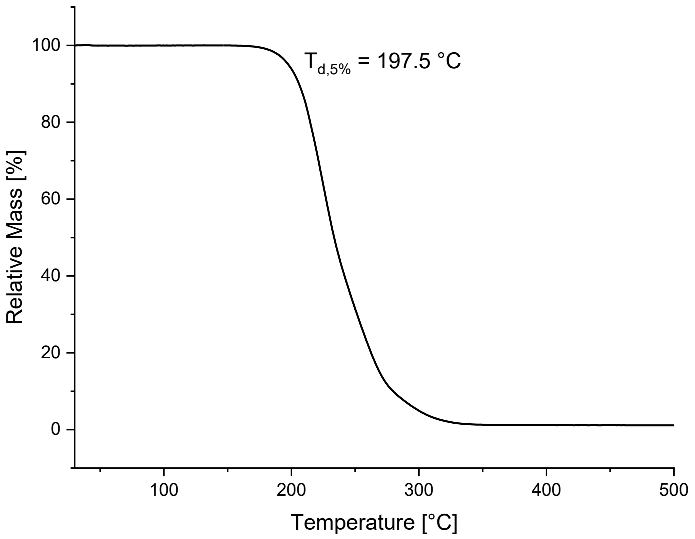

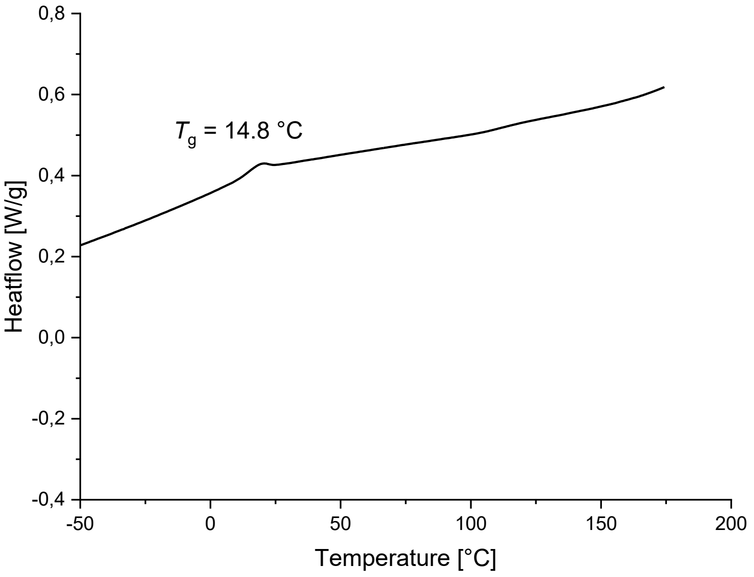

Figure S 54: GPC, TGA and DSC data for the isolated PhNCS/EGE copolymer from table S7, run 4

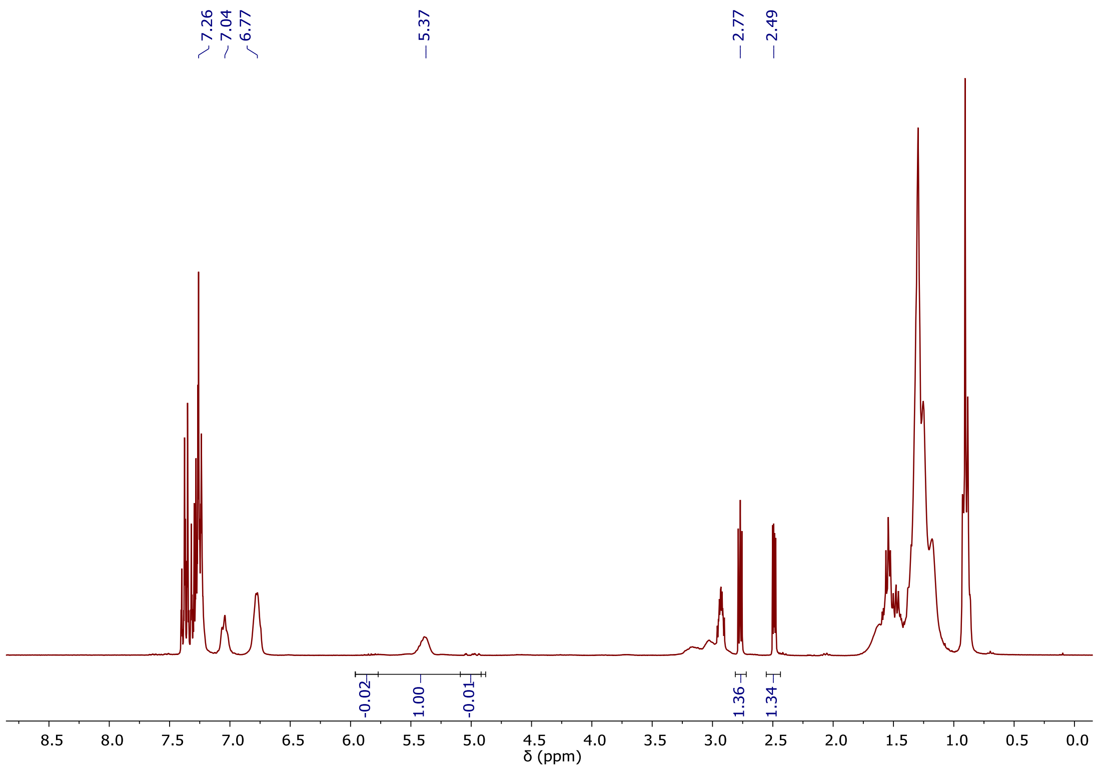

Figure S 55: ^{1}H NMR spectrum (300 MHz, $CDCl_3$) of the final aliquot from PhNCS/DO ROCOP (table S7, run 5).

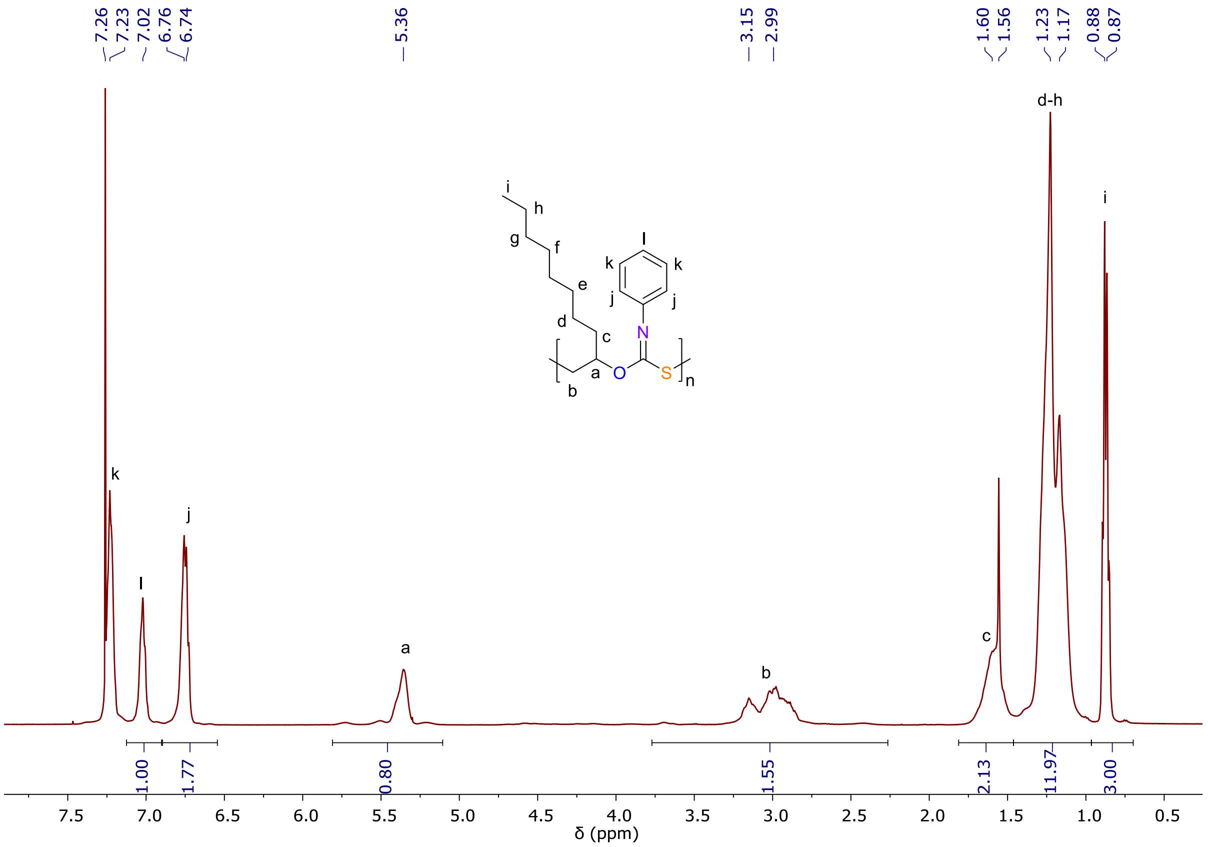

Figure S 56: ^{1}H NMR spectrum (500 MHz, $CDCl_3$) of the isolated PhNCS/DO copolymer (table S7, run 5).

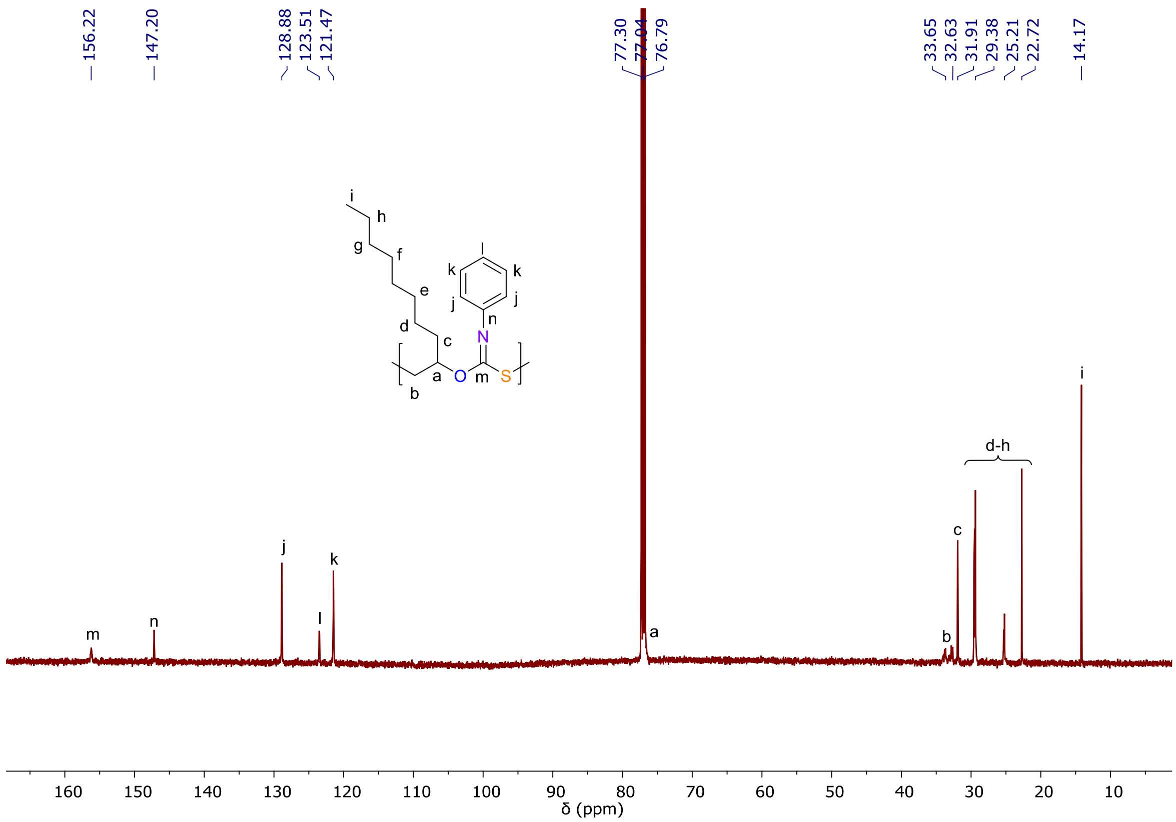

Figure S 57: ^{13}C NMR spectrum (125 MHz, $CDCl_3$) of the isolated PhNCS/DO copolymer (table S7, run 5).

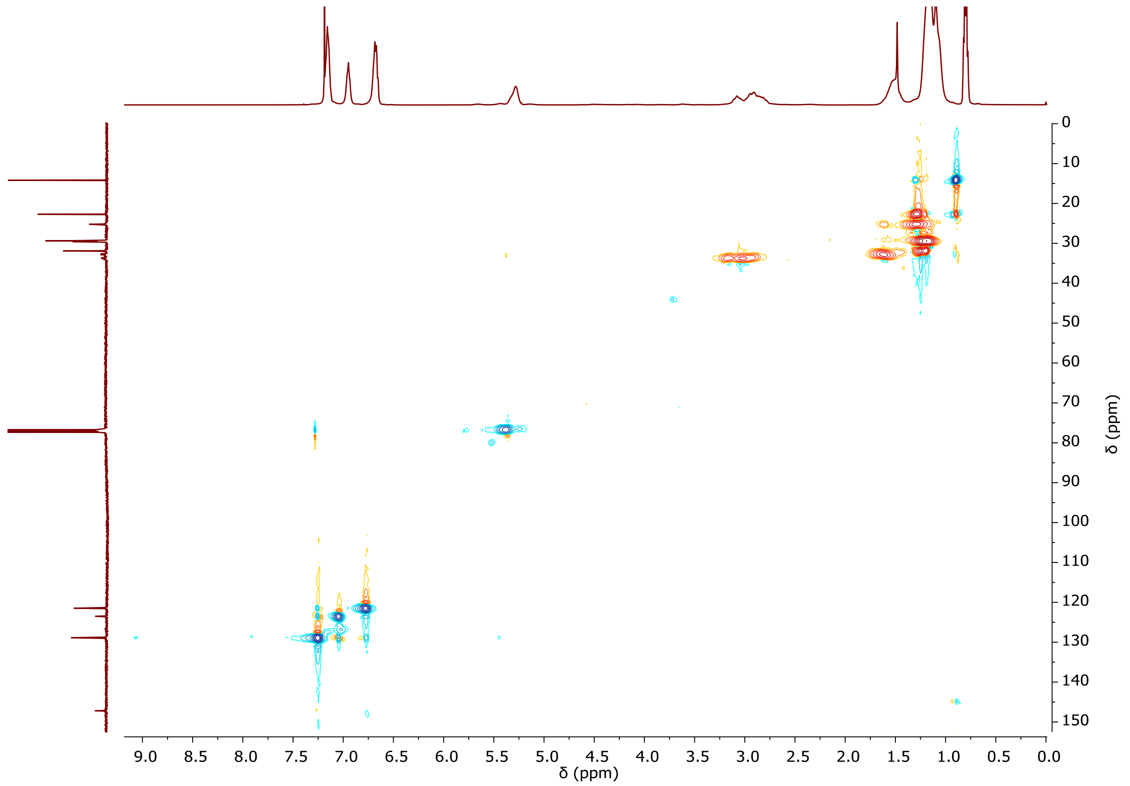

Figure S 58: ^{1}H - ^{13}C HSQC NMR spectrum ($CDCl_3$) of isolated PhNCS/DO copolymer (table S7, run 5).

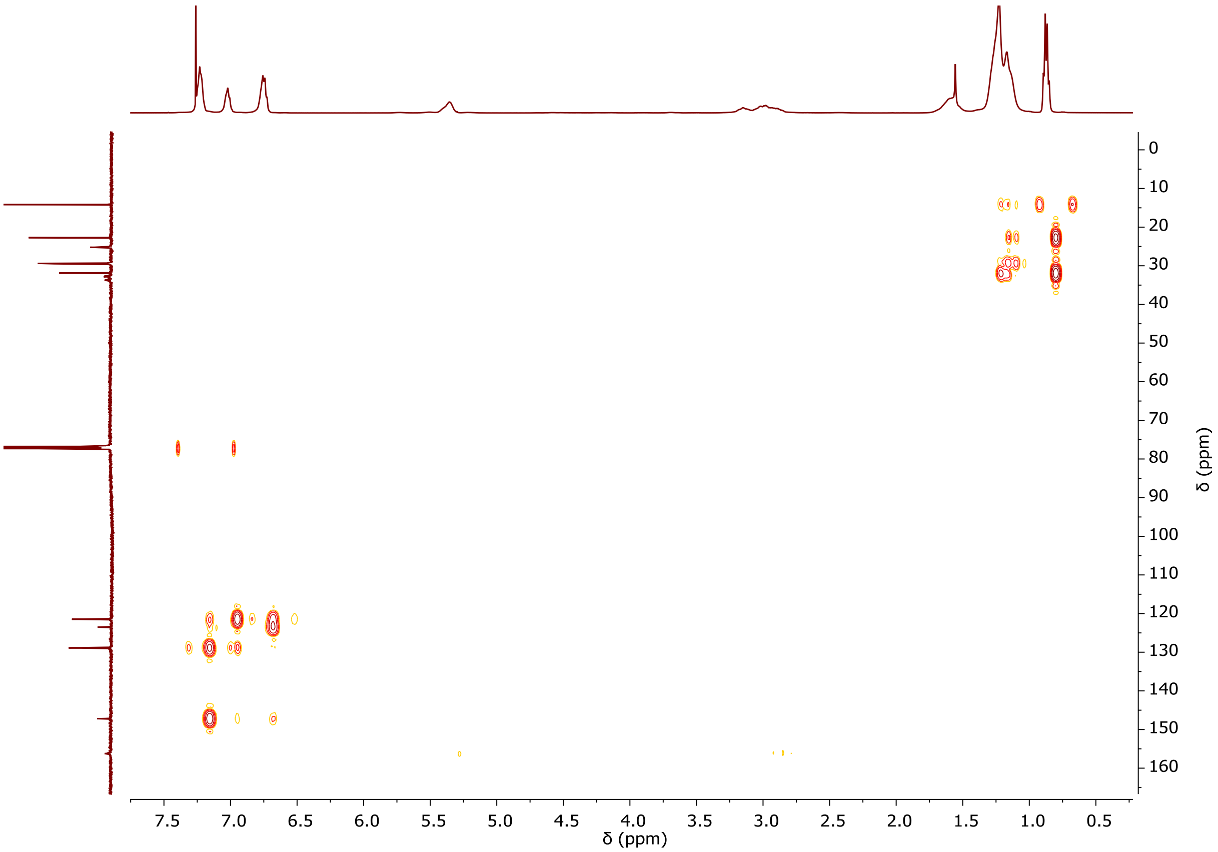

Figure S 59: ^{1}H - ^{13}C HMBC NMR spectrum ($CDCl_3$) of isolated PhNCS/DO copolymer (table S7, run 5).

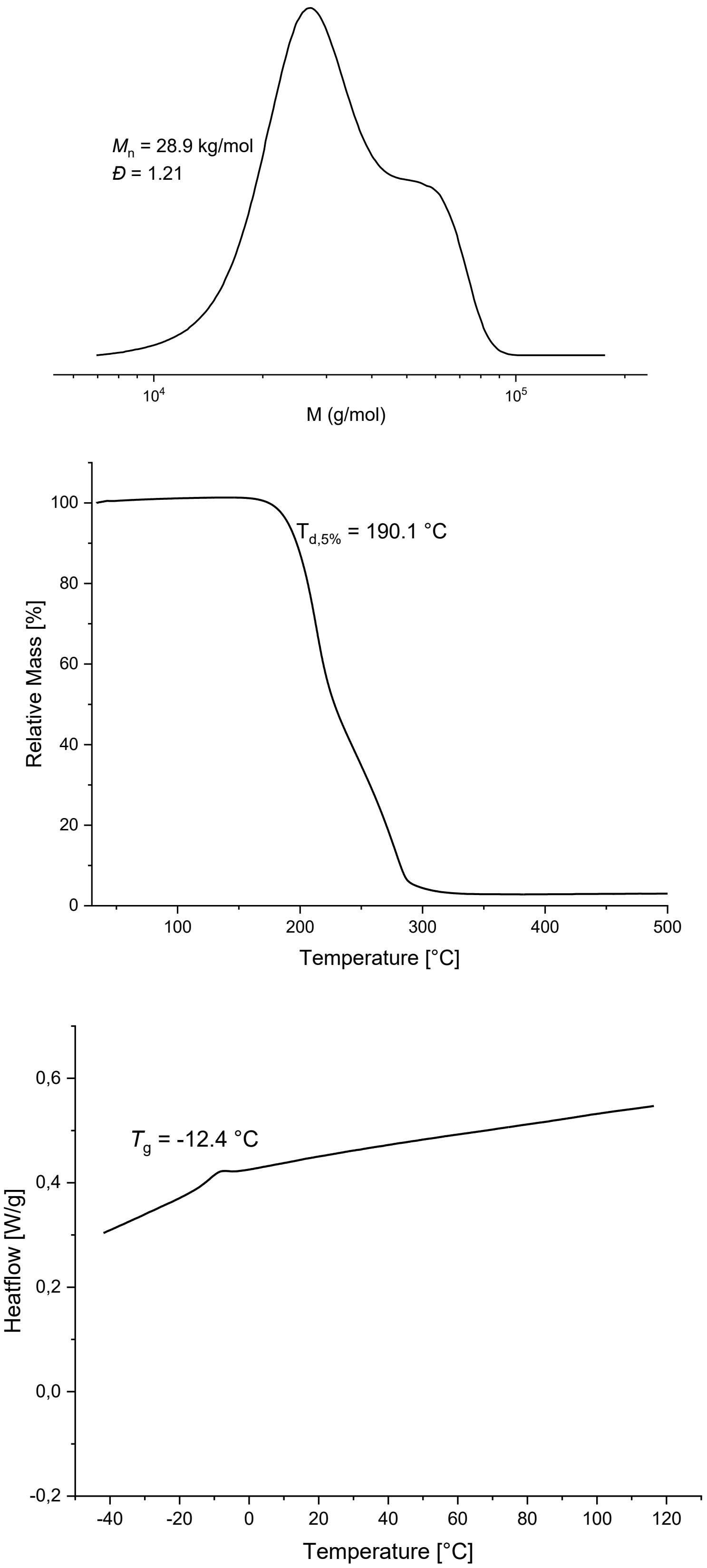

Figure S 60: GPC, TGA and DSC data for the isolated PhNCS/DO copolymer from table S7, run 5

h. COS/epoxide ROCOP

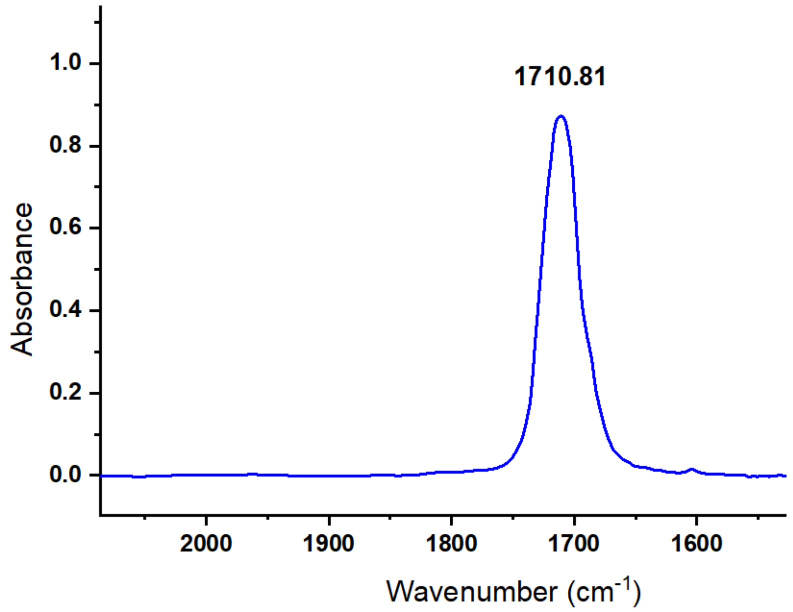

Figure S 61: Reaction mixture ATR FT-IR spectrum of CHO with COS in CH_2Cl_2 (table 1, run 5).

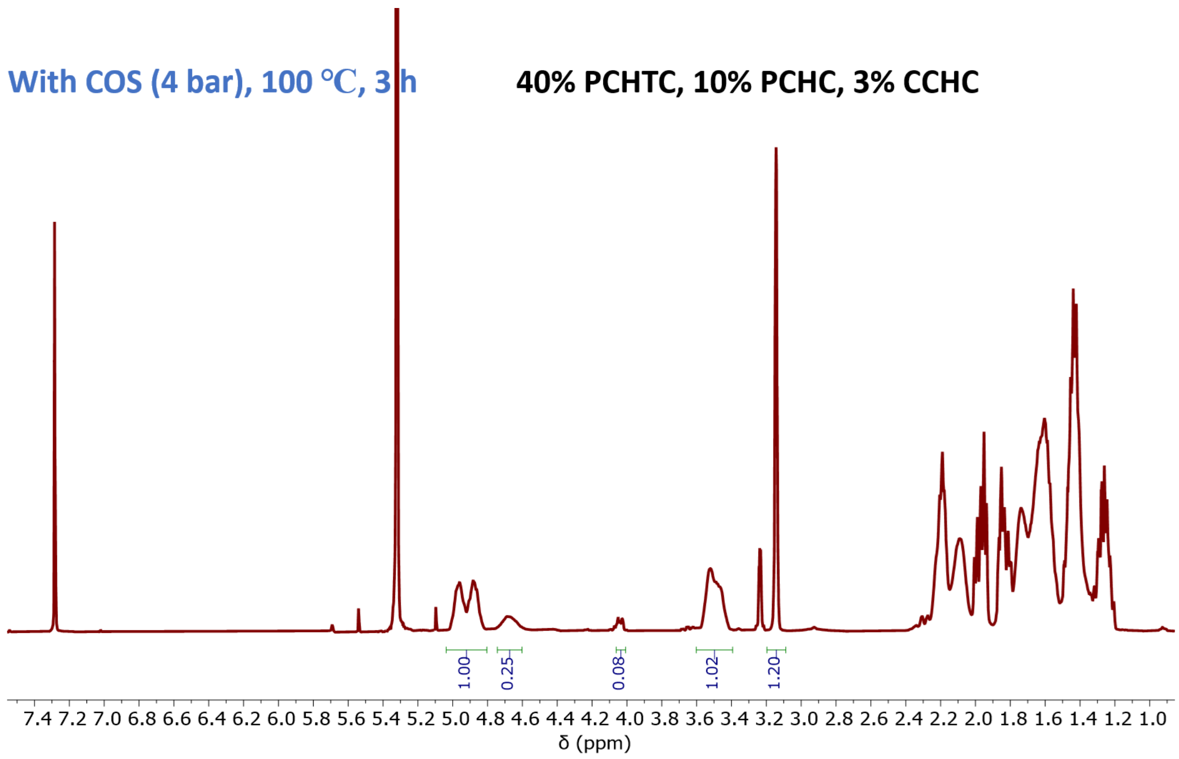

Figure S 62: Reaction mixture ^{1}H NMR of CHO with COS ($CDCl_3$, 400 MHz) (table 1, run 1).

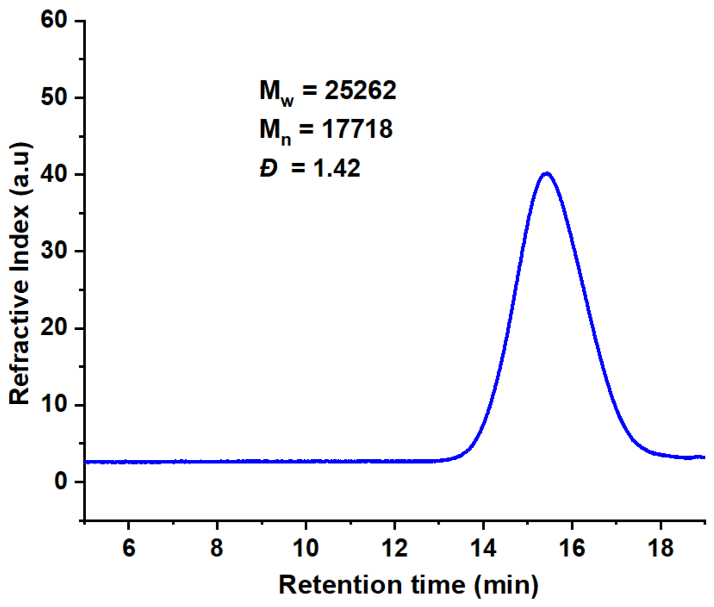

Figure S 63: GPC traces for polymonothiocarbonate (table 1, run 1).

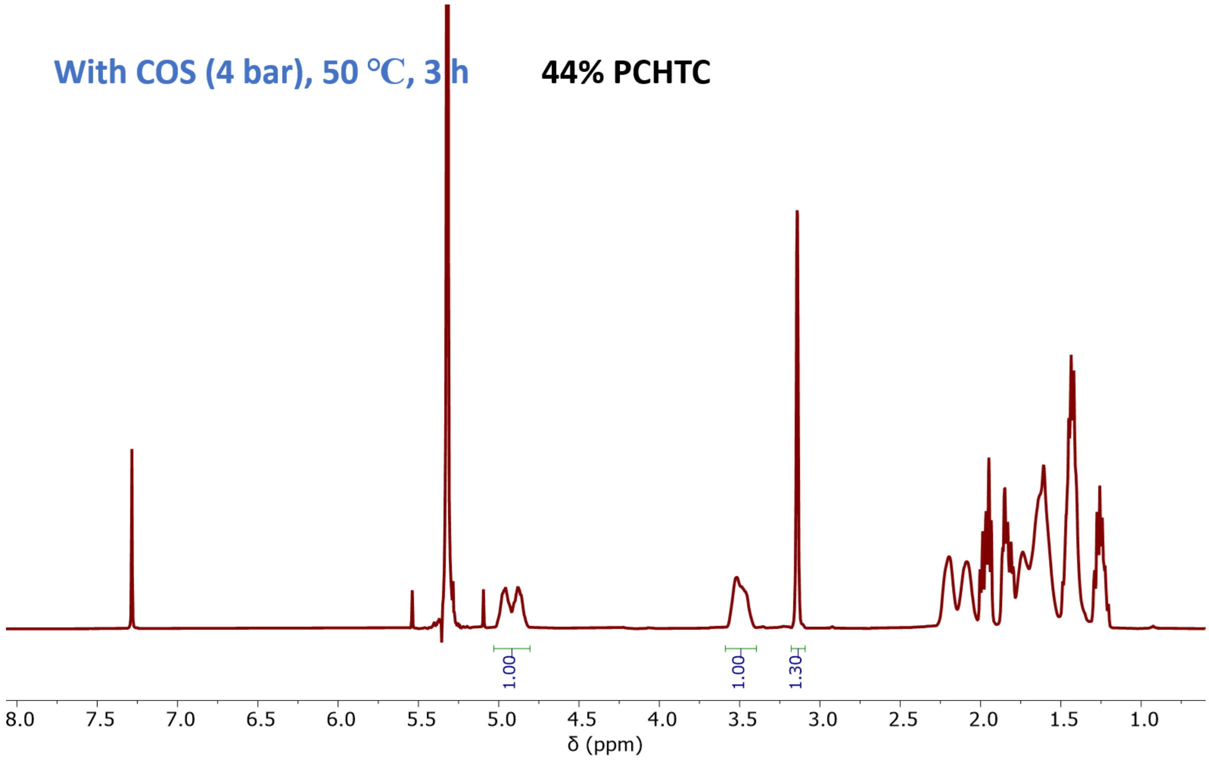

Figure S 64: Reaction mixture ^{1}H NMR of CHO with COS ($CDCl_3$, 400 MHz) (table 1, run 2).

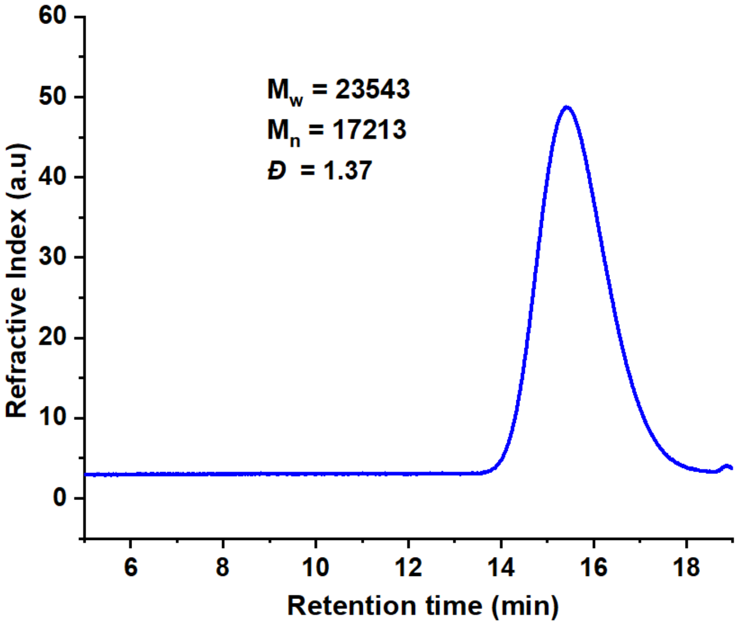

Figure S 65: GPC traces for polymonothiocarbonate (table 1, run 2).

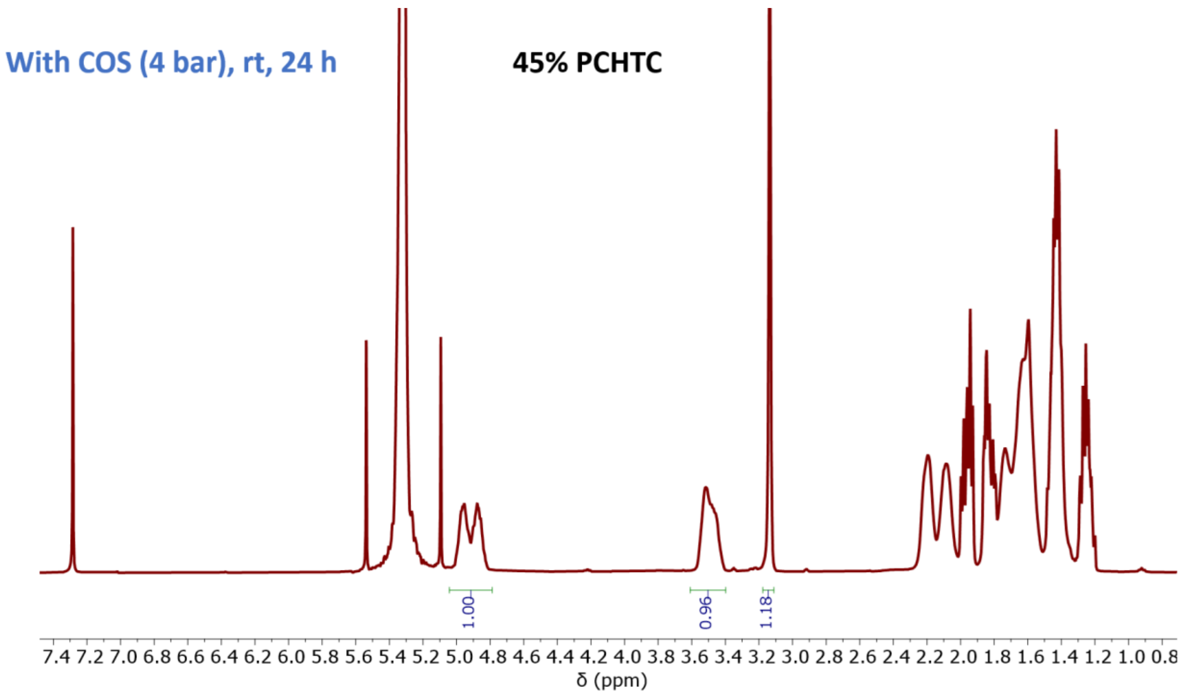

Figure S 66: Reaction mixture 1H NMR of CHO with COS (CDCl3, 400 MHz) (table 1, run 3).

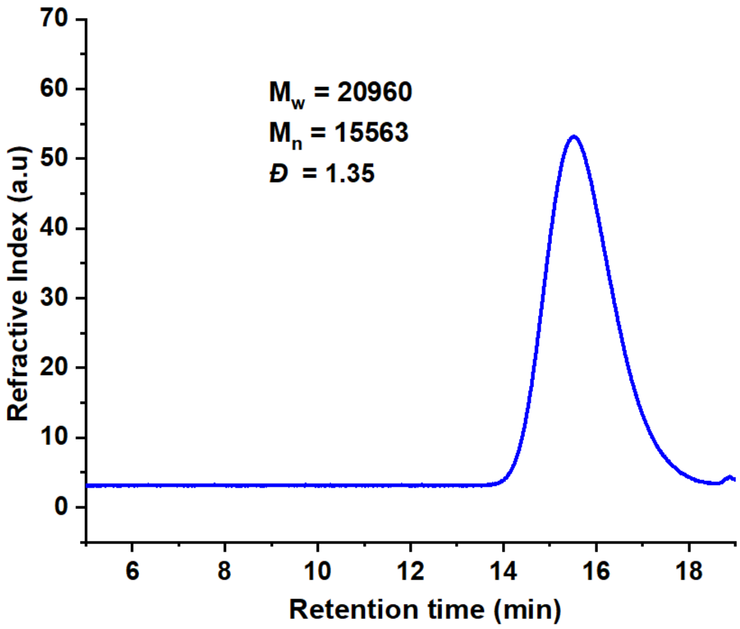

Figure S 67: GPC traces for polymonothiocarbonate (table 1, run 3).

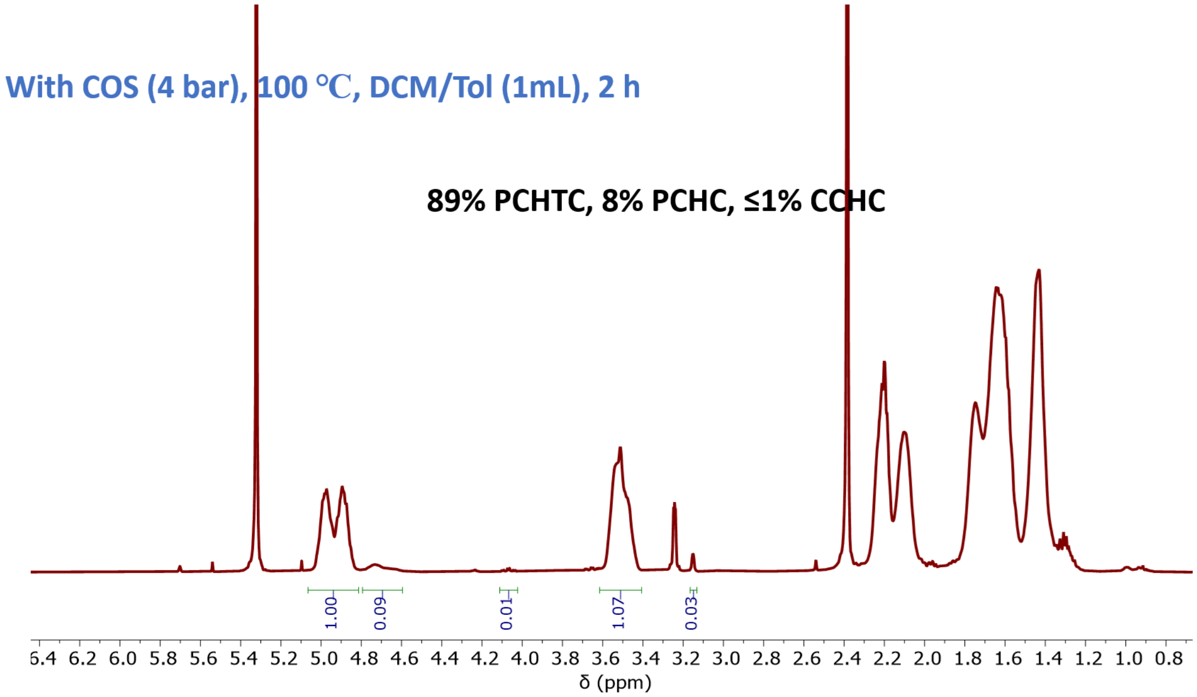

Figure S 68: Reaction mixture ^{1}H NMR of CHO with COS ($CDCl_3$, 400 MHz) (table 1, run 4).

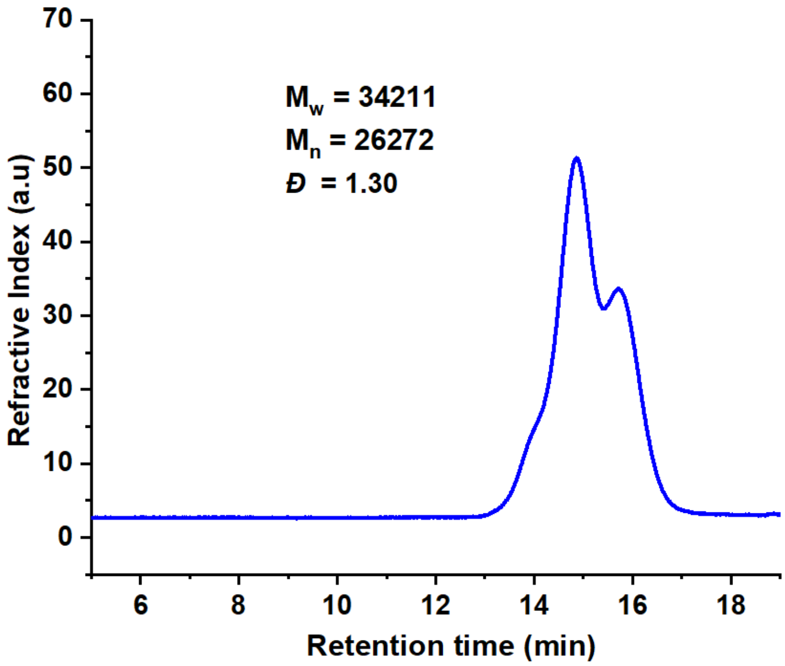

Figure S 69: GPC traces for polymonothiocarbonate (table 1, run 4).

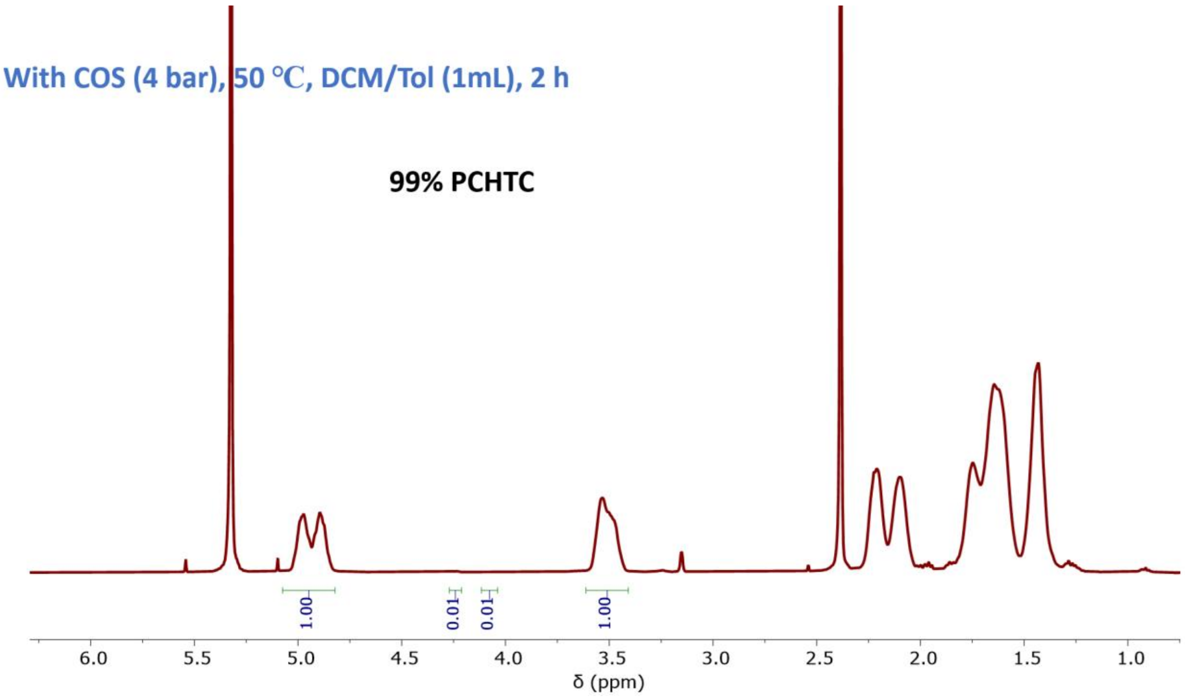

Figure S 70: Reaction mixture ^{1}H NMR of CHO with COS ($CDCl_3$, 400 MHz) (table 1, run 5).

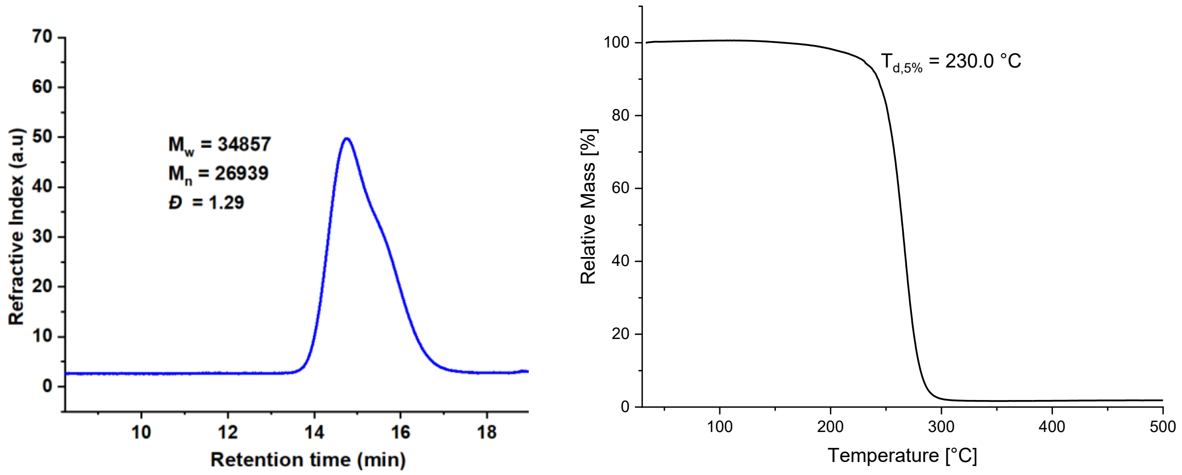

Figure S 71: GPC and TGA traces for polymonothiocarbonate (table 1, run 5).

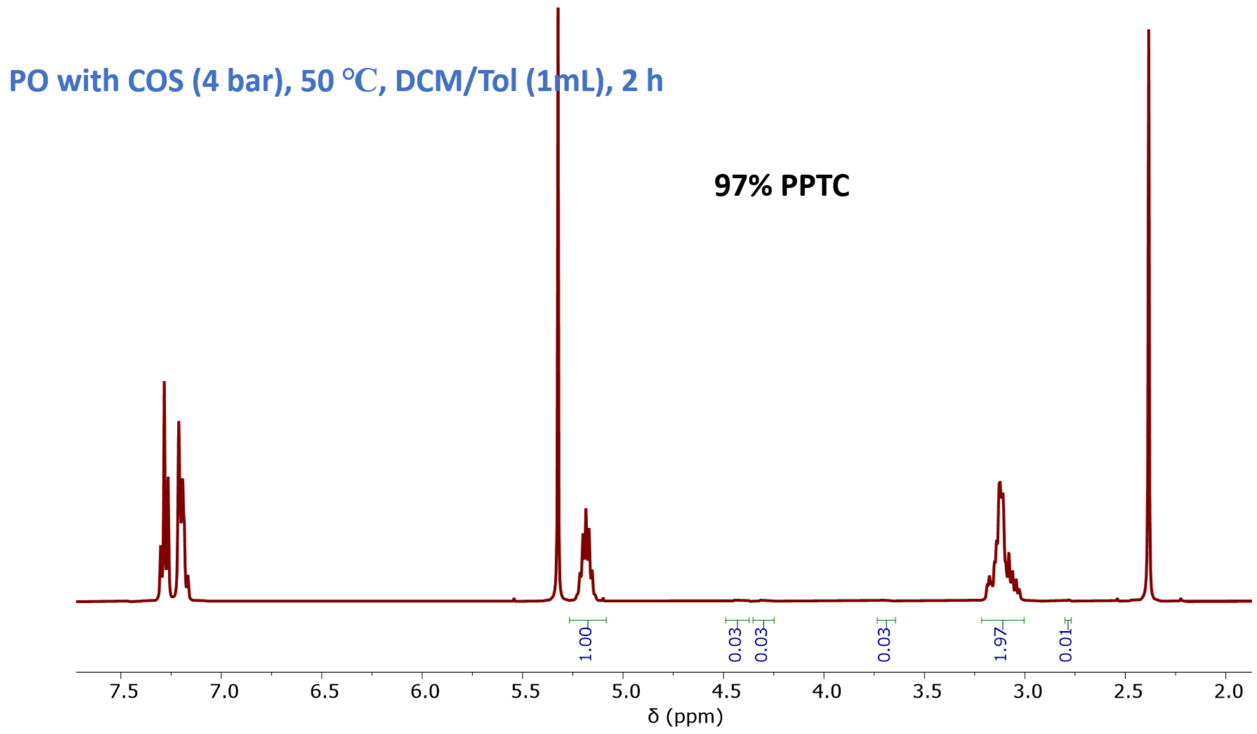

Figure S 72: Reaction mixture ^{1}H NMR of PO with COS ($CDCl_3$, 400 MHz) (table 1, run 6).

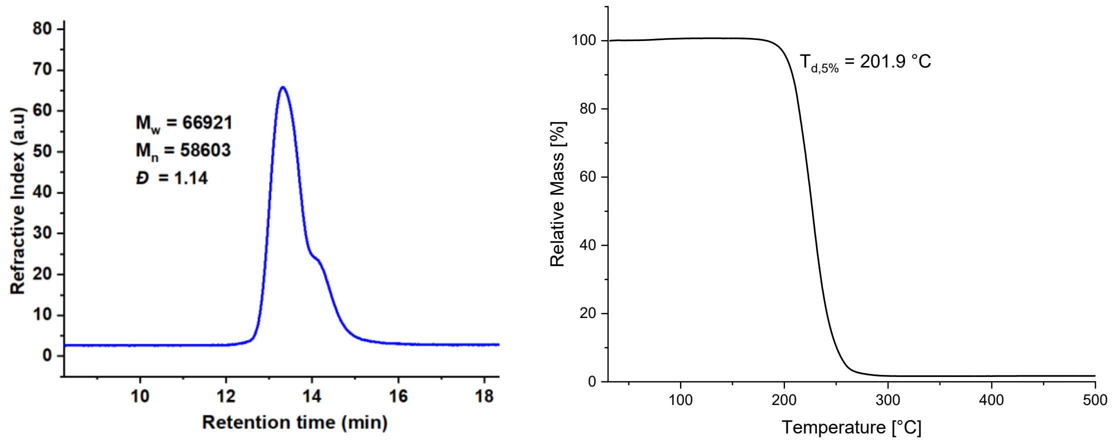

Figure S 73: GPC and TGA traces for polymonothiocarbonate (table 1, run 6).

PO with COS (4 bar), rt, DCM/Tol (1mL), 24 h

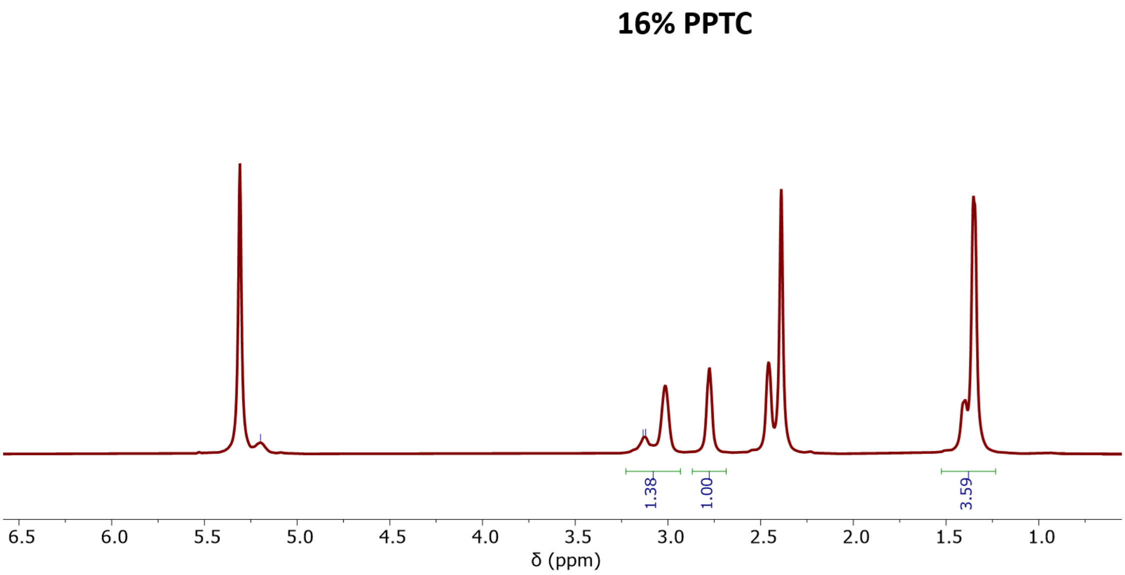

Figure S 74: Reaction mixture ^{1}H NMR of PO with COS ($CDCl_3$, 400 MHz) (table 1, run 7).

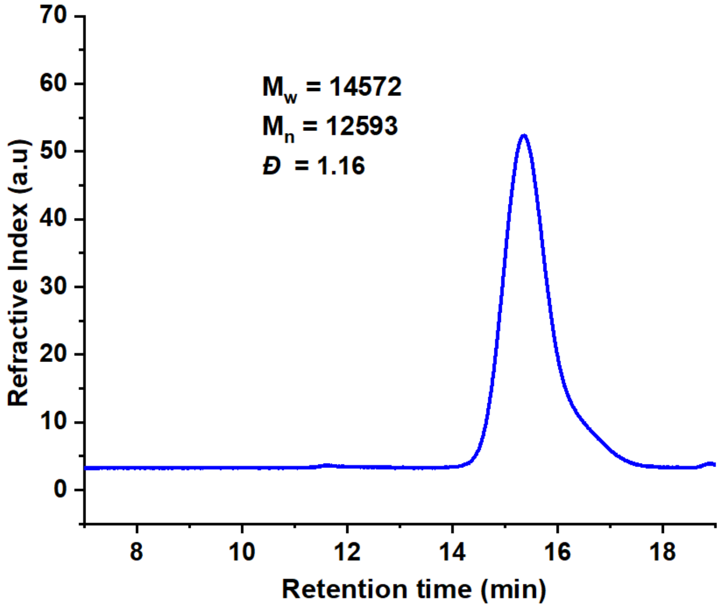

Figure S 75: GPC traces for polymonothiocarbonate (table 1, run 7).

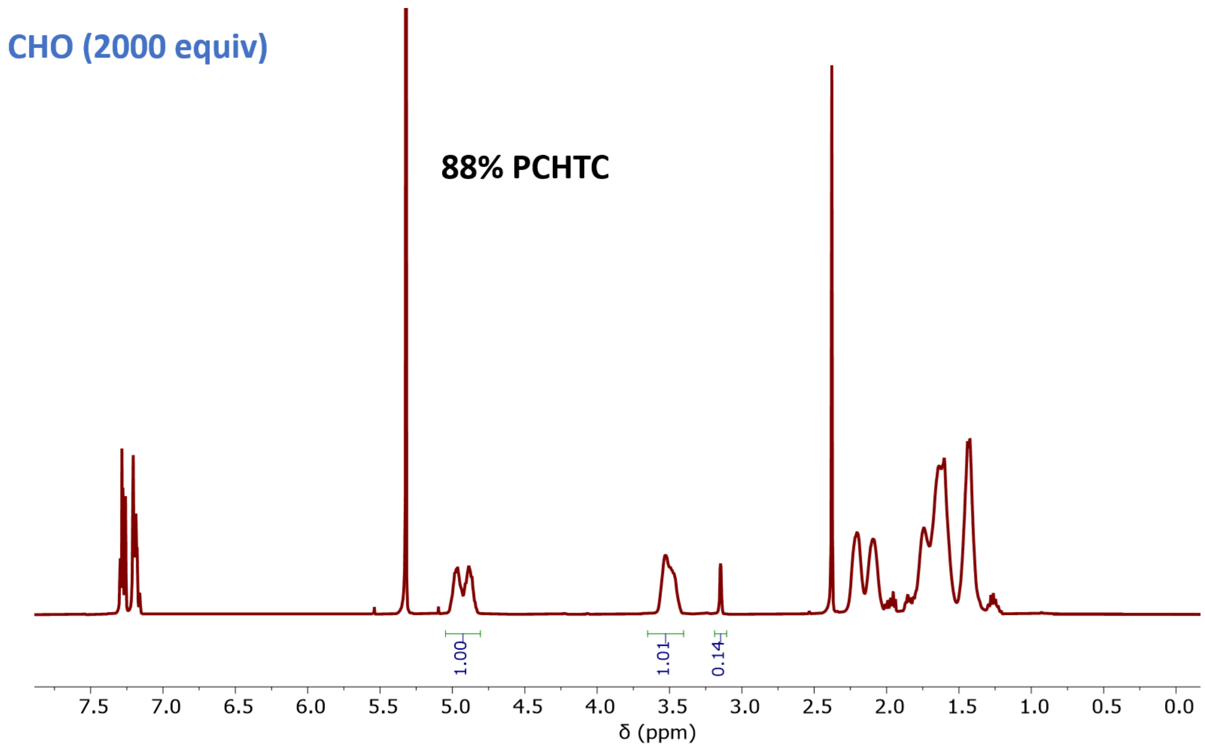

Figure S 76: Reaction mixture ^{1}H NMR of CHO with COS ($CDCl_3$, 400 MHz) (table 1, run 9).

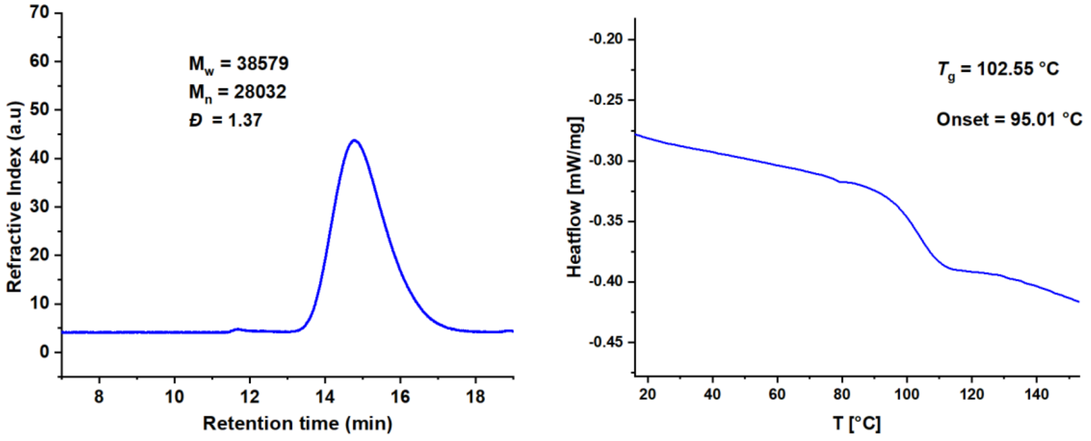

Figure S 77: GPC and DSC for polymonothiocarbonate (table 1, run 9).

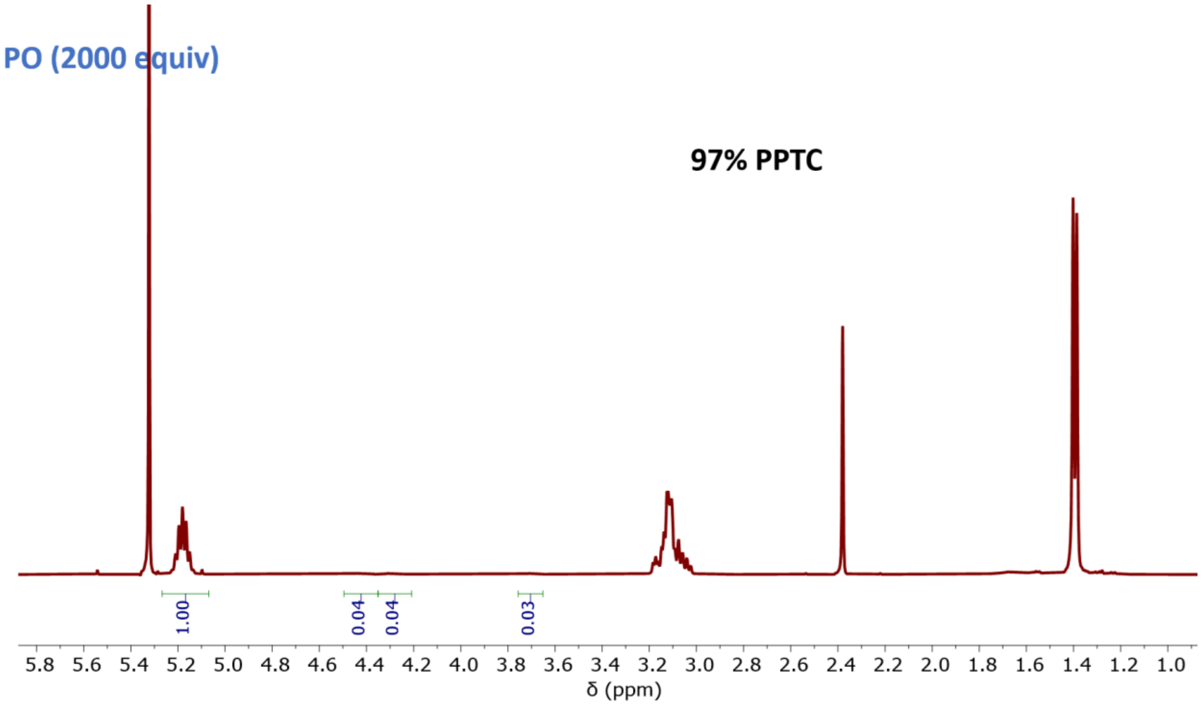

Figure S 78: Reaction mixture ^{1}H NMR of PO with COS ($CDCl_3$, 400 MHz) (table 1, run 8).

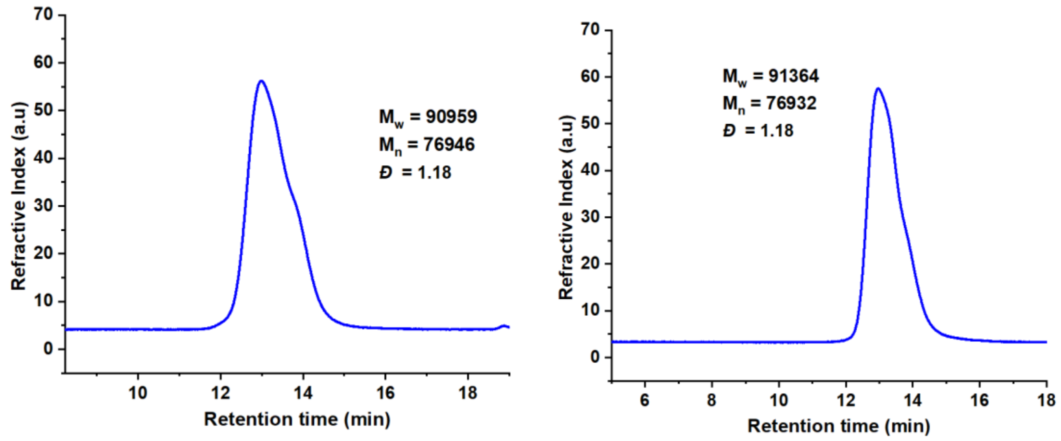

Figure S 79: GPC traces for polymonothiocarbonate (trail 1 & 2) (table 1, run 8).

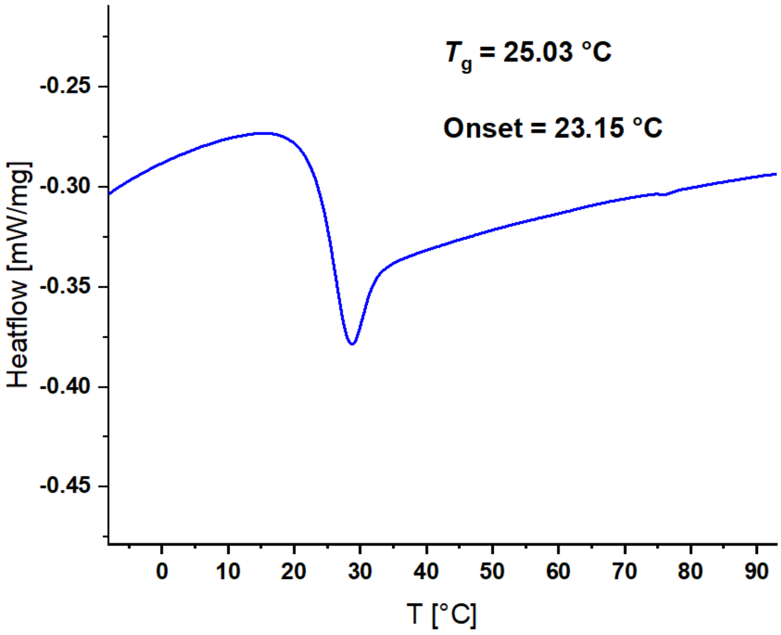

Figure S 80: DSC for polymonothiocarbonate (table 1, run 8).

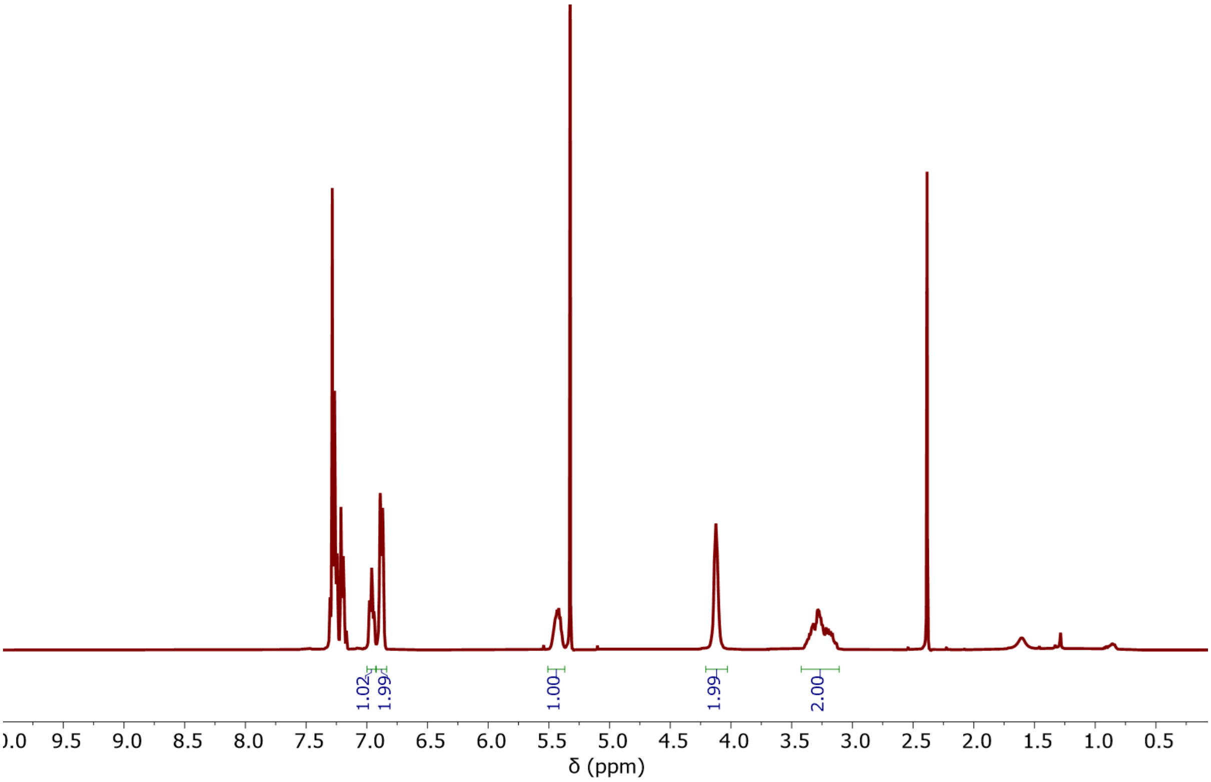

Figure S 81: Reaction mixture ^{1}H NMR of PGE with COS ($CDCl_3$, 400 MHz). (table 1, run 10).

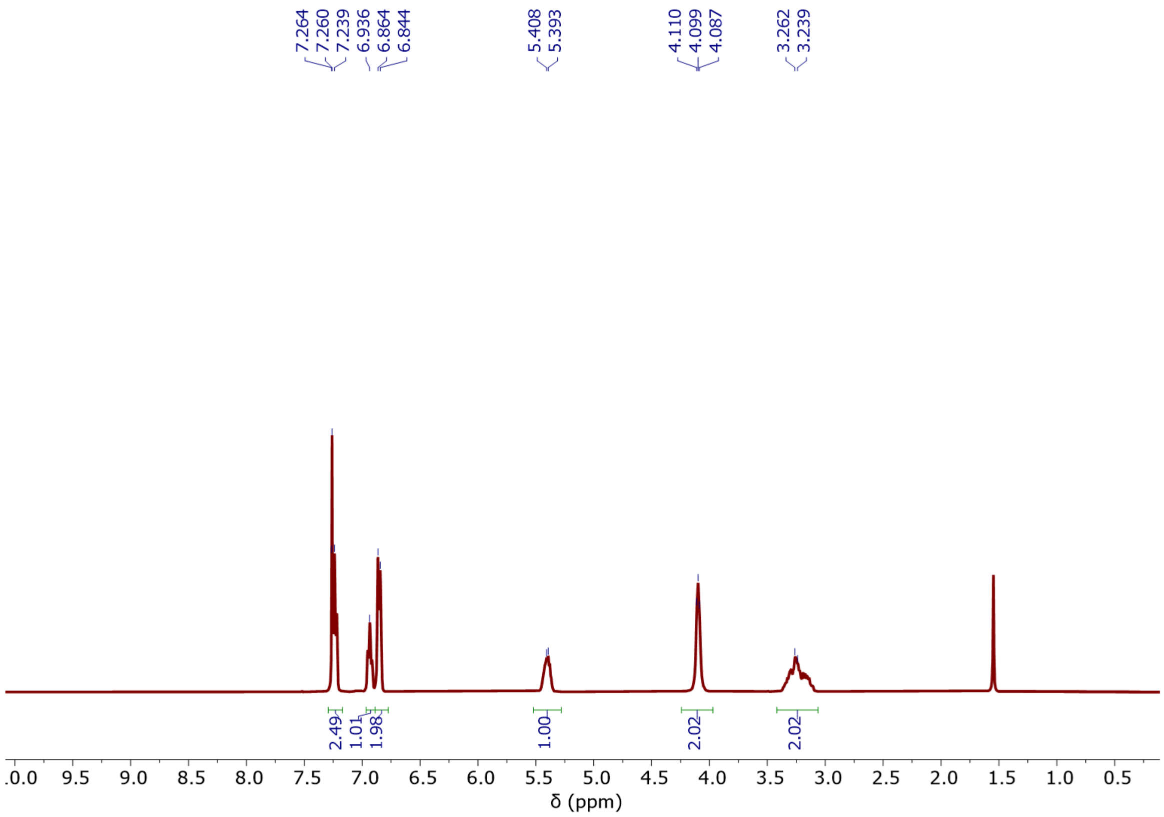

Figure S 82: ^{1}H NMR of polymonothiocarbonate obtained from PGE with COS ($CDCl_3$, 400 MHz) (table 1, run 10).

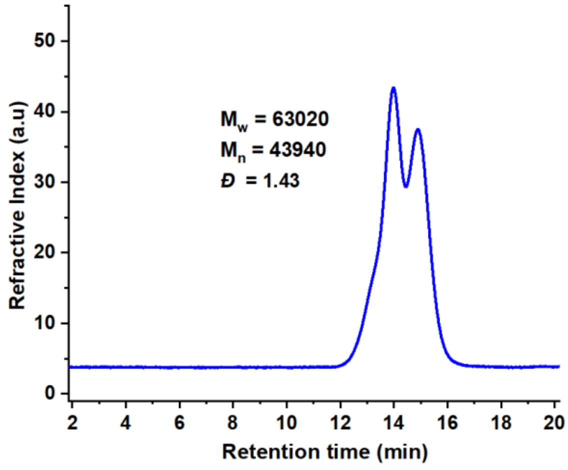

Figure S 83: GPC traces for PGE based polymonothiocarbonate. (table 1, run 10).

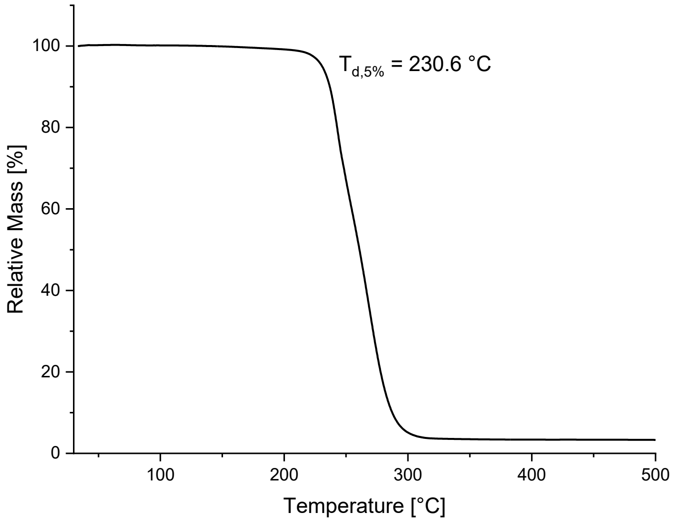

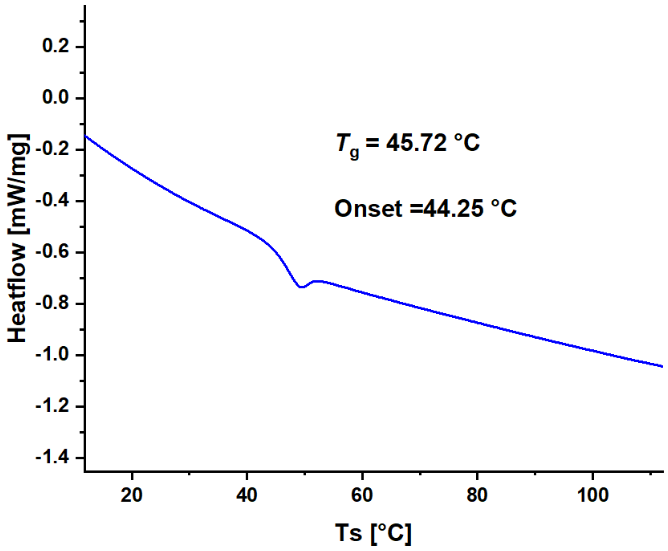

Figure S 84: TGA and DSC for PGE-polymonothiocarbonate. (table 1, run 10).

Section S4: Degradation studies

Procedure: 60 mg of powdered polymer sample was weighed into a vial containing a magnetic stirrer. 5 mL of the respective degradation solution was added into the vial. The vials were then placed in a heating block at 40 °C for 7 days. 5 mL of 1 M HCl was used for acid hydrolysis.[5] 5 mL of 5 wt.% NaOH in 60% ethanol solution was used for alkaline hydrolysis.[6] 5 mL of 7 M ammonia in methanol was used for aminolysis. After degradation, the polymer sample was filtered, washed twice and dried to determine the weight loss percentage before further analysis by GPC.

Table S8: Acid hydrolysis

Polymer	Weight loss (%)	M_n (kg/mol) (*Đ*) before	M_n (kg/mol) (*Đ*) after
CS_2/CHO	18.3	10.4 (1.7)	9.6 (1.7)
PhNCS/CHO	11.2	15.7 (1.5)	> 1 (2.9)
PTA/CHO	26.4	14.5 (1.7)	14.3 (1.6)
COS/CHO	5.1	17.7 (1.3)	18.0 (1.3)

Table S9: Alkaline hydrolysis

Polymer	Weight Loss (%)	M_n (kg/mol) (*Đ*) before	M_n (kg/mol) (*Đ*) after
CS_2/CHO	41.5	10.4 (1.7)	2.8 (2.7)
PhNCS/CHO	<1	15.7 (1.5)	14.5 (1.5)
PTA/CHO	61.9	14.5 (1.7)	1.8 (7.7)
COS/CHO	45.0	17.7 (1.3)	1.8 (6.4)

Table S10: Aminolysis

Polymer	Weight loss (%)	M_n (kg/mol) (*Đ*) before	M_n (kg/mol) (*Đ*) after
CS_2/CHO	>99	10.4 (1.7)	-
PhNCS/CHO	<1	15.7 (1.5)	14.9 (1.5)
PTA/CHO	>99	14.5 (1.7)	-
COS/CHO	81.5	17.7 (1.3)	5.8 (1.4)

Section S5: Computational Details

Gaussian16 Revision B.01 was used to perform DFT calculations with the B97D3 functional.[7] Geometry optimizations were performed with the def2-SVP basis set. [8,9] Frequency calculations of optimized structures were performed at the same level of theory (def2-SVP) to characterize the structures to be minima (no imaginary frequency) or transition states (one imaginary frequency) and yield thermodynamic corrections. Single point energy corrections were calculated at the ωB97XD /def2-TZVPP level of theory. It should be noted that all intermediates and transition states modelled in the computational investigations were calculated in the quartet ($^{4}Cr^{3+}$) spin-state.

Section S6: References

[1] M. R. Stühler, C. Gallizioli, S. M. Rupf, A. J. Plajer, *Polym. Chem.* **2023**, *14*, 4848–4855.

[2] J. Stephan, M. R. Stühler, S. M. Rupf, S. Neale, A. J. Plajer, *Cell Rep. Phys. Sci.* **2023**, *4*, 101510.

[3] L.-Y. Wang, G.-G. Gu, T.-J. Yue, W.-M. Ren, X.-B. Lu, *Macromolecules* **2019**, *52*, 2439–2445.

[4] J. Diebler, H. Komber, L. Häußler, A. Lederer, T. Werner, *Macromolecules* **2016**, *49*, 4723–4731.

[5] S. Hou, D. M. Hoyle, C. J. Blackwell, K. Haernvall, V. Perz, G. M. Guebitz, E. Khosravi, *Green Chem.* **2016**, *18*, 5190–5199.

[6] S. Ügdüler, K. M. V. Geem, R. Denolf, M. Roosen, N. Mys, K. Ragaert, S. D. Meester, *Green Chem.* **2020**, *22*, 5376–5394.

[7] M. J. Frisch et al., Gaussian16 Revision B.01 2016.

[8] F. Weigend, C. Hättig, H. Patzelt, R. Ahlrichs, A. Willets, *Phys. Chem. Chem. Phys.* **2006**, 8, 1057

[9] F. Weigend, R. Ahlrichs, K. A. Peterson, A. Bergner, *Phys. Chem. Chem. Phys.* **2005**, 7, 3297.
